# An extensive endoplasmic reticulum-localised glycoprotein family in trypanosomatids

**DOI:** 10.15698/mic2014.10.170

**Published:** 2014-10-01

**Authors:** Harriet Allison, Amanda J. O’Reilly, Jeremy Sternberg, Mark C. Field

**Affiliations:** 1 Division of Biological Chemistry and Drug Discovery, University of Dundee, Dundee, Scotland, DD1 5EH.; 2 School of Biological Sciences, University of Aberdeen, Aberdeen, AB24 2TZ, UK.

**Keywords:** Trypanosoma brucei, protein sorting, exocytosis, variant surface glycoprotein, endoplasmic reticulum, evolution, trypanosome

## Abstract

African trypanosomes are evolutionarily highly divergent parasitic protozoa, and as a consequence the vast majority of trypanosome membrane proteins remain uncharacterised in terms of location, trafficking or function. Here we describe a novel family of type I membrane proteins which we designate ‘invariant glycoproteins’ (IGPs). IGPs are trypanosome-restricted, with extensive, lineage-specific paralogous expansions in related taxa. In *T. brucei* three IGP subfamilies, IGP34, IGP40 and IGP48 are recognised; all possess a putative C-type lectin ectodomain and are ER-localised, despite lacking a classical ER-retention motif. IGPs exhibit highest expression in stumpy stage cells, suggesting roles in developmental progression, but gene silencing in mammalian infective forms suggests that each IGP subfamily is also required for normal proliferation. Detailed analysis of the IGP48 subfamily indicates a role in maintaining ER morphology, while the ER lumenal domain is necessary and sufficient for formation of both oligomeric complexes and ER retention. IGP48 is detected by antibodies from *T. b. rhodesiense *infected humans. We propose that the IGPs represent a trypanosomatid-specific family of ER-localised glycoproteins, with potential contributions to life cycle progression and immunity, and utilise oligomerisation as an ER retention mechanism.

## INTRODUCTION

*Trypanosoma brucei* is the causative agent of both Human African Trypanosomiasis and ‘nagana’ in livestock and game animals, important neglected tropical diseases afflicting much of sub-Saharan Africa 1. *T. brucei* has a complex life cycle, replicating within the mammalian bloodstream/lymphatic systems and central nervous system, as well as in various organs and tissues of the tsetse fly vector 2. During infection of the mammalian host, trypanosomes proliferate rapidly as morphologically slender forms. When a tsetse fly ingests trypanosomes, long slender forms are rapidly killed, whereas short stumpy forms are resistant and more efficiently establish infection 3. However, at the peak of each parasitaemic wave these forms mature into G_1/0_-arrested stumpy forms in response to stumpy induction factor, and partially differentiate for survival within the fly 4,5. This process may limit parasite populations, promoting chronic infection and preventing premature host death to augment transmission 3,6. Arrest of stumpy cells in G_1/0_ ensures that most developmental changes accompanying transmission into the tsetse fly coordinate with cell cycle re-entry.

Trypanosomes express high-abundance GPI-anchored proteins at the surface in both mammalian and insect hosts. In the mammalian form there are ~1 x 10^7 ^copies per cell of the variant surface glycoprotein (VSG), which represents ~10% of total protein. Switching between VSG variants permits escape from immune responses directed towards the previously expressed VSG, enabling chronic infection 7,8. VSG forms a dense, ~15 nm-thick surface coat, believed to protect underlying invariant epitopes from immune recognition. Due to comparatively low abundance compared to VSG, it has been challenging to identify additional membrane proteins biochemically. Evolutionary divergence adds additional complexity with defining surface or intracellular* trans*-membrane domain (TMD) proteins and membrane protein targeting signals. Knowledge of turnover mechanisms and functions is poor, despite the importance for maintaining the composition of the surface and intracellular organelles 9,10. Several large invariant TMD glycoprotein families, ISG60, ISG65 and ISG75, expressed exclusively in bloodstream form parasites, are known 11,12 and are termed invariant as they do not exhibit antigenic variation and are expressed at the surface and endosomal system by all strains of *T. brucei*. ISG65 and ISG75 contain large N-terminal extracellular domains, a single TMD and a small C-terminal cytoplasmic tail, but lack clear sequence homology with other proteins, beyond distant structural similarity to VSG 13. ISG75 mediates uptake of the trypanocide suramin and is likely a major protein degraded by the bloodstream form endosomal system 14,15. Other surface proteins have been described as have some components of the endomembrane system 16,17, but many surface or organellar proteomic datasets remain to be fully validated 18,19.

Targeting signals, recognised by multiple protein complexes within the endocytic and exocytic pathways, ensure accurate protein sorting. In animals and yeasts classic endocytic signals include tyrosine-based NPXY/YXXØ and dileucine-based [DE]XXXL[LI] or DXXLL signals, which are recognised by adaptin complexes^20^. Evidence for dileucine-dependent trafficking in *T. brucei* emerged through analysis of p67, the major trypanosomatid lysosomal glycoprotein; p67 contains two [DE]XXXL[LI]-type motifs, necessary and sufficient for lysosomal targeting, and likely mediated by AP-1 21,22. C-terminal KDEL signals (MDDL/KQDL in trypanosomes 23,24,25 retain lumenal chaperones in the ER 26,27, while dibasic residues near C- or N-termini (KKXX or KXKXXX) interact with COP-I, to facilitate retrieval from the Golgi apparatus 28. It is unclear if these latter signals operate in trypanosomes. Additionally, post-translational modification contributes to targeting. For example, ubiquitin (Ub) is involved in membrane trafficking and membrane internalisation 29, and the cytoplasmic domains of ISG65 and ISG75 are ubiquitylated, enabling sorting against a >100-fold VSG excess 14,30. Although a systematic analysis has not been reported, the number of GPI-anchors per complex has also been proposed to influence targeting 31,32,33.

With the exception of these examples, the signals required for targeting most *T. brucei *TMD proteins have not been defined, and the locations of the vast majority of TMD proteins are unknown. There is clear potential for divergence in both targeting signal primary structure (e.g., MDDL v KDEL) and in the molecular mechanisms underpinning trafficking, e.g., trypanosomatid-specific clathrin-associated proteins acting in endocytosis 34. To address this gap in our understanding, we used genome scanning to identify new families of type I TMD proteins. One, which we describe here, we termed invariant glycoproteins (IGPs) due to their expression from core genome regions distinct from the VSG antigenic variation telomeric expression sites and possessing similar size to ISGs. We found that IGPs are ER localised and retained by oligomerisation, independent of specific signals, within the cytoplasmic domain. Expression is upregulated in stumpy stage trypanosomes and IGPs are recognised by antibodies from infected humans, which suggests roles in both developmental transition and host immune response. Potential analogies to the ERGIC-53 ER lectins of mammalian cells are also discussed.

## RESULTS

### Identification of an extensive new type I TMD protein family

We have previously described the sorting itinerary and involvement in drug uptake of the invariant surface glycoprotein (ISG) family, and which also feature as important antigens both to the immune response and as diagnostics 14,15,30,35,36. As the vast majority of the predicted proteins within the genome with this topology have neither defined functions nor location, we set out to extend our knowledge of the signals required for sorting type I membrane proteins in the trypanosome cell by the identification of additional type I topology proteins.

To identify predicted polypeptides bearing an N-terminal ER targeting signal plus a predicted TMD, a series of computational filters were applied to the predicted proteome of the genome reference strain, *T. brucei *TREU 927 37 (Fig. S1). This procedure identified 208 open reading frames (ORFs) (accession numbers in Table S2), which were analysed by alignment and clustering by neighbour-joining (Fig. S2A). Our searches clearly under-sample the surface/endosomal proteome as only 33 of ~80 adenylate cyclases were recovered. Nevertheless, the screen was successful in identifying all known ISG/ISG-like ORFs. Significantly, a family of twenty hypothetical proteins, forming a distinct cluster, were identified and selected for further investigation.

### Evolution of the IGP family

**Figure 1:**
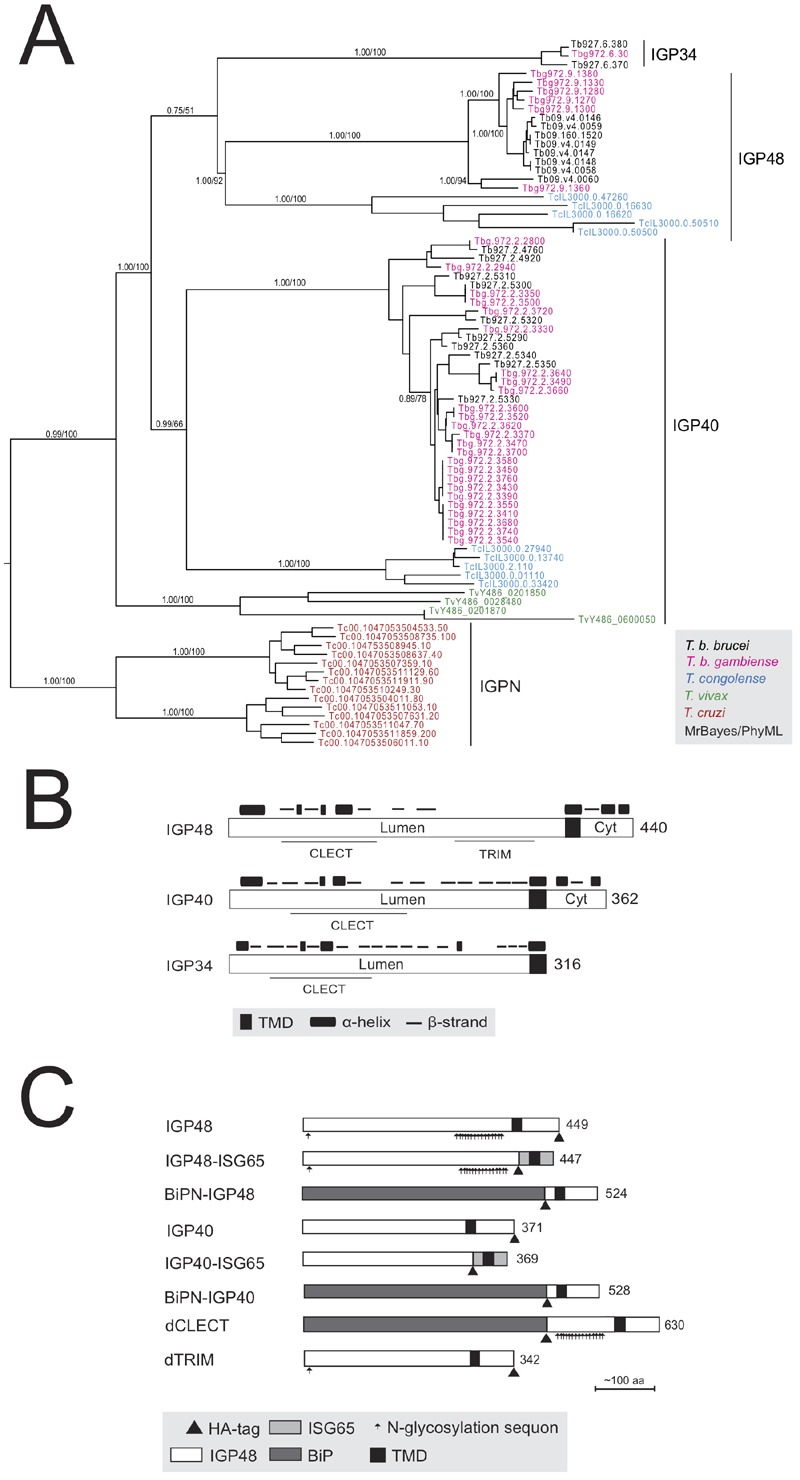
FIGURE 1: The invariant glycoprotein (IGP) family. **(A)** Phylogenetic reconstruction of the IGP family. The tree shown is the best Bayesian topology with branch support for important nodes indicated from both Bayesian and PhyML calculations. Clades are indicated by vertical bars. **(B)** Schematic diagram showing the secondary structure prediction and domain architecture of the IGP family. Lumen indicates the portion of the molecule predicted to be within the ER lumen and Cyt designates the predicted cytoplasmic domain. CLECT indicates C-type lectin domain. **(C)** Schematic structures of epitope-tagged and domain-swap IGP constructs used in this study. All constructs contain an HA tag as indicated by small triangle below the bar. **Abbreviations:** IGP48, full length IGP48; IGP48-ISG65, IGP48 ectodomain fused to ISG65 TMD and cytoplasmic domain; BiPN-IGP48, IGP48 ectodomain replaced with BiPN, retaining IGP48 TMD and cytoplasmic domain; IGP40, full length IGP40; IGP40-ISG65, IGP40 ectodomain fused to ISG65 TMD and cytoplasmic domain; BiPN-IGP40, IGP40 ectodomain replaced with BiPN, retaining IGP40 TMD and cytoplasmic domain; dTRIM, IGP48 with predicted trimerisation domain deleted.

We named this extensive protein family invariant glyco-proteins (IGPs), due to similarity to ISGs in overall predicted molecular weight, architecture, predicted topology, the presence of at least one N-glycosylation site and expression from housekeeping regions of the genome and not telomeric sites associated with antigenic variation. Twenty open reading frames encoding IGPs were identified in the *T. brucei* 927 genome (the reference genome strain), divided into three subfamilies based on sequence similarity (Fig. 1A). We designated these IGP34, IGP40 and IGP48 on account of the predicted molecular weights of their core polypeptides. All of the IGP open reading frames were recovered by the screen as additional BLASTp searches of the *T. brucei *genome failed to identify additional IGP sequences.

All twenty IGPs contain a predicted N-terminal signal and C-type lectin (CLEC) domain (Pfam PF00059), albeit with relatively weak similarity to the archetypal CLEC domain (1.24 x e^-4^) (Fig. 1B). Although sequence identity within each subfamily is high (85 to 100%), identity and similarity between IGP subfamilies is significantly lower, specifically IGP48 against IGP40 (25-30%) and IGP48 against IGP34 (50-60%). The most conserved region within the IGP family spans the predicted CLEC domain. Residues 247-336 of the IGP48 subfamily are similar to the trimerisation domain of the LpxA-like enzyme superfamily that occurs within many bacterial transferases, including UDP-N-acetylglucosamine acyltransferase (Pfam PF00132). This region is composed of multiple DENTTV repeats, which contain an N-glycosylation sequon and a potential β-turn, the latter suggesting that they may be occupied by an N-glycan.

IGP genes are present in all salivarian trypanosome genomes, plus *Leishmania, Leptomonas, Phytomonas* and the American trypanosome *T. cruzi* (Fig. S2B and S2C). Many of the IGP proteins are encoded within tandem gene arrays, and hence independent array expansion and contraction is a likely mechanism for the apparent plasticity between taxa of IGP copy number. Two IGP orthologues are also present in the recently sequenced *T. grayi* genome 38. Significantly, no evidence for IGP-related genes was found in non-trypanosomatid Excavata genomes, specifically *Naegleria gruberi*, *Trichomonas vaginalis* and *Giardia intestinalis*, or non-excavate taxa (data not shown).

Phylogenetic analysis established the evolutionary history of the IGP family across the kinetoplastida (Fig. S2A, S2B, S3A and S3B). The IGP family possesses no obvious homology to the ISGs, and phylogenetic reconstruction of all ISG, ISG-like and IGP predicted protein sequences in *T. brucei* robustly reconstructs these as independent clades (1.0/98 Mr Bayes posterior probability/PhyML bootstrap support). Further, a scan of all eukaryotic genomes only identified IGP-related sequences in kinetoplastids, indicating the presence of a trypanosomatid-specific defining domain, which has been assigned a unique pfam ID: Pfam PF16825 (DUF5075). Expansions are common within trypanosomes and *Leishmania*, but interestingly these expansions are specific to each lineage (Fig. S2C). Moreover, the number of IGP paralogs tends to be lower in the *Leishmanias*, and the phylogeny indicates that the origin of several IGP paralogs predates speciation of the *Leishmania* and *Phytomonas* lineages. Significantly, *Angomonas deanei *and *Strigomonas culicis*, both monogenous parasites (as opposed to the digenous *Leishmanias* and trypanosomes) have only two IGP paralogs each, and *Leptomonas pyrrhocoris*, also monogenous, has just three IGP paralogs. These data suggest that the IGP family may have expanded in response to the greater burdens of infecting multiple hosts, and, moreover, that this process occurred independently on several occasions. Overall, these data suggest that the IGP gene family arose during the origin of the Kinetoplastida, and clearly predating the ISGs which are restricted to salivarian trypanosomes.

### IGPs are developmentally regulated

**Figure 2:**
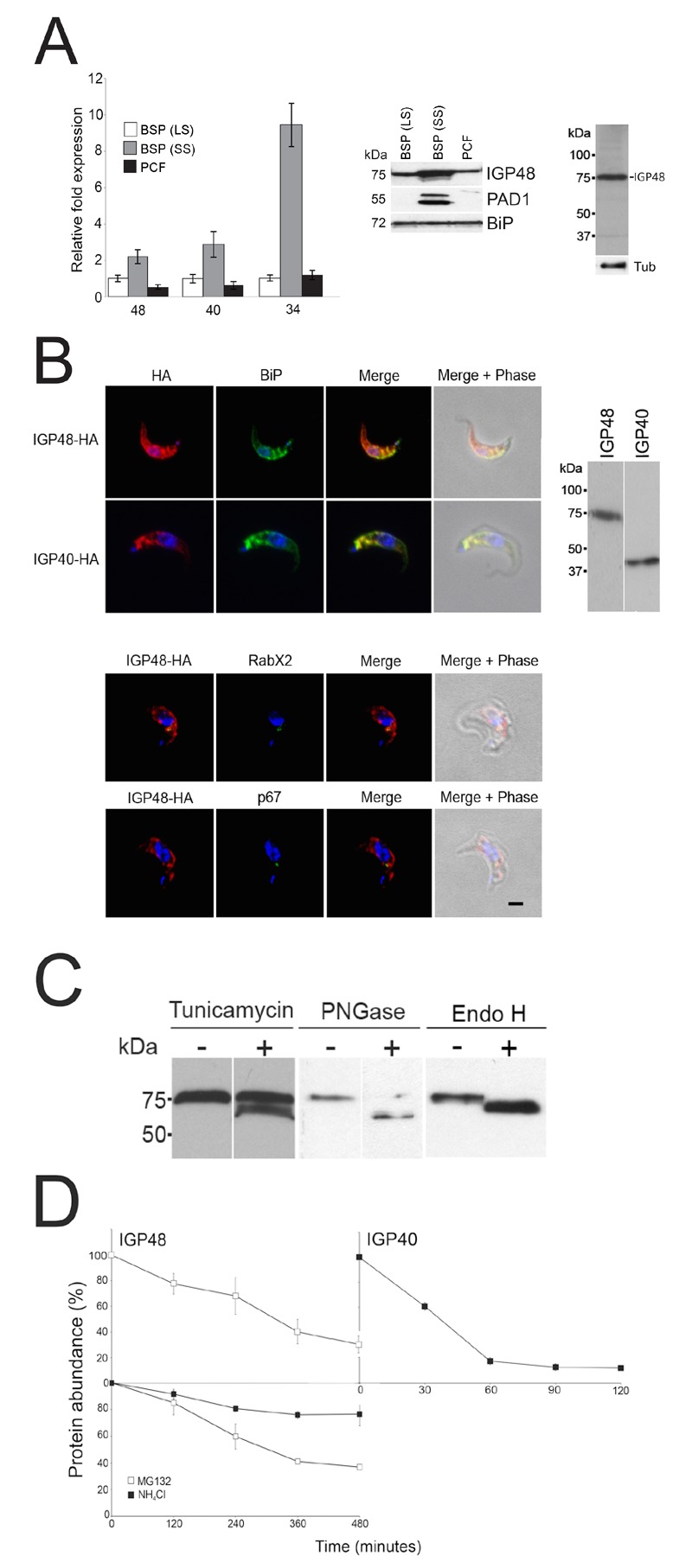
FIGURE 2: The IGP family are developmentally regulated ER proteins. **(A)** Copy numbers of IGP48 39, IGP40 40 and IGP34 34 mRNAs measured by qRT-PCR, in different life cycle stages, normalised to long slender BSF mRNA levels at 1.0. Error bars denote standard errors of the mean from triplicate measurements on independent RNA samples. Western blot of trypanosome whole cell lysates using anti-IGP48 affinity-purified antisera raised against *E. coli*-expressed recombinant protein at 1:100 dilution. The blot was re-probed for PAD1 (protein associated with differentiation 1), and which is specifically unregulated in the stumpy bloodstream form, to validate the short stumpy lysate. Rightmost; whole cell lysate probed with anti-IGP48 antisera to validate specificity. **Abbreviations: **BSF (LS), long slender bloodstream form; BSF (SS), short stumpy bloodstream form and PCF, procyclic culture form. **(B)** Intracellular localisation of IGP48-HA and IGP40-HA in BSF cells under permeabilised conditions, and detected with anti-HA antibody (red). **Top panel:** co-staining with anti-TbBiP (green). **Lower panels:** co-straining with anti-TbRabX2 and anti-p67 (green) using confocal microscopy. Bar = 2µm. Inset: expression of IGP48-HA and IGP40-HA in 427 BSF cells detected by Western blotting using anti-HA antibody. **(C)** Digestion of IGP48 with PNGase F or Endo H, or treatment with tunicamycin results in a large molecular weight shift. IGP48 was detected in fractionated lysates using anti-HA antibody. **(D)** Turnover of IGP48 and IGP40. Quantification of anti-HA reactivity in lysates of cells expressing IGP48-HA and IGP40-HA following inhibition of protein synthesis with cycloheximide. Error bars represent the standard deviation and values were normalised against a loading control, BiP (n = 3).

To gain insights into the functions of the IGP proteins we initially examined their expression in the major accessible life stages of *T. brucei. *Long slender and PCF stage material was obtained from *in vitro* culture and short stumpy mRNA and protein lysates were from infected mice (kind gift of Keith Matthews, University of Edinburgh). The mRNA abundance of each IGP subfamily was assessed using primers designed to amplify all copies within a subfamily (Fig. 2A). All three IGP subfamilies are significantly up-regulated in the short stumpy bloodstream stage, most noticeably IGP34 (~10-fold compared to long slender BSF and PCF), whereas mRNA levels in the long slender BSF and PCF are relatively similar to each other.

An affinity purified rabbit anti-IGP48 polyclonal antibody was used to probe whole-cell lysates and detected a single band at 75 kDa, significantly greater than the predicted core polypeptide molecular weight of 48 kDa, likely due to post-translational modification. Probing whole-cell lysates from these same life cycle stages with anti-IGP48 confirmed that IGP48 is indeed up-regulated in the short stumpy stage by ~4-fold at the protein level compared to long slender and PCF cells (Fig. 2A). As a control, expression of PAD1 (protein associated with differentiation 1) was also monitored using a polyclonal antibody (again, kind gift of Keith Matthews). PAD1 immunoreactivity was only observed in the short stumpy bloodstream cell lysates.

### IGP40 and IGP48 are N-glycosylated and locate to the ER

To further understand IGP function, we chose to determine the location of the IGPs. C-terminally HA epitope-tagged IGP48, IGP40 and IGP34 constructs were expressed in BSF 427 trypanosomes (Fig. 2B). One representative of each of the IGP48 (Tb09.v4.0147), IGP40 (Tb927.2.5330) and IGP34 (Tb927.6.380) families were selected for further study on account of demonstrating high identity with the majority of the remaining IGP paralogs in that subfamily, and hence most likely to report on the location of the remaining family members. Positive transformants were co-stained with antibody to TbBiP, an ER-resident protein 41 and HA (Fig. 2B). Fluorescence for IGP40, IGP48 and TbBiP exhibited extensive overlap, indicating that IGP40 and IGP48 have a substantial presence at the ER. Despite multiple attempts, no signal could be detected by either immunofluorescence or Western blotting for IGP34. To determine if IGP48 localised to other compartments within the cell, BSF cells expressing IGP48-HA were co-stained with TbRabX2, a Golgi marker 42, and p67, a lysosomal marker 43. Results indicated no obvious presence in the lysosome, but potentially some presence at the Golgi complex at steady state, but which is comparatively minor compared to the extensive ER population.

Western blots of cell lysates expressing IGP40-HA or IGP48-HA ectopic constructs suggested that IGP48 migrates with an observed molecular weight ~25 kDa greater than the predicted core polypeptide, whereas the observed molecular weight of IGP40 was similar to its predicted polypeptide core, suggesting that IGP48 undergoes extensive post-translational modification. Given fifteen predicted N-glycosylation sites in the IGP48 TRIM domain, it is probable that IGP48 bears the additional weight of multiple N-glycans (Fig. 1C). To assess this possibility, cells were cultured in the presence of tunicamycin or whole cell lysates were treated with peptide N-glycanase (PNGase F) or endoglycosidase H (Endo H) prior to analysis by Western blotting (Fig. 2C). Treatment with tunicamycin and PNGase F resulted in increased mobility of ~15 kDa, whereas Endo H digestion led to a mobility shift of only ~10 kDa. This suggests that IGP48 contains a mixture of Endo H-sensitive oligomannose glycans and Endo H-resistant paucimannose and/or complex N-glycans, the latter a result of processing in the Golgi complex. Therefore, the presence of the vast majority of IGP48 in the ER at steady state suggests the possibility of cycling through the Golgi complex to generate Endo H-resistant glycans. The remaining ~10 kDa modification may be due to the presence of other modifications, for example O-glycans or PNGase F-resistant N-glycans, aberrant migration on SDS-PAGE or a result of oligomerisation, as investigated below. Interestingly, IGP48 also binds tomato lectin (TL), which is specific for N-linked glycans containing three or more linear repeats of N-acetyllactosamine, although recent evidence suggests that TL can also bind paucimannose structures; either structural class would reside in the Endo H-resistant N-glycan fraction 38,40. Removal of the trimerisation domain, in which all predicted N-glycosylation sites are located, ablated TL binding, indicating that the TRIM domain harbours these N-glycans (Fig. S4). Furthermore, these data confirm the predicted topology of IGP48 as a type I membrane protein, with the N-terminus accessible to the glycosylation machinery and hence located within the ER lumen.

### IGP48 is degraded in a low pH compartment

The presence of IGP40 and IGP48 predominantly within the ER, but also likely trafficking through the Golgi complex, suggested that either these proteins are degraded directly from the ER by an ER-associated degradation-related mechanism 44 or that they progress to post-Golgi compartments and are degraded in terminal endosomal compartments. Turnover of IGP48-HA and IGP40-HA was monitored following treatment of cells with cycloheximide and Western blotting with anti-HA antibody (Fig. 2D). IGP48-HA and IGP40-HA differed significantly in their stability, with a half-life of ~6 hours for IGP48-HA and ~30 minutes for IGP40-HA. Turnover of IGP48-HA was further analysed by inhibition of lysosomal functions with the weak base ammonium chloride or a proteasome inhibitor, MG132, to inhibit ER-associated degradation. Ammonium chloride clearly delayed IGP48-HA degradation with ~80% of protein remaining after 4 hours compared to ~40% in untreated cells, but no significant effect was seen with the proteasomal inhibitor. Together with the absence of a clear lysosomal pool of IGP48 (Fig. 2B), these data suggest that IGP48 is delivered to the lysosome but rapidly degraded once attaining the terminal endosomal compartment. Overall, despite a predominant presence within the ER, IGP48 may exit the ER, consistent with both the presence of Endo H-resistant N-glycans and ammonium chloride-sensitive turnover.

### IGP proteins are required for normal proliferation

RNAi knockdown was used to investigate the putative roles and importance to viability of the IGP gene products, using constructs designed to suppress all members of each subfamily. Knockdown was subfamily specific, with no cross-suppression against other subfamilies detected by qRT-PCR (data not shown).

**Figure 3:**
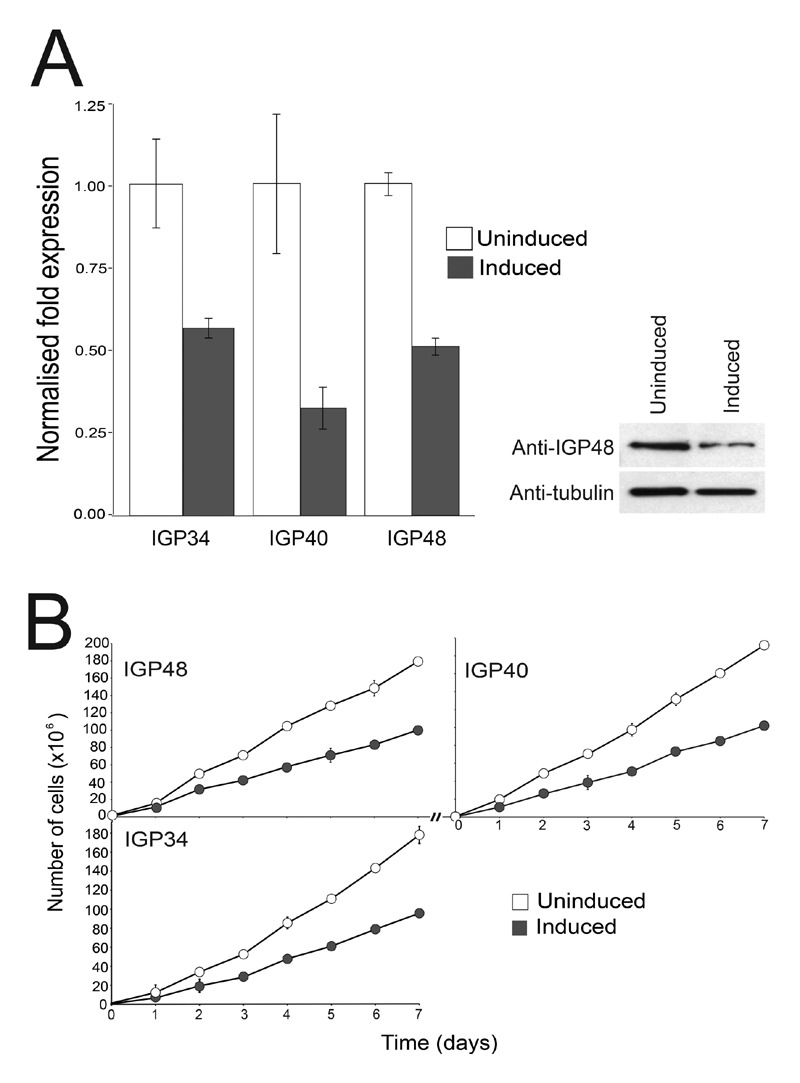
FIGURE 3: IGP40 and IGP48 are required for normal cell proliferation. **(A)** qRT-PCR of control and tetracycline-induced RNAi lines for IGP34, IGP40 and IGP48 24 hours post-induction (n = 3). **Right panel** shows a Western blot of IGP48 following RNAi induction after 24 hours with β-tubulin as loading control. **(B) **Growth curves of control and tetra-cycline-induced RNAi lines for IGP34, IGP40 and IGP48, showing a growth defect within 24 hours post induction. Representative results for one of two clonal cell lines for each construct studied are shown (n = 2) (all subsequent experiments were performed using this cell line). Cultures were diluted daily to maintain cell densities between 10^5^ and 2 × 10^6^ cells/ml, and a cumulative pseudo-growth curve is shown. Counts were carried out in triplicate, error bars represent standard error of the mean.

Specific down-regulation at the mRNA level of the three IGP subfamilies was confirmed by qRT-PCR after 24 hour induction (Fig. 3A) and indicated that mRNA levels for the IGP34, IGP40 and IGP48 induced cell lines were reduced by ~50%, 65% and 50% compared to uninduced cells, respectively. Depletion of IGP48 at the protein level by ~50% was confirmed by Western blotting of whole cell lysates from induced cells 24 hours post-induction and probed with anti-IGP48 antibody (Fig. 3A, right panel).

Proliferation defects were observed following 24 hours of RNAi induction and continued for several days (Fig. 3B). These data suggest that each IGP subfamily is required for normal cell growth and proliferation, and that despite their similarities, these gene families have non-redundant functions. In addition, after two to three days, cells with multiple nuclei and kinetoplasts were observed (Fig. S5). This phenotype emerges following knockdown of a range of resident ER proteins, as well as many other genes in *T. brucei *45. However, this minor cytokinesis defect, as demonstrated by an increase in cells with two or more nuclei, is most likely a secondary defect as it occurs following a significant period of proliferative impact. Therefore, each IGP subfamily is independently required for normal cellular physiology.

### The IGP48 lumenal/ecto-domain is required for ER retention

To determine which IGP protein regions are required for targeting and/or ER retention a panel of deletion and domain-swap constructs was created (Fig. 1C). We chose to focus on IGP40 and IGP48 due to our inability to localise IGP34, and hence lack of information on the location of members of this subfamily. We produced constructs to investigate the presence of targeting information in the C-terminal domains of IGP40 and IGP48 by replacing these with the equivalent portion of ISG65, which in the native context, or when fused to the N-terminal domain of BiP (BiPN), support efficient trafficking to the cell surface and endosomal targeting (IGP40-ISG65 and IGP48-ISG65). To investigate lumenal/ecto-domain targeting contributions, BiPN was fused to the IGP40 and IGP48 TMD, including a short spacer region from the lumenal/ecto-domain to ensure preservation of correct membrane topology (BiPN-IGP40 and BiPN-IGP48). BiPN contains essentially no targeting information and is normally rapidly secreted 35, and has only a very modest impact on the targeting or turnover of ISG65 and ISG75 fusion constructs, where the cytoplasmic signals contain the major targeting determinants 14,30,35.

**Figure 4:**
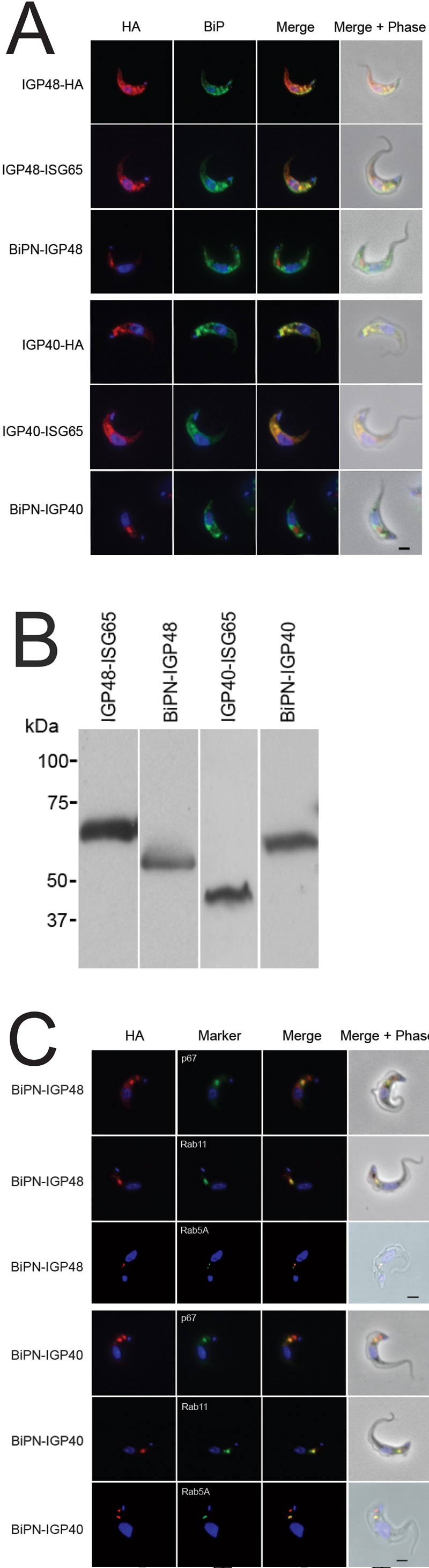
FIGURE 4: Localisation of IGP sorting signals. **(A)** Immunofluorescence demonstrating the locations of IGP constructs in BSF trypanosomes. HA-tagged constructs were detected in permeabilised cells with anti-HA antibody (red). The ER was stained with anti-TbBiP (green) and DNA stained with DAPI (blue). BiPN constructs, in which the lumenal/ecto-domain is replaced by the BiP ATPase domain, no longer co-localise with TbBiP and so are not retained within the ER. **(B)** Expression of IGP40-HA and IGP48-HA in 427 BSF cells detected by Western blotting using anti-HA antibody. Note that the BiPN chimeras migrate slower than predicted from their molecular weight, an observation that is consistent with the behaviour of BiPN-ISG65 chimeras reported previously 30. **(C)** Location of BiPN constructs was determined by co-localisation with p67, Rab5A or Rab11, markers for the lysosome, early or recycling endosomes respectively (green). BiPN-IGP40 and BiPN-IGP48 both demonstrate significant overlap with all three intracellular markers. Scale bar 2 µm.

IGP40-ISG65 and IGP48-ISG65 both co-localised with BiP, suggesting an ER location indistinguishable from full-length IGP40 and IGP48 proteins (Fig. 4A), and indicating that the IGP TMD and cytoplasmic domains do not possess essential ER retention signals. By contrast, replacement of the IGP40 or IGP48 lumenal/ecto-domain with BiPN (BiPN-IGP48 and BiPN-IGP40) resulted in loss of co-localisation with BiP, suggesting that these constructs now exit the ER. A Western blot of cell lysates expressing constructs confirmed correct incorporation of the various tags and chimeras (Fig. 4B). BiPN-IGP40 and BiPN-IGP48 were localised to distinct puncta between the nucleus and kinetoplast; co-localisation with p67, Rab11 and Rab5A demonstrated that these correspond to several endosomal compartments, including the lysosome, recycling endosomes/exocytic carriers and early endosomes, indicating inefficient retention by the ER and trafficking onto the degradative arm of the endosomal system (Fig. 4C). Together, these data indicate that the basis for ER retention resides within the IGP lumenal/ecto-domain, and not the TMD or cytoplasmic domain.

### The IGP40/IGP48 lumenal/ecto-domain is required for retention by the cell

**Figure 5:**
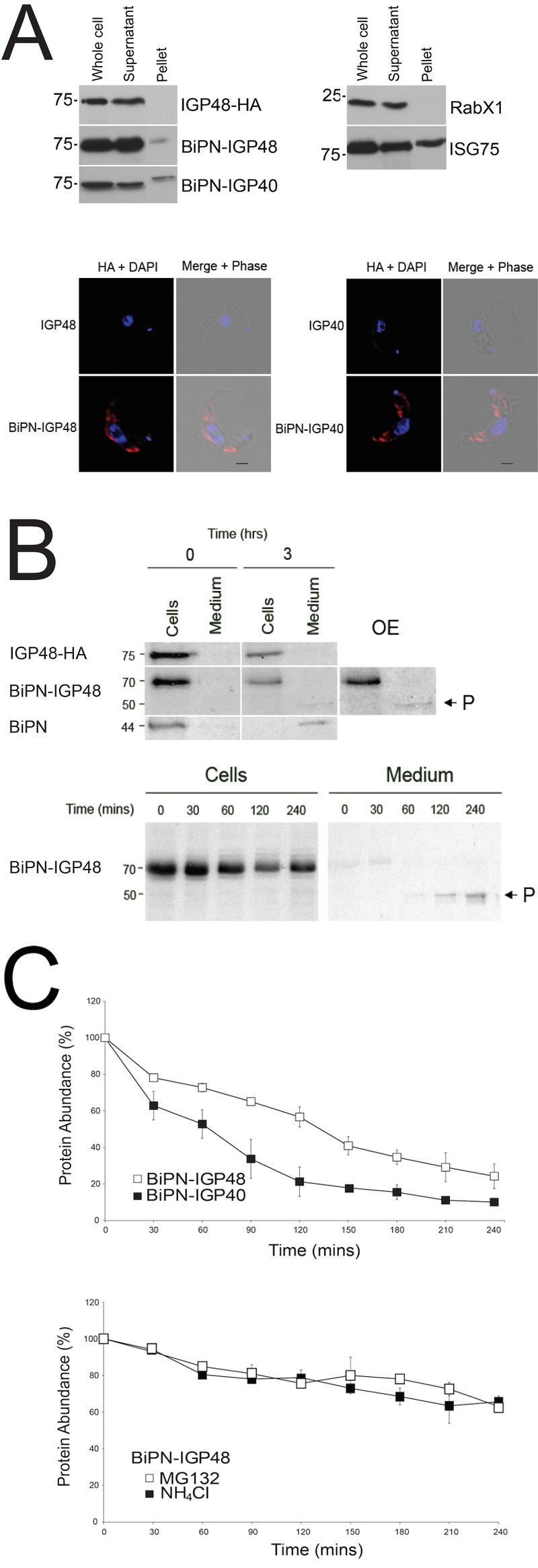
FIGURE 5: The IGP ectodomain is required for retention. **(A)** Western blot analysis of surface-biotinylated (Pellet) and non-biotinylated (Supernatant) cells to detect surface-exposed IGP proteins and chimeras. Blots were also probed for an intracellular (TbRabX1) and surface (ISG75) control, to demonstrate that cells are intact and that surface components are successfully biotinylated. Note that the Pellet lanes have been moved in Photoshop simply for clarity and no other manipulation has taken place. Surface presence of BiPN-IGP48 and BiPN-IGP40 was further demonstrated by confocal microscopy. Non-permeabilised cells were stained with anti-HA antibodies (red) and for DNA (blue). Scale bar = 1 μm. **(B)** Kinetics of protein secretion. BSF trypanosome cells expressing IGP48-HA, BiPN-IGP48 and BiPN were pulse-labeled with ^35^S-Met/Cys for 15 minutes and then chased for 3 hours. At 0 and 3 hours cultures were separated into cell and medium fractions. Labeled proteins were immunoprecipitated with anti-HA and separated by SDS-PAGE. OE, OverExposure to reveal a 70 kDa proteolytic fragment (P) cleaved from BiPN-IGP48. **Lower panel:** Detailed kinetics of BiPN-IGP48 secretion. Cells were pulse-labeled with ^35^S-Met/Cys for 15 minutes and at the indicated chase times, aliquots were treated as described above. The proteolytic BiPN-IGP48 50 kDa fragment (P) appears in the medium after 1 hour. **(C)** **Left panel:** Turnover kinetics of BiPN-IGP48 (open symbols) and BiPN-IGP40 (closed symbols) was determined by blocking protein synthesis with cycloheximide and detecting residual protein with anti-HA antibodies. **Right panel:** Turnover is sensitive to inhibition by 10 µM MG-132 (open symbols) or 20 mM NH_4_Cl (closed symbols). Results were normalised to 100% at t = 0. The graph represents the mean of two independent experiments, with the standard error of the mean indicated. Numbers to the left of some panels indicate the positions of co-migrated molecular weight standards and are in kDa.

The presence of BiPN-IGP fusion proteins in Rab11-positive compartments suggests that these chimeras may be exported to the cell surface and/or be delivered into endosomes. To investigate the fate of post-ER IGP chimeras, cells expressing BiPN-IGP40, BiPN-IGP48 or full-length IGP48 were surface-derivatized with biotin using a membrane-impermeant biotinylation reagent 30 and lysates fractionated into biotinylated (surface-accessible) and non-biotinylated (internal) fractions using streptavidin agarose. The relative levels of the constructs recovered in each fraction were revealed by Western blotting with anti-HA antibody (Fig. 5A). IGP48-HA was recovered entirely within the underivatized pool, suggesting that the protein was inaccessible to biotinylation, whereas a small fraction of both BiPN-IGP constructs was biotin-accessible. The distribution of native ISG75, which has a presence both at the surface and in intracellular organelles, was faithfully reflected by the biotinylation analysis 14, while RabX1, a cytoplasmic protein, was not detected in the biotinylated fraction, indicating that the cells remained intact during the procedure. To confirm that BiPN-IGP constructs are present on the cell surface, optical sections of non-permeabilised cells were taken using confocal microscopy (Fig. 5A, lower panel). In agreement with previous data, no evidence for IGP40 or IGP48 on the cell surface was obtained, but by contrast, both BiPN-IGP constructs are clearly seen at the cell periphery in central confocal sections, consistent with a surface location.

To determine whether BiPN-IGP chimeras are secreted from the cell when they reach the surface, radio-immunoprecipitations were performed on cells and culture media for BiPN-IGP48, IGP48 and BiPN 35. Cells were pulse-radiolabeled with ^35^S-methionine and chased, after which labelled proteins were recovered by immunoprecipitation with anti-HA antibody (Fig. 5B). IGP48 remained within the cell fraction even up to three hours, indicating that essentially all of the protein was retained by the cell. By contrast, after three hours a ~50 kDa proteolytic fragment (P) of BiPN-IGP48 was detectable in the medium, which clearly retains the HA-epitope (Fig. 5B). Essentially all BiPN was secreted as expected. Very small quantities of intact BiPN-IGP48 are shed into the medium up to 30 minutes post-chase, but the 50 kDa fragment was observed in increasing quantities up to three hours, likely reflecting a true secretion event. The molecular weight of this fragment suggests that cleavage occurs at a point immediately N-terminal to the *trans*-membrane domain of IGP48, and is consistent with BiPN-IGP48 attaining the cell surface. However, both the pulse-chase and biotinylation data indicate that the vast majority of the protein is retained within the cell.

The half-lives of BiPN-IGP48 and BiPN-IGP40 were determined to be approximately two hours and one hour respectively (Fig. 5C). This significantly fast rate of degradation and the presence of some of the BiPN-IGP constructs within the lysosome (Fig. 4B) suggests that these constructs are trafficked to this organelle. Turnover of BiPN-IGP48 was also analysed in the presence of MG132 or NH_4_Cl, with both significantly increasing the half-life of this construct, so that after four hours ~70% of protein remained, compared to ~30% in untreated cells. This suggests that BiPN-IGP48 is degraded by both a lysosomal and proteasome-dependant mechanism, distinct from IGP48 itself where the proteasome has no apparent role.

**Figure 6:**
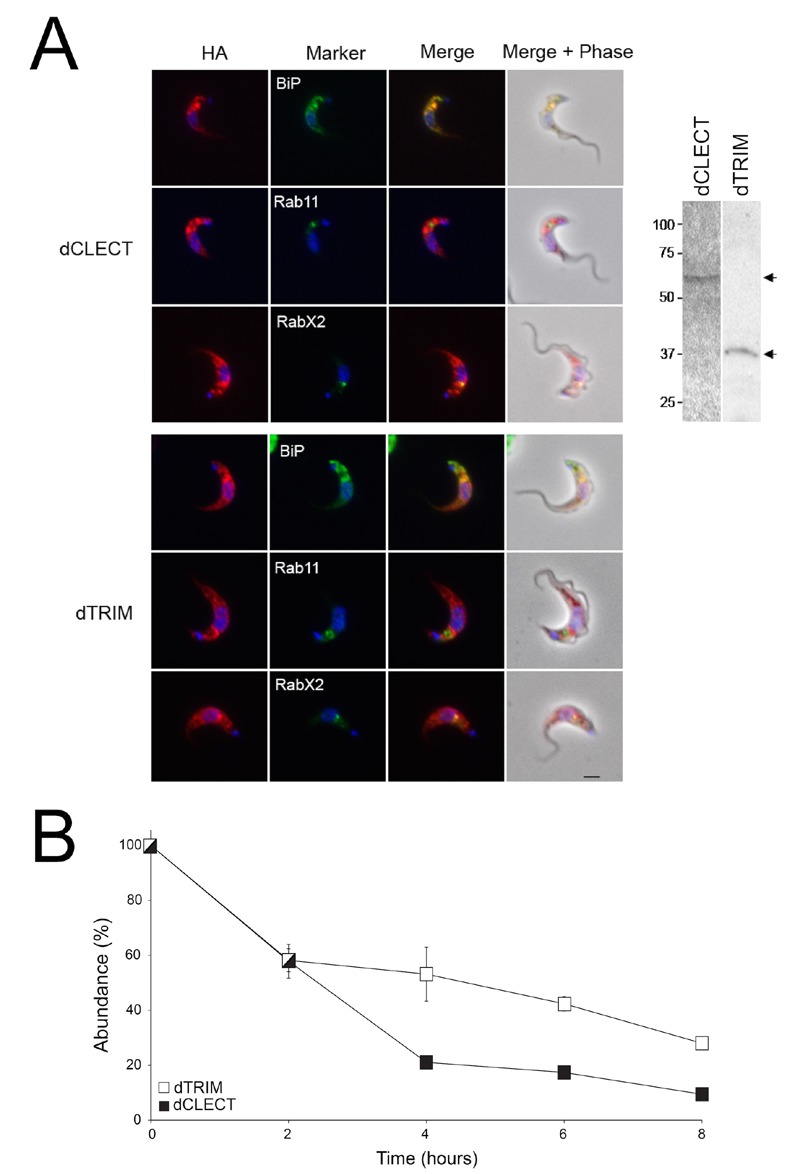
FIGURE 6: Subcellular localisation of BiPN-IGP48 chimeras is not dependent on the lumenal domain. **(A) **The IGP48 CLEC (dCLEC) or trimerisation lumenal domain (dTRIM) was replaced with the BiP ATPase domain and the location determined by immunofluorescence. In each case the BiPN chimera is in red and a marker protein visualised using polyclonal antibodies is in green. Scale bar = 1 μm. Verification of protein expression was carried out by Western blot (inset at right). Due to low expression, blots have been deliberately overexposed and relevant reactivity is indicated with arrows. **(B)** Turnover of dCLEC and dTRIM constructs. Protein degradation following cycloheximide treatment was monitored as described in Figure 2. Experiments were done in duplicate and error bars indicate standard error of the mean.

To determine the contribution the CLEC or TRIM domain makes to ER retention, the locations of dCLEC and dTRIM constructs were analysed by immunofluorescence (Fig. 6A). Both constructs co-localise with BiP and RabX2, and so appear retained in the ER with a minor presence in the Golgi complex. No co-localisation was observed with Rab11, suggesting that the proteins are unable to enter endocytic or late exocytic systems; the absence of surface staining indicates that these constructs do not reach the plasma membrane. Therefore, both the trimerisation domain and CLEC domain are independently sufficient for IGP48 ER retention, whereas removal of both releases the IGP48 *trans*-membrane and cytoplasmic domain from the ER. The observed molecular weights of both dCLEC (~65 kDa) and dTRIM (~36 kDa) (Fig. 6A, right panel) are in agreement with those predicted for these constructs, and therefore, it appears that neither construct is modified post-translationally to the same extent as IGP48 itself. Further, the half-life of both constructs was significantly reduced compared to intact IGP48, at two hours for dCLEC and one hour for dTRIM (Fig. 6B). Therefore, the absence of either the lectin or trimerisation domain likely destabilises the IGP48 protein.

### IGP48 forms higher order oligomeric complexes

**Figure 7:**
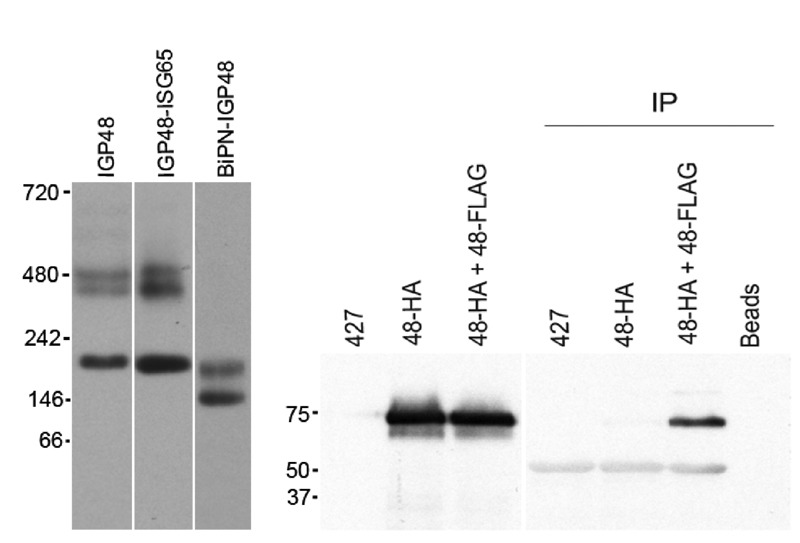
FIGURE 7: IGP48 forms a complex *in vivo*. Lysates from cells expressing various IGP48 chimeras were subjected to native PAGE, followed by detection by Western blotting. **Left:** Replacement of the IGP48 ectodomain with the BiP ATPase domain (BiPN-IGP48) results in loss of the high molecular weight (450 - 500 kDa) complexes. **Right:** Cells expressing IGP48-HA or IGP48-HA (48-HA) plus IGP48-FLAG (48-FLAG) were immunoprecipitated with anti-FLAG antibody, followed by Western blotting with anti-HA antibody. Whole cell lysates are shown to the left, and wild-type (427) and single transfected cells, as well as a bead plus lysate with no antibody IP control (Beads).

The finding that retention by the ER required the lumenal domain of IGP40 or IGP48 suggested that a simple sequence-based signal may not be responsible for targeting, and we considered that assembly into a higher order complex could contribute. To investigate this, lysates of cells expressing full-length IGP48-HA, IGP48-ISG65 or BiPN-IGP48 were analysed by blue-native PAGE and Western blotted using anti-HA antibody (Fig. 7, left panel). Each protein was detected as multiple bands, and significantly for IGP48 and IGP48-ISG65, the patterns were quite similar, with an intense band at approximately 200 kDa and a less intense doublet at ~450 kDa. This behaviour suggests incorporation into complexes, with multiple isoforms. Significantly, when the IGP48 ectodomain was replaced with BiPN, the slower migrating forms were lost and bands of only ~146 kDa and ~200 kDa forms were observed, suggesting that higher order complex formation required the IGP48 ectodomain.

To determine if these oligomeric complexes represent homomeric interactions, co-immunoprecipitations from cell lines harbouring both HA and FLAG epitope-tagged IGP constructs was performed (Fig. 7, right panel). Immunoprecipitation with anti-FLAG followed by Western blotting with anti-HA antibody revealed a band corresponding to IGP48 in the cell line containing both IGP48-HA and IGP48-FLAG, indicating that IGP48 can form homomeric interactions. Incubation of whole cell lysates of the double tag-containing cell line (IGP48-HA and IGP48-FLAG) with protein A beads but no anti-FLAG antibody shows that protein is not binding to the beads non-specifically, while no bands were detected from non-transfected cells or a cell line containing only IGP48-HA. Therefore, the IGP ectodomain is both necessary and sufficient for protein-protein interactions within the ER, for homo-oligomerisation and is required for ER retention.

### IGP48 is up-regulated in *in vitro* surrogates of bloodstream to procyclic stage differentiation

Alterations in both copy number and protein location between trypanosome life stages are well known 46,47. We asked if cell stage-specific expression level of IGP48 also led to changes in location. Laboratory-adapted monomorphic slender trypanosomes, including Lister 427 MITat1.2 strain, generated by long-term passage 48, have lost the ability to develop into short stumpy cells *in vivo*. However, these strains are able to differentiate into partially-differentiated cells in response to cold shock (ΔT > 15^o^C), where the expression of PCF surface antigens is induced as well as rendering cells 1000-fold more sensitive to *cis*-aconitate-induced differentiation 49. Differentiation can also be mimicked *in vitro* by treatment with a membrane-permeable cAMP derivative, 8-(4-chlorophenylthio)-cAMP (pCPT-cAMP) 3,50.

Whole cell lysates were generated from parasites incubated at 37°C, 20°C or with 1 mM pCPT-cAMP for twelve hours, with equivalent cell numbers used per lane (Fig. S6). Protein was separated by SDS-PAGE and analysed by Western blot using rabbit anti-IGP48 polyclonal antibody. Blots were stripped and re-probed for ISG75, to show that changes to IGP48 protein levels were specific and that there was not a global change to protein expression levels. Blots were also probed for lysosomal protein, p67, which is up-regulated in cells treated with pCPT-cAMP 51 and for PAD2 which is also under thermoregulation and serves as a cold-shock 46. Both proteins were up-regulated under conditions which promote differentiation. IGP48 protein levels increase by ~1.5-fold in cold-shock and pCPT-cAMP treated cells, showing that protein levels do increase under these conditions, albeit less dramatically than for true stumpy cells (cf. Fig. 2).

**Figure 8:**
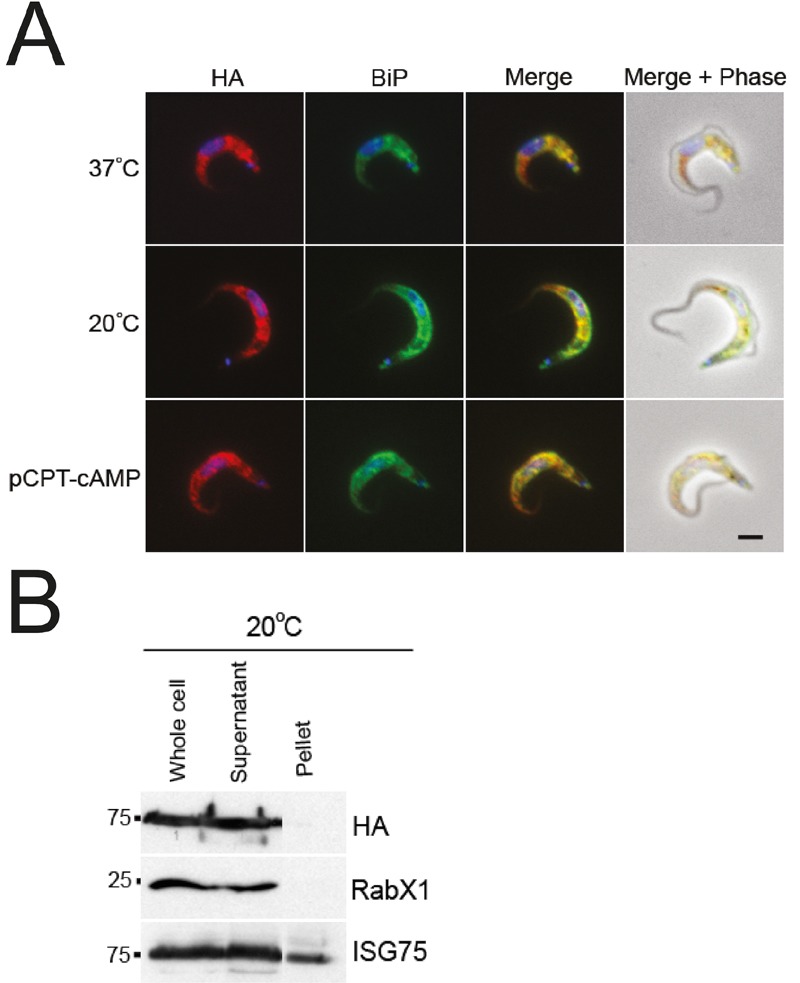
FIGURE 8: IGP48 is retained in the ER in short stumpy-like cells. **(A)** BSF cells expressing IGP48-HA were incubated at 37°C, 20°C (cold-shock) or with pCPT-cAMP for 12 hours. IGP48-HA was visualised with anti-HA antibody and co-stained with anti-BiP antibody. IGP48-HA remains in the ER in short stumpy-induced cells. DNA was visualised using DAPI. All images are captured at the same magnification, scale bar 2 μm. **(B) **Surface biotinylation was performed to determine if IGP48-HA reaches the cell surface in short stumpy-like cells. Cells were cultured *in vitro* at 37°C or 20°C for 12 hours and the biotinylation assay was carried out as described previously. IGP48-HA was detected by Western blot with anti-HA antibody. Blots were stripped and re-probed for an intracellular marker, RabX1 (localises to the ER) and a surface marker, ISG75, which localises to both the surface and endosomal compartments.

To determine whether full-length IGP48 undergoes developmentally regulated routing in cells incubated at 20°C or with 1 mM pCPT-cAMP compared to cells incubated at 37°C, immunofluorescence analysis was used (Fig. 8). Cells co-stained with anti-HA and anti-BiP antibody showed significant co-localisation, suggesting that in these differentiation models the majority of IGP48 remained in the ER. To establish if IGP48 reached the plasma membrane, surface biotinylation was carried out on cells cultured at 20°C for twelve hours (Fig. 8). IGP48-HA did not appear to reach the cell surface in these cold-shocked cells. Optical sections were taken using confocal microscopy of fixed, non-permeabilised cells expressing N-terminal HA-tagged IGP48 stained with anti-HA antibody so that only surface proteins will be stained if present (Fig. S7). No IGP48 protein was visible at the cell periphery in the central confocal stacks for cells incubated at 20°C, in agreement with biotinylation analysis. Therefore, it appears that IGP48 remains within the ER on the receipt of differentiation signals/mimics, and up-regulation of this protein was confirmed for both *in vitro *differentiation and *in vivo *stumpy cells.

### IGP48 is not required for maintaining global glycosylation status

**Figure 9:**
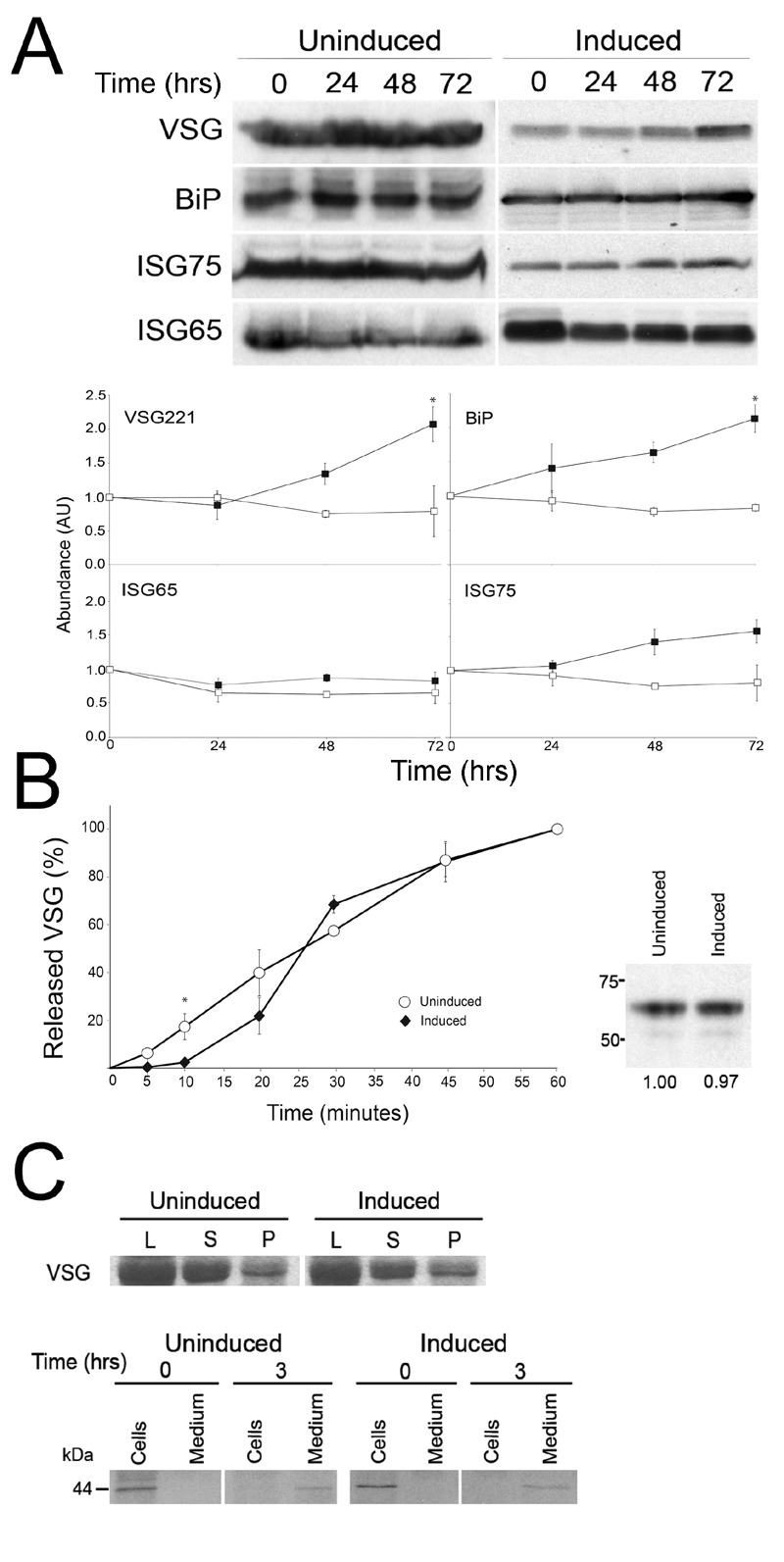
FIGURE 9: Effects of IGP knockdown on major surface protein copy number and exocytosis. **(A)** Cells were sampled from either induced or uninduced IGP48 RNAi cell lines at the times indicated. Membranes were probed with anti-VSG221, TbBiP, ISG65, or ISG75 and relative protein abundance was determined by densitometry. Experiments were done in duplicate and bars indicate standard error of the mean. Data were normalised to 100% at t = 0. **(B)** Export of newly synthesised VSG in induced and uninduced IGP48 RNAi cells. Surface accessible VSG was hydrolysed by GPI-PLC after hypotonic lysis of the cells. Soluble and membrane-form VSG was recovered by incubation with ConA-sepharose. Data represent the kinetics of newly synthesised VSG transported from the endomembrane system to the cell surface, shown as percent of VSG at the cell surface. Data were taken from two independent experiments and standard error of the mean is shown. Student’s t-test showed statistically significant difference between induced and uninduced cells at the time point indicated with an asterisk (p < 0.05). **Right panel:** Metabolic labelling of newly synthesised VSG following 24 hour RNAi induction. Newly synthesised VSG was labelled with ^35^S-methionine and detected by autoradiography. **(C)** **Top:** Hypotonic lysis, followed by separation of surface (supernatant, S) and intracellular (pellet, P) VSG 221 was visualised by SDS-PAGE and Commassie staining and levels of VSG compared to that in whole cell lysates (L). No significant difference in VSG distribution is seen between induced and uninduced cells. **Lower:** Levels of the BiPN reporter following labelling of cells with ^35^S-methionine were detected in induced and uninduced cells, following a 3 hour chase. No significant change is seen between export of BiPN from the cell in induced compared to uninduced trypanosomes.

Since both IGP40 and IGP48 reside in the ER and possess a putative lectin-like domain, we suspected a role in quality control and/or folding of newly synthesised proteins. The impact on glycosylation status was examined by separating whole cell lysates from IGP48 RNAi induced and uninduced cells by SDS-PAGE and probing Western blots with either *Erythrina cristagalli* (EC) or *Ricinus communis* (RC) lectins conjugated to biotin (Fig. S8) to recognise N-acetyllactosamine (LacNAc) or terminal β-galactose respectively. No significant differences were found, indicating that glycosylation is not generally impacted by IGP40 or IGP48 depletion. Further, Western blotting indicated that ISG75 and ISG65 copy numbers did not significantly change between induced and uninduced cell lines, although levels of VSG221 and BiP did significantly increase (p < 0.05) (Fig. 9). A similar phenotype was present in cells silenced for proteins involved in ER quality control 45.

To analyse the internal and surface presence of BiP and VSG221 in these RNAi lines, cells were analysed by immunofluorescence (Fig. S9). Confocal microscopy was used to analyse central sections of permeabilised cells stained with anti-BiP at 24 and 48 hours post-induction. After 24 hours little difference is seen between induced and uninduced cells, whereas after 48 hours of induction cells with disrupted ER structures are evident. Central optical sections of permeabilised cells stained with anti-VSG221 again showed little difference between induced and uninduced cells after 24 hours, although ~ 5% of cells induced for 48 hours appeared to accumulate VSG in the ER, suggesting that by depleting IGP48, export of protein from the ER was affected. Immunofluorescence analysis of surface VSG in non-permeabilised cells showed little significant difference between the induced and uninduced cells.

To address the contribution of IGP48 in exocytosis, the export of VSG was monitored as described previously 25. VSG export was monitored for up to 60 minutes in cells induced for IGP48 RNAi for one day (Fig. 9B, left panel). A very minor defect in VSG export to the surface was seen in induced cells at 10 minutes (p<0.05), whereas no significant difference is seen between induced and uninduced cells at later time points. Metabolic labelling indicates no apparent change to VSG synthesis levels or the distribution between internal and surface pools (Fig. 9, right panel), again similar to previous observations from silencing of ER chaperones 45. However, previous results (Fig. 9) suggest there is accumulation in total VSG221 protein levels after 72 hours of RNAi. A possible explanation for this is that VSG221 protein is being turned over less rapidly than in uninduced cells rather than increased synthesis, after prolonged IGP48 depletion. In addition, an export assay using a BiPN reporter construct showed no obvious differences between levels of exported BiPN in induced and uninduced cells after 3 hours (Fig. 9, bottom right). Overall, these data suggest that while IGP48 is located at the ER, the impact of silencing of this protein on exocytosis or the global N-glycosylation state is somewhat minimal.

### IGP48 is required to maintain normal ER morphology

**Figure 10:**
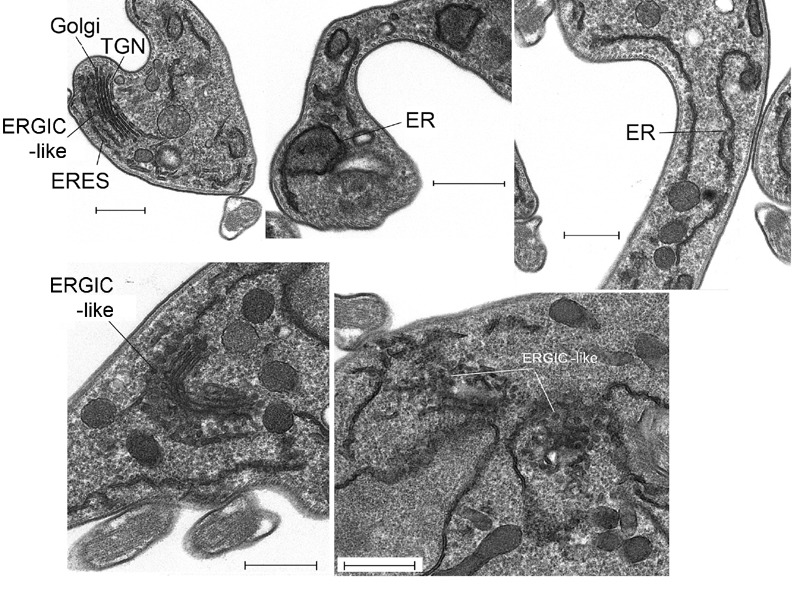
FIGURE 10: Ultrastructural analysis of IGP48 RNAi cells reveals defects in the ER.Transmission electron micrographs of IGP48 RNAi cell lines. **Top left:** Representative uninduced IGP48 RNAi cell with major secretory pathway organelles indicated. Other panels are induced cells after 24 hours induction. **Top centre:** Distorted ER with apparent lumenal inclusion. **Top right:** ER tubules with apparent normal morphology. **Lower left:** Extensive vesicles associated with the Golgi complex. Based on observations that the Golgi complex is concave towards the *trans-*face (see top left panel), these vesicles are likely ER to Golgi transport intermediates corresponding to a structure similar to the ERGIC, i.e. ER-GIC-like. **Lower right:** Examples of extensive clusters of vesicles in close association with ER tubules. Scale bars are 500 nm. **Abbreviations:** ER, endoplasmic reticulum; ERGIC, ER-Golgi intermediate compartment; TGN, *trans-*Golgi network.

We used electron microscopy to assess the impact of IGP48 knockdown on the morphology of intracellular organelles in more detail (Fig. 10). There was no evidence for ER hyperplasia or large autophagosomes, which had been observed previously following knockdown of ERAP32 and ERAP18, two ER resident proteins. However, although the Golgi apparatus in induced cells appeared normal, a large number of vesicles were present in many (approximately 25%) of the cells in which the Golgi was visible, located between the ER and cisternal face of the Golgi (Fig. 10, bottom). These vesicles may represent accumulations of structures analogous to the ER-Golgi intermediate compartment (ERGIC) in mammalian cells and/or transport vesicles and possibly reflect a defect in the transport of cargos between the ER and Golgi complex. This is also consistent with an intracellular accumulation of VSG in a subset of cells (Fig. S9), and suggests that the disruption of IGP48 expression also has an influence on the production and/or consumption of ER-derived transport vesicles.

The accumulation of what appears to be vesicles at an ERGIC-like site, positioned between ER exit sites and the *cis*-Golgi compartments and coincident with the accumulation of VSG within the ER and other subcellular compartments, and the increase to levels of the major ER chaperone BiP, suggested the possible induction of ER stress in response to loss of IGP48. ER stress can lead to an autophagy response, and to examine this issue IGP48 knockdown cell lines were transfected with YFP-ATG8.2, a marker for autophagosomes 48. Following 48 hours of induced RNAi, immunofluorescence analysis was used to determine the relative number of ATG8-positive autophagosomes per cell compared to uninduced cells (Fig. S10). There was no significant difference between the relative number of ATG8-positive autophagosomes in induced compared to uninduced cells, indicating that IGP48 depletion does not activate an ATG8-dependant autophagy pathway, indicating that a major ER stress response was not present.

### IGP48 contributes to the immune response in humans

The increased expression of IGPs in stumpy forms, which in infected hosts are lysed at high frequency, prompted us to ask if IGP proteins were detected by the immune system. We analysed sera selected from a panel assembled from infected and non-infected matched controls gathered from endemic areas of Africa. Significantly, an immune response against any member of the IGP family has not been previously reported.

**Figure 11:**
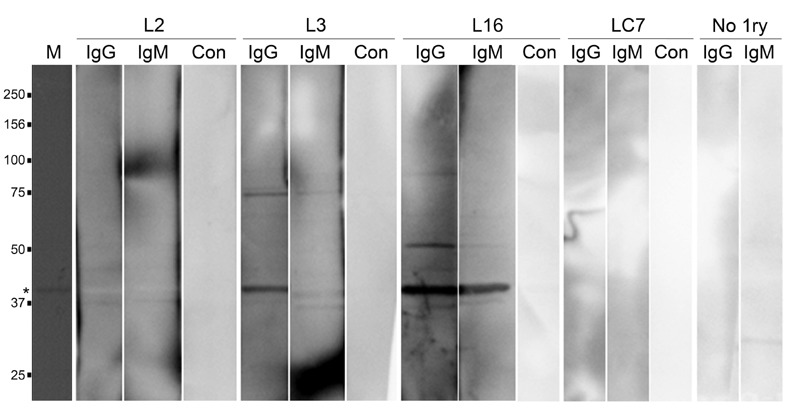
FIGURE 11: IGP48 elicits variable IgG and IgM responses in human *T. b. rhodesiense* infections. Recombinant IGP48 was resolved on a 10% SDS-PAGE gel. After Western Blotting, PVDF membranes were probed with plasma from *T. b. rhodesiense* patients and controls. **Lane M:** molecular weight markers. **Lane 1 **(from left): Ponceau red staining of IGP48 (*). **Lanes 2, 3 and 4:** membrane probed with plasma L2 with anti-IgG, anti-IgM and no secondary antibody control respectively. **Lanes 5, 6 and 7:** membrane probed with plasma L3 with anti-IgG, anti-IgM and no secondary antibody control respectively. **Lanes 8, 9 and 10: **membrane probed with plasma L16 with anti-IgG, anti-IgM and no secondary antibody control respectively. **Lanes 11, 12 and 13:** membrane probed with endemic control plasma LC7 with anti-IgG, anti-IgM and no secondary antibody control respectively. **Lanes 15 and 16**: anti-IgG and IgM controls with no primary antibody.

In the *T. b. rhodesiense* patient plasmas, three types of response were observed to bacterially expressed IGP48, namely IgG plus IgM, IgG only and no detectable response (as exemplified by L16, L3 and L12 respectively, Fig. 11). Additionally, in plasma from control individuals (endemic region, uninfected and European non-endemic/exposed), no responses were detected (Table S3). No significant relationships between patient immunoglobulin responses to rIGP48 and a range of demographic, pathobiological parameters, including stage of infection, ethnicity, gender, anaemia, plasma IgG and IgM concentrations and plasma cytokine (IFN-γ, TNF-α, IL-6 and IL-10) concentrations were detected (data not shown), and nor was there a relationship to total serum IgG and IgM concentrations. It was observed that cases showing no IgG response to rIGP48 were of significantly lower thick film parasitaemia than those who responded (median counts per 10 fields 0.125 versus 20 p<0.05 Mann-Whitney U-test). More data are clearly required to substantiate the possibility that responses to IGP are more significant in those with higher parasitaemia.

## DISCUSSION

We identified a new family of glycoproteins in African trypanosomes by searching the *T. brucei *genome for membrane proteins with a predicted type I topology. We designated this new family as invariant glycoproteins, or IGPs. Phylogenetic analysis and comparative genomics indicates that IGPs are an early feature of trypanosomatid evolution, and the family exhibits frequent and lineage-specific gene expansions, even between closely related trypanosome species. This complex evolutionary history suggests that IGPs have evolved rapidly with the differing life styles between trypanosomatids. This is supported by the importance of each specific IGP subfamily to normal proliferation. Further, the absence of IGPs from *B. saltans* suggests that IGPs could have arisen coincident with acquisition of a parasitic lifestyle, and may indicate specific roles in the evolution of close association with a host and the challenges that this provides. The surface proteomes of the *Leishmanias* (including Phytomonads), African and American trypanosomes are highly distinct and may necessitate modifications to the ER environment to accommodate these differences, and are examples of well established host adaptations, critical for the transitions between mammalian and insect vectors.

The ER quality control system as described in mammals includes calnexin/calreticulin, BiP, several protein disulphide isomerases and a group of ER-degradation-enhancing α-mannosidase (EDEM) proteins. Expression levels of many of these proteins are modulated via transcriptional responses 51, which are largely absent from trypanosomatids 52,49. However, *T. brucei*, together with other trypanosomes, does possess orthologs of hsp/dnaj chaperones, PDIs, calreticulin and EDEM pathway factors 52, while several trypanosome-specific proteins have been assigned to the ER. For example, two ER-associated proteins, ERAP18 and ERAP32, were described recently; both influence VSG surface expression and lead to ER hypertrophy 25. Therefore IGPs and ERAPs represent distinct cohorts of trypanosome-specific ER proteins. The complexity of the IGP gene family likely compromised our ability to detect major trafficking blockades, such that while the subfamilies are individually important, some redundancy may also be present in terms of functionality during knockdown, despite our use of pan-subfamily constructs.

While the presence of the lectin domain suggests a role in glycosylation or glycan recognition, we were unable to detect a major defect in the N-glycans for IGP48 knockdown, as probed by a small panel of lectins. While this finding indicates that IGP48 subfamily members are not major effectors of N-glycan levels, it is possible that only a subset of specific glycoproteins are affected, as described for mammalian ERGIC-53 53, or that an unrelated function for IGP48 within the ER is important.

Significantly, the structure of the IGPs and the oligomerisation behaviour of IGP48 suggests that these proteins have at least architectural similarities to ERGIC-53, which itself consists of a C-type lectin domain and a stalk, followed by a TMD and cytoplasmic domain. Further, ERGIC-53 forms homohexamers and also cycles between the ER and Golgi complex, which is suggested for IGP48, based on localisation and analysis of the N-glycan structural classes. However, there is no sequence relationship between the IGPs and ERGIC-53, and if ERGIC-53 and IGP are derived from a common ancestor, we would expect them to have similar cargo interactions. As the known cargo for ERGIC-53 are specific to higher eukaryotes, this is difficult to test, but exploration of the interactions between IGPs and cargo proteins is currently being attempted by immunoaffinity purification. Again, the absence of bulk effects is one more significant similarity in function between IGPs and ERGIC-53, as ERGIC-53 mutations appear to affect only a small subset of secretory proteins, while up-regulation of ERGIC-53 following tunicamycin-mediated stress suggests responsiveness to changing conditions within the ER 54, possibly mirroring the slender to stumpy transition, as suggested here for the IGPs.

The absence of an obvious role for the cytoplasm-oriented portion of IGP40 or IGP48 was unexpected, as this region of the protein is expected to interact with coatomer protein systems. However, we mapped the region responsible for ER-retention to the N-terminal lumenal/ecto-domain. For IGP48 we could demonstrate that this portion of the protein allows homo-oligomerisation and the formation of high molecular weight complexes, which we speculate also form *in vivo*. Both the CLEC and trimerisation domains of IGP48 appear sufficient to retain the protein within the ER. As wild type proteins remain within the parasite, this may suggest that retention is due to truncated IGPs retaining an ability to associate with wild type IGP48 proteins, but it is unclear if IGPs can form hetero-complexes, either between IGP subfamilies or with unrelated proteins. We noted the presence of an extensive region of VDENTT heptad repeats in IGP48, which is potentially an oligomerisation signal. Direct characterisation of IGP oligomers is clearly required to address this issue. Removal of the IGP40 and IGP48 ectodomain released these proteins from the ER and into endosomes, the cell surface and into proteolysed secreted fragments. This failure to be specifically targeted to the plasma membrane, or to a specific intracellular compartment, suggests that additional potent targeting signals are unlikely to be present in the TMD or cytoplasmic regions of either IGP40 or 48 subfamilies, and represents a relatively nonspecific localisation, resulting from the absence of any post-ER targeting information.

The IGP family is up-regulated in the stumpy life cycle stage at the mRNA level and, at least for IGP48, the protein level. Unlike PAD2, the localisation of IGP48 is unaltered in stumpy cells and remains within the ER, making it unlikely that the IGP family are developmentally routed during differentiation, but that they remain within the ER where they may act in remodelling of the cell surface during developmental transition via altering of the ER environment. Increased expression in stumpy forms may be coupled to recognition by the human immune system. Specifically, as stumpy cells die, intracellular IGP will be released and sampled by the immune system. Immune recognition of IGP48 was confirmed using *T. b. rhodesiense* sleeping sickness patient sera from Uganda. While endemic controls showed no antibody response to IGP48, infected individuals showed a range of responses. The lack of any relationship to total IgG and IgM is an evidence that this is a specific response, rather than a result of polyclonal Ig activation 55, and is consistent with variable serum IgG responses to invariant trypanosome antigens, including ISG64, ISG65, and ISG75 36. The relationship of parasitaemia to detectable IgG responses to IGP48, while based on a very small sample, was significant, and this requires further investigation to determine its immunological impact and whether the monitoring of IGP antibody is of utility in assessing disease progression.

In conclusion, the IGP family represents an extensive, rapidly evolving family of *trans*-membrane domain proteins. Some IGP subfamily members were localised to the ER, and in African trypanosomes all three subfamilies appear to be essential for normal cell proliferation. An unusual mechanism of retention by oligomerisation is suggested by a combination of complex formation and requirement for the lumenal/ecto-domain for both aspects, and taken together there is considerable evidence that the behaviour of the IGPs is similar to ERGIC-53. We speculate that IGP oligomers may form a specialised matrix for folding and/or trafficking of VSG or of surface macromolecules during the developmental switch between VSG and procyclin, but clearly requires direct analysis of IGP interactions to move beyond speculation. Significantly, increased expression in short stumpy forms suggests a specific role in conditioning the ER during transition between hosts, and the detection of IGP48 by human sera provides evidence that antigens released from stumpy cells during necrosis can be sampled by the immune system.

## MATERIALS AND METHODS

### Informatics

A series of *in silico *filters for identification of predicted type I *trans*-membrane domain proteins were used (Fig. S1); type I topology has a single TMD with the N-terminus lumenal/extracellular and the C-terminus within the cytosol. The TREU927 predicted proteome (http://tritrypdb.org/tritrypdb) was scanned using SignalP HMM for the presence of a putative N-terminal ER-targeting signal (http://www.cbs.dtu.dk/services/SignalP-2.0) . Sequences predicted to contain signal peptides and/or signal anchors were retained. As ER-targeting signals can be confused with mitochondrial N-terminal signals, the resulting sequence cohort was scanned with Mitoprot (http://ihg.gsf.de/ihg/mitoprot.html ), Predotar (http://www.hsls.pitt.edu/obrc/index.php?page=URL1043959648) and TargetP (http://www.cbs.dtu.dk/services/TargetP) . Predicted mitochondrial proteins were removed. Using SignalP HMM predictions, mature protein sequences were generated for all retained proteins in order to negate recognition of the signal sequence as a TMD. These sequences were then entered into TMHMM (http://www.cbs.dtu.dk/services/TMHMM) to select sequences containing a single TMD. Finally, GPI-SOM (http://gpi.unibe.ch) and big-PI (http://mendel.imp.ac.at/gpi/gpi_server.html) were used to remove proteins containing a predicted C-terminal GPI-anchor signal. A neighbour joining tree was generated from the remaining sequences using ClustalW2 (Fig. S2A).

To find any further members of the IGP family across the eukaryotic lineage, the twenty *T. brucei *IGP sequences were used as BLASTp 56 queries against a panel of eukaryotic predicted proteomes. A ClustalW 57 neighbour-joining tree was generated from a ClustalW alignment of all hits and a trypanosome-specific cluster, containing the twenty IGP queries, was recovered. These cluster-derived sequences were aligned with Muscle 58. Poorly-aligned sequences in the N-terminal and the C-terminal regions were removed in Jalview 59. This alignment/domain query was then used to search a panel of eukaryotic predicted proteomes with PSI-BLAST 60 as described 61, until the search converged. A ClustalW neighbour joining tree was generated from a ClustalW alignment of all matches. IGP paralogs were found in most trypanosomatid genomes (see Fig. S2C). Poorly-aligned sequences in the N-terminal and the C-terminal regions were again removed in Jalview. The domain was submitted to pfam and subsequently classed as pfam family PF16825, domain DUF5075.

For phylogenetic reconstructions, all protein sequence alignments were generated by ClustalW2 57 and manually edited to remove poorly aligned regions. For phylogenetic reconstructions PhyML 62 and MrBayes 63 were used with default parameters. Protein domain predictions were performed at Pfam (http://pfam.xfam.org) and Superfamily (http://supfam.cs.bris.ac.uk/SUPERFAMILY) (Fig. S2B).

### Cell culture

Long slender bloodstream form (BSF) *T. brucei* were maintained as described 64. Briefly, BSF Molteno Institute trypanosomal antigen type (MITat) 1.2 cells, derived from Lister 427 and expressing VSG 221, were cultured in HMI-9 complete medium 65 at 37˚C with 5% CO_2_ in a humid atmosphere in non-adherent culture flasks with vented caps. Cells were maintained at densities between 1 x 10^5^ and 2 x 10^6 ^cells/ml. For RNAi, the single marker T7^RNAP/TETR^ BSF cells (SMB) line was used 34. Expression of integrated plasmid constructs was maintained in MITat and SMB cells using G418 antibiotic selection at 2.5 μg/ml and G418 plus hygromycin selection at 2 μg/ml, respectively. Induction of RNAi was carried out with tetracycline (Sigma) at 1.0 μg/ml. Procyclic culture form cells were grown exactly as described using SDM79 medium 34. BSF stumpy stage mRNA and protein extracts were obtained from cells propagated in mice, a kind gift from Keith Matthews (University of Edinburgh).

### *In vitro* short stumpy differentiation/cold shock

Cells were incubated with 1 mM 8-(4-chlorophenylthio)-cAMP (pCPT-cAMP) 66, or incubated at 20˚C 47 for 12 hours to induce states with resemblance to differentiating cells in monomorphic 427 BSF cells.

### Plasmid constructs and transfections

Constructs for ectopic expression were designed so that an HA- or FLAG-epitope tag was incorporated into the gene sequence. For RNAi, silencing fragments were selected using RNAit 67. Primers used for PCR amplification are given in Table S1. PCR products were cloned into the BSF expression vector pXS519 68 or p2T7 69 vector for RNAi. All constructs were verified by standard sequencing methods (Geneservice Ltd.). YFP-ATG8.2::GL2166, an expression plasmid encoding a fusion protein between YFP and one *T. brucei* ATG8 paralog was a kind gift from Jeremy Mottram, University of Glasgow. Prior to introduction into trypanosomes, about 15 μg of pXS5 and p2T7 constructs were linearised with XhoI or NotI (NEB) respectively. Transgenic BSF lines were generated by electroporation using an Amaxa Nucleofector®II, incubated for ~6 hours and selected in the presence of the appropriate drug. Viable cells were taken from plates where less than 50% of wells contained transformants (considered likely clonal) and further expanded in the presence of antibiotic(s).

### IGP48 protein expression and antibody production

The IGP48 ectodomain sequence was amplified and cloned into the pQE30 His_6_-tag bacterial expression vector (Qiagen) as described 42. Protein expression was induced at OD_600nm_ = 0.6 by addition of 0.1 mM IPTG. Cells were incubated for 2 hours at 37˚C, pelleted and lysed in lysis buffer (1 x PBS, 100 μg/ml lysozyme, mini-complete EDTA-free protein inhibitor tablets, Sigma). Cells were crushed with a cell homogenisor (One Shot Model, Constant Systems Ltd.), Triton X-100 added to 1% and rotated at 4˚C for 30 minutes. The lysate was centrifuged at 10 000 g for 30 minutes at 4˚C to pellet cell debris. His_6_-IGP48 was affinity purified from the soluble fraction using the QIAexpressionist system (Qiagen). To further purify the recombinant protein, the bound fraction was subjected to SDS-PAGE and the resulting band corresponding to His_6_-IGP48 excised, washed with PBS and used to immunise rabbits (CovalAb, France).

To affinity purify anti-IGP48 antibodies from rabbit serum, the His_6_-IGP48 ectodomain was coupled to CNBr-sepharose-4B following the manufacturer’s instructions (GE Healthcare). Serum and beads were then applied to a Poly-Prep Chromatography Column (BioRad) and the column washed five times with PBS. Anti-IGP48 antibody was eluted with 100 mM glycine pH 2.5 and neutralised immediately with 1 M Tris pH 8.0. Eluted antibody was washed four times in PBS using Vivaspin 20 columns (Sartorius Stedim Biotechnology), resuspended in storage buffer (PBS plus 30% glycerol and 0.01% sodium azide) and stored at -20˚C.

### Quantitative RT-PCR (qRT-PCR)

1 x 10^8^ cells were harvested at 800 g for 10 minutes at 4˚C and washed in ice-cold PBS and quick-frozen in dry ice for 1 minute. RNA was purified using an RNeasy minikit (Qiagen) according to the manufacturer’s instructions. The RNA concentration was quantified using an ND-1000 spectrophotometer (Nanodrop Technologies). qRT-PCR was performed using iQ-SYBR green Supermix on a MiniOpticon real-time PCR (RT-PCR) detection system (Bio-Rad), and quantified using OPTICON3 software (Bio-Rad) 52. The following primers were used: IGP48-RTF (CTGCAGGCTGCCAGCTCTG), IGP48-RTR (TTTAATCTCCCGTACGCAGG), IGP40-RTF (CTGCATGTGACTGCTGCT), IGP40-RTR (TGAAAGGGTATACAACTGACC), IGP34-RTF (ATTGCGTCTACCGATGGAAC), IGP34-RTR (TAGACTCCTCATCTGAATGC). Data were normalised against TERT (telomerase reverse transcriptase) (TERT-RTF (GAGCGTGTGACTTCCGAAGG) and TERT-RTR (AGGAACTGTCACGGAGTTTGC).

### Western blot analysis

Protein samples were typically resolved on 10% SDS-PAGE after solubilisation in SDS sample buffer 70 and then transferred to polyvinylidene fluoride (PVDF) membranes (Millipore). Non-specific binding sites on the membrane were blocked, and Western blotting was carried out following standard procedures.

### Immunofluorescence microscopy

Immunofluorescence analysis (IFA) was as described 64. Antibodies for IFA were used at the following dilutions: mouse anti-HA-epitope IgG (Santa Cruz Biotechnology Inc.) at 1:1000, rabbit anti-Rab11 at 1:200 44, rabbit anti-Rab5a at 1:200 68, mouse anti-p67 at 1:1000 43, mouse anti-BiP at 1:10 000 41, rabbit anti-VSG221 at 1:1000 68, rabbit anti-RabX2 at 1:50 71, rabbit anti-IGP48 (this study) at 1:50. Secondary antibodies were used at the following dilutions: anti-mouse Oregon Green (Molecular Probes) at 1:1000 and anti-rabbit Cy3 (Sigma) at 1:1000. Cells were examined on a Nikon Eclipse E600 epifluorescence microscope fitted with optically matched filter blocks and a Hamamatsu ORCA charge-coupled-device camera. Digital images were captured using Metamorph software (Universal Imaging Corp.) on a computer running the Windows XP operating system (Microsoft Inc.) and the raw images processed using Photoshop CS6 software (Adobe Systems Inc.). Confocal z-sections were acquired using a Leica DMIRE2 microscope and deconvolved using Huygens Professional software.

### N-glycosylation analysis

Bloodstream form trypanosomes were cultured in the presence of tunicamycin (Sigma) added to complete media at 1 µg/ml, which prevents further cell proliferation 52. For treatment with PNGase F, 1 x 10^8^ cells were washed in PBS and incubated with lysis buffer (1% NP-40, 100 mM NaPO_4_, pH 7.5) plus protease inhibitor cocktail (Roche) and then heated to 95˚C for 15 minutes. Samples were treated with 10 mU PNGase F (NEB) overnight at 37˚C. A second aliquot of PNGase F was added and the reaction continued for 2 hours prior to analysis. For treatment with Endoglycosidase H, 1 x 10^8^ cells were washed and lysed by incubation with 1 ml of 5 mM EDTA and protease inhibitor cocktail (Roche), followed by two cycles of freeze-thawing. Samples were centrifuged to separate crude membranes and the pellet dissolved in 10 mM Tris-HCl, pH 8.0, 1 mM EDTA, 1% SDS. 5 mU/ml of Endoglycosidase H (Calbiochem) was added to each sample, and then 5 mU/ml again 12 hours prior to analysis.

### Protein turnover

Protein synthesis was blocked by the addition of cycloheximide (35 µM) and IGP copy number estimated by Western blotting 64. Antibodies were used at the following dilutions: mouse anti-HA at 1:8000, mouse anti-β-tubulin at 1:20 000, rabbit anti-BiP at 1:10 000 and horseradish peroxidase-conjugated anti-(rabbit IgG) and anti-(mouse IgG) (Sigma) at 1:10 000. Antigen copy number was estimated by densitometry using ImageJ on scanned films derived from low exposure blots (National Institutes of Health).

### Biotinylation 

Mid-log phase cells (1 × 10^7^) were collected and washed three times in vPBS. Biotinylation and separation of biotinylated and non-biotinylated proteins were all carried out as described 14. Samples were incubated in SDS sample buffer at 95˚C, and both fractions subjected to SDS-PAGE and Western blotting. Antibodies were used at the following dilutions: mouse anti-HA at 1:8,000, rabbit anti-RabX1 (1:1 000) or rabbit anti-ISG75 (1:8 000) and blot signals quantified using ImageJ software.

### Radioimmunoprecipitation

1 x 10^7^ cells were washed in PBS and resuspended in 500 μl of Met/Cys-free RPMI 1640 (Sigma) medium supplemented with 10% dialyzed fetal bovine serum (FBS), followed by incubation at 37˚C for 1 hour. Cells were pulse-labeled for 1 hour with EasyTag EXPRESS^35^S protein labeling mix (Perkin Elmer) at a specific activity of 200 μCi/ml and chased by addition of HMI-9 medium for 3 hours. Cells were washed in ice-cold PBS, lysed in 100 μl RIPA buffer (25 mM Tris-Cl, pH 7.5, 150 mM NaCl, 1% NP40, 0.5% sodium deoxycholate, 0.1% SDS) for 15 minutes on ice, and incubated for 5 minutes at 95˚C in RIPA buffer containing 1% SDS. Lysates were pre-cleared for 1 hour with Pansorbin (Calbiochem) and incubated overnight with mouse anti-HA (1:100) at 4˚C. Immunocomplexes were isolated by incubation with protein A-sepharose (Sigma) for 1 hour and subjected to SDS-PAGE. Gels were dried on Whatman 3MM paper and exposed to autoradiographic films for up to 1 week.

### Immunoprecipitation

Cells were grown to logarithmic phase and harvested by centrifugation (800 g, 10 minutes, 4˚C) and washed twice in PBS. Cells were lysed in 500 μl of NP40 buffer (1% NP40, 150 mMNaCl, 50 mM Tris HCl pH 7.5, 1 mM EDTA, protease inhibitor cocktail) and incubated on ice for 10 minutes. Lysates were centrifuged at 16 000 g for 15 minutes at 4˚C to remove nuclei and cell debris, and the supernatant transferred to a fresh tube. Five microliters of rat anti-HA or mouse anti-FLAG was added to each sample, and the mixture incubated overnight at 4˚C. Immune complexes were isolated by addition of 50 μl of protein A-sepharose (Generon) or Dynabeads®Protein G (Invitrogen) and incubated at 4˚C with rolling for 2 hours. Beads were recovered by centrifugation at 3000 g for 5 minutes (protein A-sepharose) or by magnetisation (Dynabeads®) and washed extensively with NP40 buffer. The supernatant was discarded and bound proteins eluted by addition of 1 x SDS sample buffer and incubation at 95˚C for 10 minutes. Samples were subjected to protein electrophoresis and Western blotting for detection. Antibodies were used at 1:8000 rat anti-HA and 1:800 mouse anti-FLAG.

### Ultrastructural analysis

Tetracycline-induced and non-induced RNAi cell lines were grown to a density of 1 × 10^6^ cells/ml and rapidly fixed in culture by the addition of isothermal glutaraldehyde to the culture flask, to a final concentration of 2.5% (w/v), as described previously 72. The culture flask was rocked gently for 10 minutes at 37˚C, followed by centrifugation at 800 g for 5 minutes and harvested cells resuspended in 2.5% glutaraldehyde in PBS for another 30 minutes at room temperature. Fixed cells were post-fixed and embedded as described previously 34. Ultra-thin (70 nm) sections were viewed on a Philips CM100 electron microscope (FEI-Philips).

### VSG and BiPN transport

VSG export was monitoredas described 64 with a few modifications. Parasites were labeled with EasyTag EXPRESS ^35^S protein labeling mix (PerkinElmer) at a specific activity of 200 μCi/ml and incubated for 7 minutes at 37˚C. Following separation of membrane-form and soluble VSG by hypotonic lysis and binding to ConA, fractions were washed and resuspended in sample buffer and loaded onto 10% SDS-polyacrylamide gels at 1 × 10^6^ cell equivalents per lane. Gels were fixed, stained and exposed to X-ray film. VSG band intensity was quantified using ImageJ. BiPN export was monitored as described previously 73. Transformed IGP48 RNAi BSF cells expressing BiPHA9 were pulse-labeled for 1 hour at 37°C and then chased for 1 hour, taking samples at regular time points. Aliquots were separated into cells and medium by centrifugation, and labeled polypeptides in both fractions immuno-precipitated with anti-HA and analysed as for VSG export.

### Trypanosomiasis plasma samples and Western blotting

Plasma was prepared from heparinised blood samples from patients diagnosed with *T. b. rhodesiense* infection and non-infected local controls in Tororo, Uganda in 2003. Details of the recruitment and diagnostic methods for these samples have been described previously 54 as well as measurement of serum IgG and IgM concentrations 74. Sample collection and subsequent analyses were approved by ethical review by the Uganda Ministry of Health and the Grampian Research Ethics Committee, and were subject to informed consent. All plasma was stored at -80˚C after collection. For immunoprobing, PVDF membranes were wetted in 50% methanol (v/v), washed with PBS-T (PBS, 0.05% Tween-20) and blocked for 1 hour in PBS-T plus 5% BSA (w/v). After washing in PBS-T, filters were probed with human plasma samples diluted 1:1000 in PBS-T, 5% BSA for 3 hours with gentle rocking. Filters were then washed four times with PBS-T and probed with either no secondary antibody, peroxidase-conjugated goat anti-human IgG (Invitrogen 62-7520) or peroxidase-conjugated goat anti-human IgM (Invitrogen 62-7120) diluted 1:1000 in PBS, 5% BSA. After 1 hour incubation, filters were washed five times in PBS-T. Filters were flooded with a solution comprising equal volumes of freshly mixed 0.02% H_2_O_2 _in 20 mM Tris-HCl pH 8.5 and 2.5 mM Luminol, 0.4 mM p-Coumaric acid in 20 mM Tris-HCl, pH 8.5. After excess ECL reagent was removed, luminescence was visualised using a Fusion SL imaging system (Peqlab).

## SUPPLEMENTAL MATERIAL

Click here for supplemental data file.

All supplemental data for this article are also available online at http://microbialcell.com/researcharticles/an-extensive-endoplasmic-reticulum-localised-glycoprotein-family-in-trypanosomatids/.

## DOWNLOAD FIGURES IN ORIGINAL RESOLUTION

Win-RAR-archive, 26 Mb:

http://microbialcell.com/wordpress/wp-content/uploads/2014/09/2014A-Allison-Figures.rar
