# Supplementary material for: An extensive endoplasmic reticulum-localised glycoprotein family in trypanosomatids
Source: Microb Cell. 2014 Oct 1;1(10):325–45. doi: 10.15698/mic2014.10.170 (PMC4497807; doi:10.15698/mic2014.10.170)
Supplement: Supplementary file 1 [file mic-01-325-s01.pdf]

Supplementary material for:

An ER-localised, extensive, essential and immunogenic glycoprotein family in trypanosomes

Harriet Allison[1], Amanda J. O'Reilly[1], Jeremy Sternberg[2] and Mark C. Field1[*]

[1]Division of Biological Chemistry and Drug Discovery, University of Dundee, Dundee, Scotland, DD1 5EH and [2]School of Biological Sciences, University of Aberdeen, Aberdeen, AB24 2TZ, UK.

Corresponding author: Telephone: +44 (0)751-550-7880, email: mcf34@cam.ac.uk

Figure legends

Figure S1: Bioinformatic filters for identification of predicted type I *trans*-membrane domain surface membrane proteins. Firstly, the TREU927 proteome was downloaded from TriTrypDB (http://tritrypdb.org/tritrypdb/). All protein sequences were entered into SignalP HMM (http://www.cbs.dtu.dk/services/SignalP-2.0/) and those protein sequences containing predicted signal peptides and signal anchors were retained, whilst all others were discarded. Mitprot (http://ihg.gsf.de/ihg/mitoprot.html), Predotar (http://www.hsls.pitt.edu/obrc/index.php?page=URL1043959648) and TargetP (http://www.cbs.dtu.dk/services/TargetP/) were then used to remove predicted mitochondrial proteins. Using the output from SignalP HMM, signal sequences were removed and thus the mature protein sequence was generated for all retained protein sequences. These sequences were then entered into TMHMM (http://www.cbs.dtu.dk/services/TMHMM/). Finally, GPI-SOM (http://gpi.unibe.ch) and big-PI (http://mendel.imp.ac.at/gpi/gpi_server.html) were both used to remove all predicted GPI-anchored proteins. Only one GPI-anchored protein was found in this final output; Tb09.v4.0204. The final cohort of 208 protein IDs was parsed through the publicly available web-based mitochondrial proteome database (www.TrypsProteome.org), to check for the absence of mitochondrial-targeted proteins. Accession numbers and details for these sequences are available in Table S2.

Figure S2: Phylogenetic identification of the IGP family. (A) A neighbour joining tree was generated using ClustalW2. Adenylate cyclases are highlighted in pink, ISG and ISG-like proteins are highlighted in blue and light blue respectively. Those proteins which do not form clusters are highlighted in yellow and referred to as singletons. A family of 20 proteins, invariant glycoproteins (IGPs) are highlighted in green. (B) Phylogenetic analysis of IGPs and ISGs, which demonstrates the distinct nature of these two gene families. The IGP family is further divided into three subfamilies, designated IGP34 (two genes), IGP40 (ten genes) and IGP48 (eight genes), based on molecular weight, presence in all Salivarian trypanosomes and *T. cruzi* and also on possession of N-glycosylation sites.

Sequences from the nearest set of genes from the closer analysis in panel A were used as an out group. (C) Phylogenetic analysis of all retrieved IGP orthologs from a range of kinetoplastid genomes (see methods). Leishmania and related species are colour-coded in shades of green, African trypanosomes in shades of red/ochre and south American trypanosomes in shades of blue.

Figure S3: ClustalW alignment of IGP homologues. Panel A: Single representatives of the IGP34, 40 and 48 families are shown to illustrate the homology within the N-terminal region. Panel B: Eighty IGP family sequences, from *T. brucei brucei*, *T. brucei gambiensie*, *T. vivax, T. congolense* and *T. cruzi* were aligned using ClustalW. The alignment was used for building the phylogeny shown in Figure S2C.

Figure S4: IGP48 is a tomato lectin-binding protein. 1 x 10^7 BSF cells over-expressing IGP48 full-length protein, IGP48 ectodomain, dTRIM or wild type cells (negative control) were lysed and HA-tagged proteins immunoprecipitated with anti-HA antibody and Dynabeads® Protein G (Life technologies). This was carried out under both native and denaturing conditions (addition of detergent and heating to 95°C). Samples were separated by SDS-PAGE followed by Western blot with TL-biotin conjugate (1:10,000, Vector Laboratories) and streptavidin-HRP (1:10,000, Sigma Aldrich). Membranes were stripped and re-probed with mouse anti-HA monoclonal antibody. Antibody light (~25kDa) and heavy chains (~50kDa) can be seen for all blots. Bands corresponding to HA-tagged proteins are also seen at the correct molecular weight when probed with mouse anti-HA to verify the immunoprecipitation of HA-tagged proteins. To confirm the specificity of immunoprecipitation, wild type BSF cells were lysed and incubated with mouse anti-HA and then Dynabeads under native and denaturing conditions. Following SDS-PAGE, membrane was probed with rabbit polyclonal antibodies against the highly N-glycosylated VSG221. A protein band corresponding to VSG221 was present in the whole cell sample, and no band was observed in the native or denaturing immunoprecipitated samples, indicating that only HA-tagged constructs were isolated in the immunoprecipitation process. The addition of chitin hydrolysate was used to check the carbohydrate specificity of tomato lectin for proteins containing poly-LacNAc units.

Figure S5: Effects of IGP RNAi on cell cycle progression. Uninduced and cells that have been induced for 24 hrs were fixed and stained with DAPI. Representative images were taken for cells with normal nuclei and kinetoplast content (1K:1N, 2K:1N, 2K:2N), as well as for cells with abnormal DNA content (0K:1N, 1K:0N, 1K:2N, 0K:2N, K:N n>2). The percentage of cells with this type of DNA content out of 200 cells is indicated for induced and uninduced cells beside each image. All images were captured at the same magnification and scale bar represents 2μm.

Figure S6: Induction of IGP48 expression in stumpy and stumpy-like cells. BSF cells ectopically over-expressing HA epitope tagged IGP48 at the C-terminus were incubated at 37°C, 20°C (cold-shock), or in the presence of pCPT-cAMP for 12 hours. A Western blot of

endogenous IGP48 expression levels using rabbit anti-IGP48 antibody (1:100) is shown. Blots were stripped and re-probed with anti-ISG75 whose levels remained unchanged. IGP48 protein levels were determined by densitometry using ImageJ and quantified by normalisation to ISG75. Blots were probed for p67, which is up-regulated in cells treated with pCPT-cAMP and PAD2 (protein associated with differentiation 2), which is under thermoregulated control.

Figure S7: Visualisation of IGP48 by confocal microscopy. Cells were fixed and membranes remained intact (not permeabilised), so that incubation with anti-HA antibody stained only HA epitope-tagged proteins on the surface of the cell, if present. A representative image is shown for each condition of a single optical z-section. Confocal z sections were acquired using an SP2-visible inverted confocal microscope (Leica Microsystems GmbH, Germany). Scale bar is 2μm.

Figure S8: Analysis of glycosylation defects on IGP knockdown. (A) Whole cell lysates were prepared from p2T7 IGP48 RNAi cell lines cultured in the presence (induced) or absence (uninduced) of tetracycline for 24 hrs and proteins separated by NuPAGE® Bis-Tris Gel System (Invitrogen) using 4-12% gradient acrylamide gels, allowing high resolution separation of proteins. Analysis of protein abundance and molecular weight was carried out using ImageJ, and a line profile plot of grey value (the sum of the grey values of all the pixels in the selection) against distance from top to bottom of the blot was generated. (B) Samples were separated by SDS-PAGE and lectin blots were carried out with *Erythrina cristigalli* (EC; 1:1000) or *Ricinus communis* (RC; 1:1000) lectins conjugated to biotin and then incubated with streptavidin-HRP. A line profile plot, using ImageJ, was generated for each blot. Protein gels were also stained with Coomassie blue stain, to show equal protein loading. Each experiment was performed in duplicate with a representative experiment shown here.

Figure S9: Analysis of intracellular compartment morphology in IGP48 knockdown cells. (Left) Visualisation of ER structure and morphology of cells in *T. brucei* stained with TbBiP. (Centre and right) Distribution of VSG221 in permeabilised cells (intracellular VSG, centre) and non-permeabilised cells (surface VSG, right). Intracellular staining with BiP and VSG was visualised with confocal microscopy. Numbers on the panels indicate the period after induction when cells were taken for analysis. Scale bar, 2μm.

Figure S10: IGP48 knockdown does not induce ATG8-dependant autophagy. BSF IGP48 RNAi cells transfected with YFP-ATG8.2::GL2166. Cells were induced with tetracycline for 48 hours and fixed, permeabilised and stained with anti-GFP. The number of ATG8-positive puncta were counted for induced and uninduced cells (n = 20) and plotted as a bar graph. Error bars denote standard error of the mean. Knockdown of IGP48 is verified by Western blot, using anti-IGP48 antibody and anti-tubulin as a loading control. A Western blot probed with anti-GFP to verify molecular weight of YFP-ATG8 as 41kDa.

Table S1: Primer sequences for the verification of RNAi knockdown and localisation studies. The epitope tag is underlined and restriction sites are shown in bold. Primers are shown in 5' to 3' direction. Designation of primers is shown to the left and restriction sites and epitope tags used are given on the right.

Table S2: Accession numbers of predicted type I proteins in *T. brucei*. The accession numbers of all 208 proteins resulting from the bioinformatics search outlined in Fig. S1 are shown in the table.

Table S3: Serum immunoglobulin responses to IGP48 in *T. b. rhodesiense* patients. Immunoglobulin G and M responses to recombinant IGP48 detected by Western blotting are recorded as either positive (+) or negative (-). For each patient, disease stage, age and thick film parasitaemia (i.e. Number of Giemsa stained parasites observed per 10 fields, at 400x magnification) are recorded.

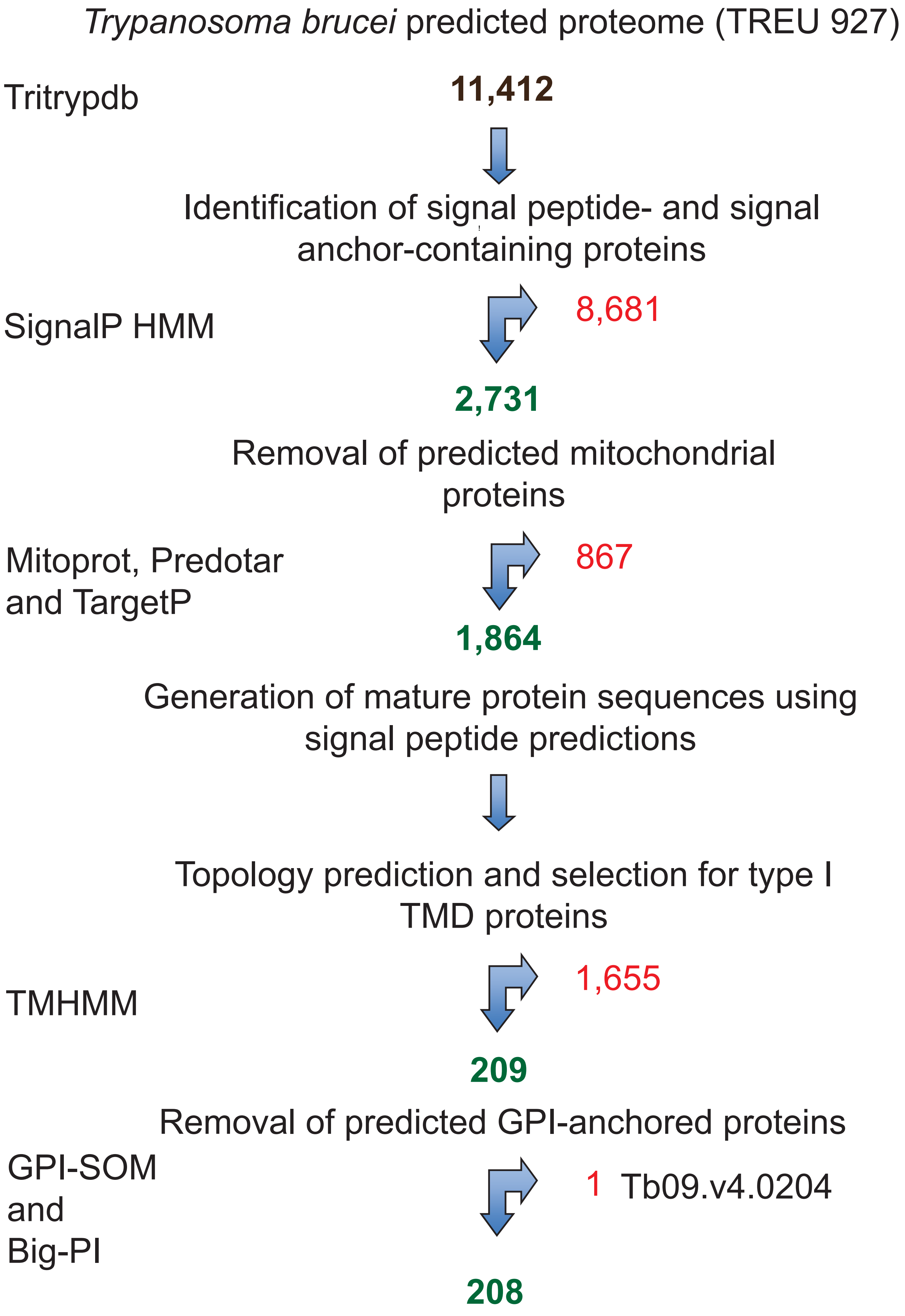
Trypanosoma brucei predicted proteome (TREU 927)
Tritrypdb
11,412
Identification of signal peptide- and signal anchor-containing proteins
SignalP HMM
8,681
2,731
Removal of predicted mitochondrial proteins
Mitoprot, Predotar and TargetP
867
1,864
Generation of mature protein sequences using signal peptide predictions
Topology prediction and selection for type I TMD proteins
TMHMM
1,655
209
Removal of predicted GPI-anchored proteins
GPI-SOM and Big-PI
1 Tb09.v4.0204
208

Figure S1

Figure S2A

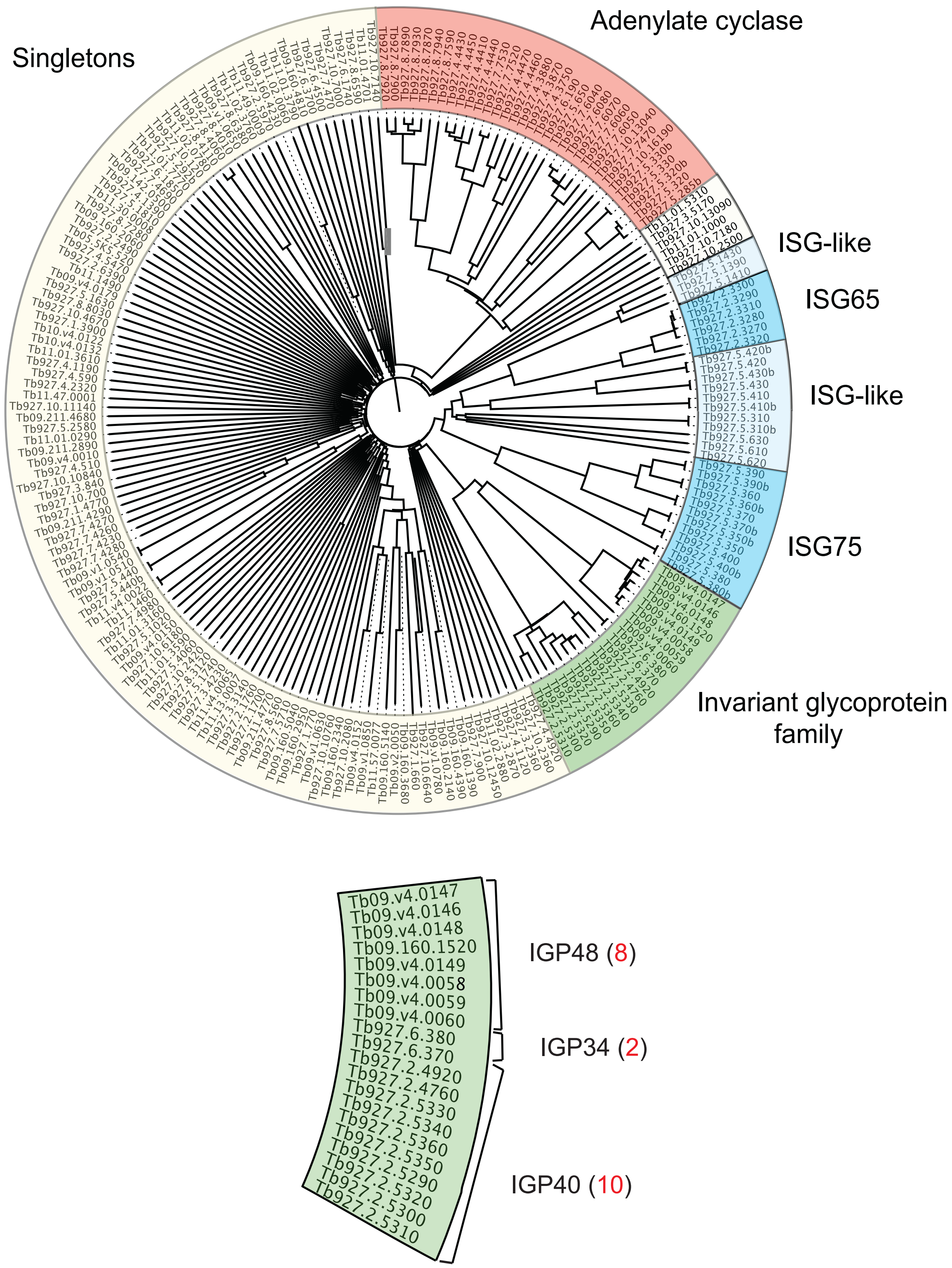

Singletons
Adenylate cyclase
ISG-like
ISG65
ISG-like
ISG75
Invariant glycoprotein family
Tb09.v4.0147
Tb09.v4.0146
Tb09.v4.0148
Tb09.160.1520
Tb09.v4.0149
Tb09.v4.0058
Tb09.v4.0059
Tb09.v4.0060
Tb927.6.380
Tb927.6.370
Tb927.2.4920
Tb927.2.4760
Tb927.2.5330
Tb927.2.5340
Tb927.2.5360
Tb927.2.5350
Tb927.2.5290
Tb927.2.5320
Tb927.2.5300
Tb927.2.5310
IGP48 (8)
IGP34 (2)
IGP40 (10)

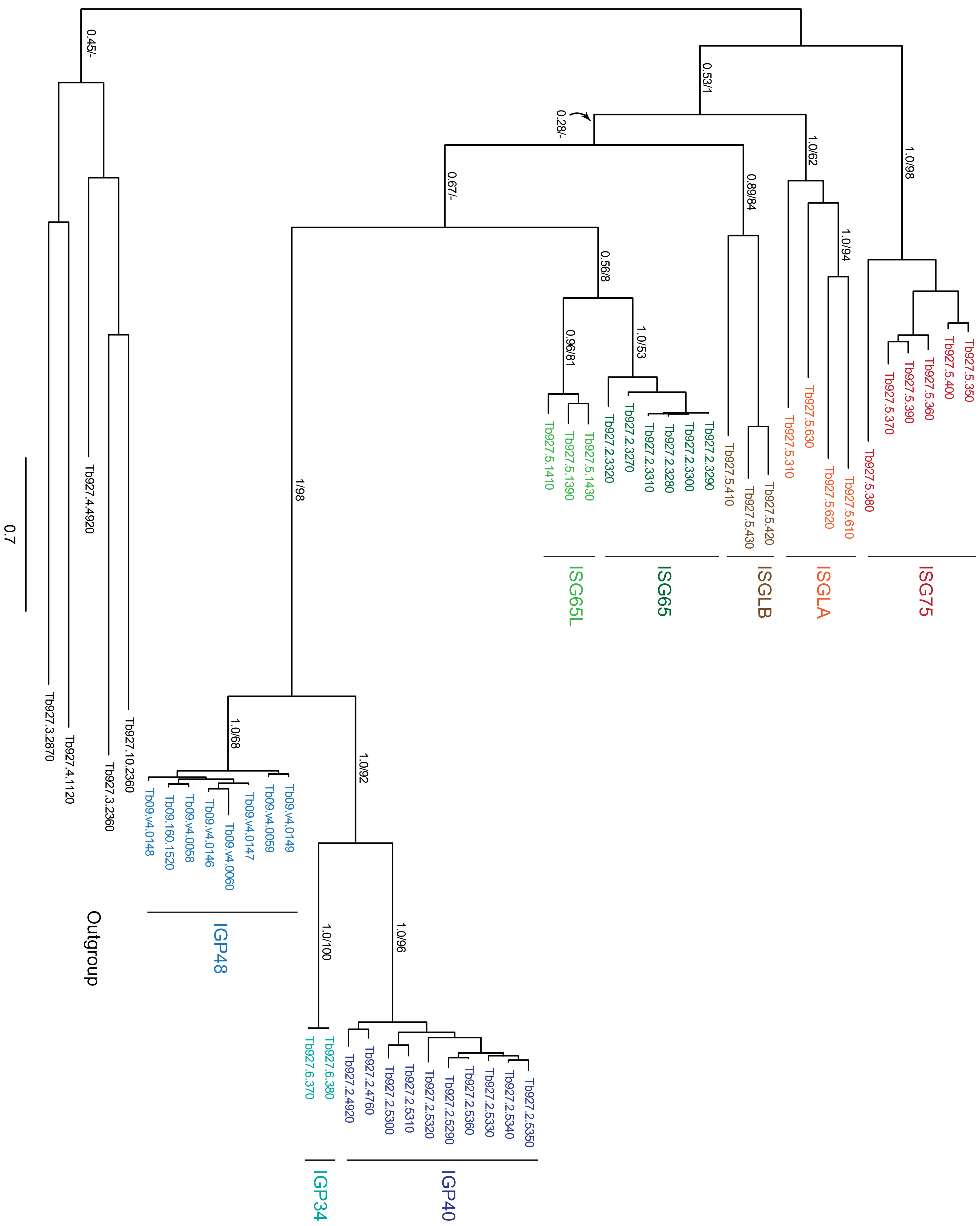
0.45/-
0.53/1
0.28/-
0.67/-
0.89/84
1.0/62
1.0/98
1.0/94
0.56/8
0.96/81
1.0/53
1/98
1.0/68
1.0/92
1.0/100
1.0/96
0.7
Tb927.5.350
Tb927.5.400
Tb927.5.360
Tb927.5.390
Tb927.5.370
Tb927.5.380
Tb927.5.610
Tb927.5.620
Tb927.5.630
Tb927.5.310
Tb927.5.420
Tb927.5.430
Tb927.5.410
Tb927.2.3290
Tb927.2.3300
Tb927.2.3280
Tb927.2.3310
Tb927.2.3270
Tb927.2.3320
Tb927.5.1430
Tb927.5.1390
Tb927.5.1410
ISG75
ISGLA
ISGLB
ISG65
ISG65L
Tb927.4.4920
Tb927.10.2360
Tb927.3.2360
Tb927.4.1120
Tb927.3.2870
Outgroup
Tb09.v4.0149
Tb09.v4.0059
Tb09.v4.0147
Tb09.v4.0060
Tb09.v4.0146
Tb09.v4.0058
Tb09.160.1520
Tb09.v4.0148
IGP48
Tb927.6.380
Tb927.6.370
IGP34
Tb927.2.4920
Tb927.2.4760
Tb927.2.5300
Tb927.2.5310
Tb927.2.5320
Tb927.2.5290
Tb927.2.5360
Tb927.2.5330
Tb927.2.5340
Tb927.2.5350
IGP40

Figure S2B

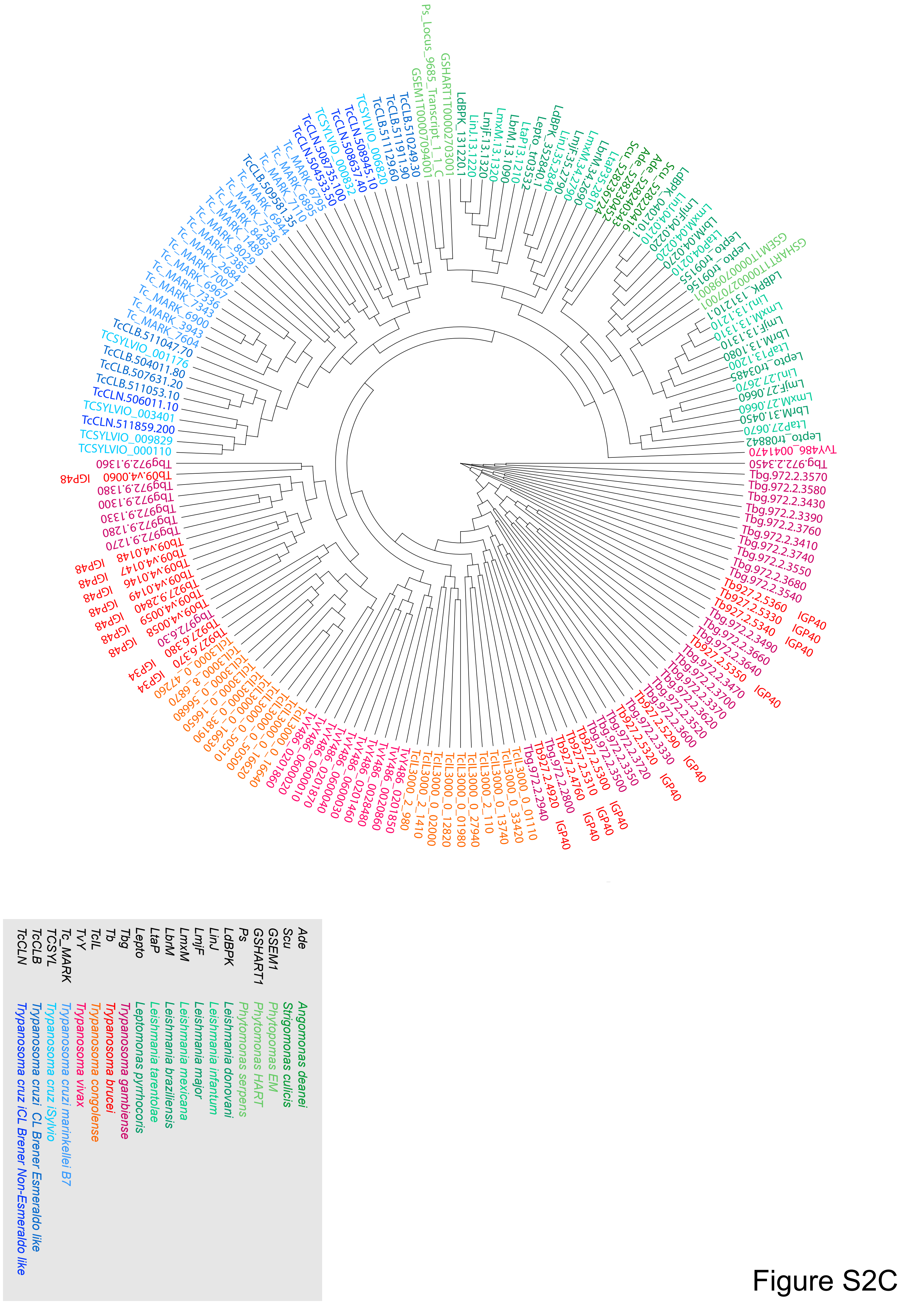
Ade Angomonas deanei
Scu Strigomonas culicis
GSEM1 Phytopomas EM
GSHART1 Phytomonas HART
Ps Phytomonas serpens
LdBPK Leishmania donovani
LinJ Leishmania infantum
LmjF Leishmania major
LmxM Leishmania mexicana
LbrM Leishmania braziliensis
LtaP Leishmania tarentolae
Lepto Leptomonas pyrrhocoris
Tbg Trypanosoma gambiense
Tb Trypanosoma brucei
TcIL Trypanosoma congolense
TvY Trypanosoma vivax
Tc_MARK Trypanosoma cruzi marinkellei B7
TCSYL Trypanosoma cruzi iSylvio
TcCLB Trypanosoma cruzi CL Brener Esmeraldo like
TcCLN Trypanosoma cruzi iCL Brener Non-Esmeraldo like

Figure S2C

Figure S3A


```
CLUSTAL 2.1 multiple sequence alignment


Tb09.v4.0147      --MEIRMVPSNFSRITTLCGILMLLQCILRPLQAASSGGGPVTTAAKSYAIYTHEVFENN
Tb927.6.380       MGTEISMTACGFARMVVLF--TALLQCLSGSSHLFAFGKVYPTAYVKEYILFPNRKFWHG
Tb927.2.5290      MSMDTRMVNFGFTRAALLCG-LLLLLTAVCPLHVTAADNGRVIVNVKSYFSMDNFAFAHR
                     :  *.  .*:* . *     **     . :  : .     . .*.*    :  * :

Tb09.v4.0147      GVYNVDYVNSERVCRAEGMNLATDHSEATNSLIYKLLKPKNKLGYLYAYLGGDAKYSASS
Tb927.6.380       GDYLVDYSNSVHICREAGGTLATDHSQATNERLTAYHLGVADGPFIYSYLGGDATYSASA
Tb927.2.5290      GNYFVSYKNAAAYCGDNGAVLPADQTEAAHQSLQSAIKRVITGSDAFSYLGGDAVYSSGE
                  * * *.* *:   *   *  *.:*:::*::. :             ::****** **:.

Tb09.v4.0147      VHEEKDRCKVGDLASSLNCVYRWNTGLFAPATPDDNGVAFWRGSYYEVTGAGSMNDYPSF
Tb927.6.380       AQDAGIRCTAGDYDTSLNCVYRWNSGLFEASGIEGNGVAFWRGSYTYKSGSGALGGYPSF
Tb927.2.5290      AIEADKRCKPGDAASSRYCVYRWNKGLFAKALPDGQGVAFWRGSYVKYGGAASMNGYPSF
                  . :   **. **  :*  ******.***  :  :.:*********    *:.::..****

Tb09.v4.0147      FEN-YPAYGRLNVIAKLD-SSDRVTWFDDDDDFGNNISFTNTRARSTRFFMVLCEASAVP
Tb927.6.380       FAQSYPQDGQLHVMGWYTNVTKWFGWFDGGEVSGNKPGGKAPGYKSLTYFAALCEVT-VP
Tb927.2.5290      WGSEYPKRGQLYTLSWYHTGDDVTTWYDGKDMSGRIKWASDNPDTKTENYVILCEVHDSI
                  : . **  *:* .:.      .   *:*. :  *.    .     .   :  ***.

Tb09.v4.0147      TPLPPASPHSENTTADENTTVDENTTVDENTTADENTTADENTTVDENTTADENTTADEN
Tb927.6.380       QPSHPVPGNGNSTANEELAVINESLHSDE-------------------------------
Tb927.2.5290      STTTTTTTTTTTTTTTTTTAAPASGEVPEP------------------------------
                   .  ...    .*:    :.   .    *

Tb09.v4.0147      TTADENTTVDENTTADENTTVDENTTVDENTTVDENTNADEISNGSNEASDKTVPSTASD
Tb927.6.380       ----------ESTNSQDQPSATPKPKGEAETTLTSLSHITICVGG---------------
Tb927.2.5290      -----------TVTKDEEVTVT-------TVSLEERSNWHIILIA---------------
                             ... ::: :.         .:: . ::      .

Tb09.v4.0147      GEGSGGGAGAAVVIIFILLAVLLILLYFCCFAGHEKYITVMSLREKVTSPVSNVEAAEVA
Tb927.6.380       --------ELCLLIVFFSLAIVC-------------------------------------
Tb927.2.5290      -----------LISVVSLIVLLLFLLRWFCDYDVKKEIIPMTLREVVGEPLFVLEPTPFV
                             :: :.  :.::

Tb09.v4.0147      AVPSNGEEHLSITSHQQETPAADE
Tb927.6.380       ------------------------
Tb927.2.5290      PPEGFAAPPLALQYYNQMRTSE--
```

Figure S3B

CLUSTAL W (1.8) multiple sequence alignment

```
Tbg97291330              ------------------------------------------------------------
Tbg97291280              ------------------------------------------------------------
Tbg97291270              ------------------------------------------------------------
Tbg97291300              ------------------------------------------------------------
Tb09v40146               ------------------------------------------------------------
Tb09v40147               ------------------------------------------------------------
Tb091601520              ------------------------------------------------------------
Tb09v40149               ------------------------------------------------------------
Tb09v40059               ------------------------------------------------------------
Tb09v40058               ------------------------------------------------------------
Tb09v40148               ------------------------------------------------------------
Tbg97291380              ------------------------------------------------------------
Tb09v40060               ------------------------------------------------------------
Tbg97291360              ------------------------------------------------------------
Tc00104705351105310      ------------------------------------------------------------
Tc00104705350763120      ------------------------------------------------------------
Tc00104705350401180      ------------------------------------------------------------
Tc001047053511859200     ------------------------------------------------------------
Tc00104705350601110      ------------------------------------------------------------
Tc00104705351104770      ------------------------------------------------------------
Tc00104705350453350      ------------------------------------------------------------
Tc001047053508735100     ------------------------------------------------------------
Tc0010475350894510       ------------------------------------------------------------
Tc00104705350863740      ------------------------------------------------------------
Tc00104705351112960      ------------------------------------------------------------
Tc00104705351191190      ------------------------------------------------------------
Tc00104705351024930      ------------------------------------------------------------
Tc00104705350735910      ------------------------------------------------------------
TvY486_0201870           ------------------------------------------------------------
TvY486_0600050           ------------------------------------------------------------
TvY486_0028480           ------------------------------------------------------------
TvY486_0201850           ------------------------------------------------------------
Tbg97223580              ------------------------------------------------------------
Tbg97223450              ------------------------------------------------------------
Tbg97223390              ------------------------------------------------------------
Tbg97223430              ------------------------------------------------------------
Tbg97223760              ------------------------------------------------------------
Tbg97223550              ------------------------------------------------------------
Tbg97223410              ------------------------------------------------------------
Tbg97223680              ------------------------------------------------------------
Tbg97223740              ------------------------------------------------------------
Tbg97223540              ------------------------------------------------------------
Tb92725360               ------------------------------------------------------------
Tb92725340               ------------------------------------------------------------
Tb92725330               ------------------------------------------------------------
Tbg97223600              ------------------------------------------------------------
Tbg97223520              ------------------------------------------------------------
Tbg97223370              ------------------------------------------------------------
Tbg97223470              ------------------------------------------------------------
Tbg97223700              ------------------------------------------------------------
Tbg97223620              ------------------------------------------------------------
Tbg97223490              ------------------------------------------------------------
Tbg97223660              ------------------------------------------------------------
Tbg97223640              ------------------------------------------------------------
Tb92725350               ------------------------------------------------------------
Tbg97223330              ------------------------------------------------------------
Tb92725290               ------------------------------------------------------------
Tbg97223720              ------------------------------------------------------------
Tb92725320               ------------------------------------------------------------
Tbg97223350              ------------------------------------------------------------
Tbg97223500              ------------------------------------------------------------
Tb92725300               ------------------------------------------------------------
Tb92725310               ------------------------------------------------------------
Tbg97222800              ------------------------------------------------------------
Tb92724760               ------------------------------------------------------------
Tbg97222940              ------------------------------------------------------------
Tb92724920               ------------------------------------------------------------
TcIL30002110             MVCCTIIT-----IIIISLLCLSEVKCSYSLSVCGGRCCYVLCRVTAYGVFLLREYCSPS
TcIL3000013740           MVCCTIIT-----IIIISLLCLSEVKCSYSLSVCGGRCCYVLCRVTAYGVFLLREYCSPL
TcIL3000027940           MVCCTIIT-----IIIISLLCLSEVKCSYSLSVCGGRCCYVLCRVTAYGVFLLREYCSPS
TcIL3000001110           -----------------------------------------------------MFHCALS
TcIL3000033420           MVCCTIITIIIIIIIIISLLCLSEVKCSYSLSTCGGRCCYVLCRVTACGVFLLREYCSPS
Tb9276380                ------------------------------------------------------------
Tbg972630                ------------------------------------------------------------
Tb9276370                ------------------------------------------------------------
```

```
TcIL3000050510          ------------------------------------------------------------
TcIL3000050500          ------------------------------------------------------------
TcIL3000016620          ------------------------------------------------------------
TcIL3000016630          ------------------------------------------------------------
TcIL3000047260          ------------------------------------------------------------


Tbg97291330             ------------------------------------------------------------
Tbg97291280             ------------------------------------------------------------
Tbg97291270             ------------------------------------------------------------
Tbg97291300             ------------------------------------------------------------
Tb09v40146              ------------------------------------------------------------
Tb09v40147              ------------------------------------------------------------
Tb091601520             ------------------------------------------------------------
Tb09v40149              ------------------------------------------------------------
Tb09v40059              ------------------------------------------------------------
Tb09v40058              ------------------------------------------------------------
Tb09v40148              ------------------------------------------------------------
Tbg97291380             ------------------------------------------------------------
Tb09v40060              ------------------------------------------------------------
Tbg97291360             ------------------------------------------------------------
Tc00104705351105310     ------------------------------------------------------------
Tc00104705350763120     ------------------------------------------------------------
Tc00104705350401180     ------------------------------------------------------------
Tc001047053511859200    ------------------------------------------------------------
Tc00104705350601110     ------------------------------------------------------------
Tc00104705351104770     ------------------------------------------------------------
Tc00104705350453350     ------------------------------------------------------------
Tc001047053508735100    ------------------------------------------------------------
Tc0010475350894510      ------------------------------------------------------------
Tc00104705350863740     ------------------------------------------------------------
Tc00104705351112960     ------------------------------------------------------------
Tc00104705351191190     ------------------------------------------------------------
Tc00104705351024930     ------------------------------------------------------------
Tc00104705350735910     ------------------------------------------------------------
TvY486_0201870          ------------------------------------------------------------
TvY486_0600050          ------------------------------------------------------------
TvY486_0028480          ------------------------------------------------------------
TvY486_0201850          ------------------------------------------------------------
Tbg97223580             ------------------------------------------------------------
Tbg97223450             ------------------------------------------------------------
Tbg97223390             ------------------------------------------------------------
Tbg97223430             ------------------------------------------------------------
Tbg97223760             ------------------------------------------------------------
Tbg97223550             ------------------------------------------------------------
Tbg97223410             ------------------------------------------------------------
Tbg97223680             ------------------------------------------------------------
Tbg97223740             ------------------------------------------------------------
Tbg97223540             ------------------------------------------------------------
Tb92725360              ------------------------------------------------------------
Tb92725340              ------------------------------------------------------------
Tb92725330              ------------------------------------------------------------
Tbg97223600             ------------------------------------------------------------
Tbg97223520             ------------------------------------------------------------
Tbg97223370             ------------------------------------------------------------
Tbg97223470             ------------------------------------------------------------
Tbg97223700             ------------------------------------------------------------
Tbg97223620             ------------------------------------------------------------
Tbg97223490             ------------------------------------------------------------
Tbg97223660             ------------------------------------------------------------
Tbg97223640             ------------------------------------------------------------
Tb92725350              ------------------------------------------------------------
Tbg97223330             ------------------------------------------------------------
Tb92725290              ------------------------------------------------------------
Tbg97223720             ------------------------------------------------------------
Tb92725320              ------------------------------------------------------------
Tbg97223350             ------------------------------------------------------------
Tbg97223500             ------------------------------------------------------------
Tb92725300              ------------------------------------------------------------
Tb92725310              ------------------------------------------------------------
Tbg97222800             ------------------------------------------------------------
Tb92724760              ------------------------------------------------------------
Tbg97222940             ------------------------------------------------------------
Tb92724920              ------------------------------------------------------------
TcIL30002110            ATCPLYYYYFFRDVSLCVELSLFSLPRCSACAGVHRSHCAFDVLPPPPRSLLSPPDSPEQ
TcIL3000013740          ATCPLYYYYFFRDVSLCVELSLFSLPRCSACAGVHRSHCAFDVLPPPPRSLLSPPDSPEQ
TcIL3000027940          ATCPLYYYYFFRDVLLCVELSLFSLPRCSACAGVHRSHCAFDVLPPPPRSLLSPPDSPEQ
TcIL3000001110          SS-------------------YFHFPAALPVLAFTGAIAIFDVLPPPTRSLLSPPDSPEQ
TcIL3000033420          ATRLLYYYYFFRDVSLCVELFLFSLPRCSACAGVHRGHCASDVLPPSHRSLLSPPDSPEQ
```

```
Tb9276380              ------------------------------------------------------------
Tbg972630              ------------------------------------------------------------
Tb9276370              ------------------------------------------------------------
TcIL3000050510         ------------------------------------------------------------
TcIL3000050500         ------------------------------------------------------------
TcIL3000016620         ------------------------------------------------------------
TcIL3000016630         ------------------------------------------------------------
TcIL3000047260         ------------------------------------------------------------


Tbg97291330            ------MEIRMVPSNFSR---ITTLCG---------VLMLLQCILRPLQAASSGG----G
Tbg97291280            ------MEIRMVPSNFSR---ITTLCG---------ILMLLQCILRPLQAASSGG----G
Tbg97291270            ------MEIRMVPSNFSR---ITTLCG---------VLMLLQCILRPLQAASSGG----G
Tbg97291300            ------MEIRMVPSNFSR---ITTLCG---------VLMLLQCILRPLQAASSGG----G
Tb09v40146             ------MEIRMVPSNFSR---ITTLCG---------ILMLLQCILRPLQAASSGG----G
Tb09v40147             ------MEIRMVPSNFSR---ITTLCG---------ILMLLQCILRPLQAASSGG----G
Tb091601520            ------MEIRMVPSNFSR---ITTLCG---------ILMLLQCILRPLQAASSGG----G
Tb09v40149             ------MEIRMVPSNFSR---ITTLCG---------ILMLLQCILRPLQAASSGG----G
Tb09v40059             ------MEIRMVPSNFSR---ITTLCG---------ILMLLQCFLRPLQAASSGG----G
Tb09v40058             ------MEIRMVPSNFSR---ITTLCG---------ILMLLQCILRPLQAASSGG----G
Tb09v40148             ------MEIRMVPSNFSR---ITTLCG---------ILMLLQCILRPLQAASSGG----G
Tbg97291380            ------MEIRMVPSNFSR---ITTLCG---------VLMLLQCILRPLQAASSGG----G
Tb09v40060             ------MEIRMVPSNFSR---ITTLCG---------ILMLLQCFLRPLQAASSGG----G
Tbg97291360            ------MEIRMVPSNFSR---ITTLCG---------ILMLLQCILRPLQAASSGG----G
Tc00104705351105310    ---MEFEAMGIRTRSATR---NAVRWM---------VLFLLLQYLGSHPSFTAAGE-EEK
Tc00104705350763120    ---MEFEAMGIRTRSATR---NAVRWM---------VLFLLLQYLGSHPSFTAAGE-EEK
Tc00104705350401180    --------MGIRMRSATR---NAVRWM---------VLFLLLQYLGSHPSFTAAGE-EEK
Tc001047053511859200   ---MEAEAMGIRTRSATR---NAVRWM---------VLFLLLQYLGSHPSFTAAG---EE
Tc00104705350601110    ---MEAEAMGIRTRSATR---NAVRWM---------VLFLLLQYLGSHPSFTAAGE-EEK
Tc00104705351104770    ---MESEAMGIRTRSATR---NAVRWM---------VLFLLLQYLGSHPSFTAAGE-EEK
Tc00104705350453350    ---MEVELMGMRTRSATR---NAVRWM---------VLFLLLQYLGSHPSFTAAGEEEEG
Tc001047053508735100   ---MEVELMGMRTRSATR---NAVRWM---------VLFLLLQYLGSHPSFTAAGEEEEG
Tc0010475350894510     ---MEVELMCMRTRSATR---NAVRWM---------VLFLLMQYLGSHPSFTAAGKEEEG
Tc00104705350863740    ---MEVELMCMRTRSATR---NAVRWM---------VLFLLMQYLGSHPSFTAAGEEEKG
Tc00104705351112960    ---MEVEFLGMRTRSATR---NAVRWM---------VLFLLLQYLGSHPSFTAAGSVEEG
Tc00104705351191190    ---MEVEFLGMRTRSATR---NAVRWT---------VLFLLLQYLGSHPSFTAAGKVEEG
Tc00104705351024930    ---MEVEFLGMRTRSATR---NAVRWM---------VLFLLLQYLGSHPSFTAAGKVEEG
Tc00104705350735910    ---MEFEAMGIRTRSATR---NAVRWM---------VLFLLLQYLGSHPSFTAAGKVEEG
TvY486_0201870         ------------------------------------------------------------
TvY486_0600050         ------------------------------------------------------------
TvY486_0028480         ----MTTDARARTAACVR---TAGAWP---------LLLLLCVHVAWHARLASAE---DG
TvY486_0201850         ----MTTDARARTAACVR---TAGAWP---------LLLLLCVHVACHARLASAE---DG
Tbg97223580            ----MSMDTRMGSFGFTR---AALLCG---------LLLLLTAVCPLHVS-AADGD--DG
Tbg97223450            ----MSMDTRMVSFGFTR---AALLCG---------LLLLLTAVCPLHVS-AADGD--DG
Tbg97223390            ----MSMDTRMVSFGFTR---AALLCG---------LLLLLTAVCPLHVS-AADGD--DG
Tbg97223430            ----MSMDTRMVSFGFTR---AALLCG---------LLLLLTAVCPLHVS-AADGD--DG
Tbg97223760            ----MSMDTRMVSFGFTR---AALLCG---------LLLLLTAVCPLHVS-AADGD--DG
Tbg97223550            ----MSMDTRMVSFGFTR---AALLCG---------LLLLLTAVCPLHVS-AADGD--DG
Tbg97223410            ----MSMDTRMVSFGFTR---AALLCG---------LLLLLTAVCPLHVS-AADGD--DG
Tbg97223680            ----MSMDTRMVSFGFTR---AALLCG---------LLLLLTAVCPLHVS-AADGD--DG
Tbg97223740            ----MSMDTRMVSFGFTR---AALLCG---------LLLLLTAVCPLHVS-AADGD--DG
Tbg97223540            ----MSMDTRMVSFGFTR---AALLCG---------LLLLLTAVCPLHVF-AADGD--DG
Tb92725360             ----MSMDTRMVNFGFTR---AALLCG---------ILLLLTAVCPLHVT-AADGD--DG
Tb92725340             ----MSMDTRMVNFGFTR---AALLCG---------ILLLLTAVCSLHVT-AADGD--DG
Tb92725330             ----MSMDTRMVNFGFTR---AALLCG---------ILLLLTAVCSLHVT-AADGD--DG
Tbg97223600            ----MSMDTRMVNFGFTR---AALLCG---------ILLLLTAVCPLHVT-AADGD--DG
Tbg97223520            ----MSMDTRMVNFGFTR---AALLCG---------ILLLLTAVCPLHVT-AADGD--DG
Tbg97223370            ----MSMDTRMVNFGFTR---AALLCG---------LLLLLTAVCPLRVT-AADGD--DG
Tbg97223470            ----MSMDTRMVNFGFTR---AALLCG---------LLLLLTAVCPLRVT-AADGD--DG
Tbg97223700            ----MSMDTRMVNFGFTR---AALLCG---------LLLLLTAVCPLRVT-AADGD--DG
Tbg97223620            ----MSMDTRMVNFGFTR---AALLCG---------LLLLLTAVCPLRVT-AADGD--DG
Tbg97223490            ----MSMDTRMVSFGFTR---AALLCG---------LLLLLTAVCPLRVT-AADGD--DG
Tbg97223660            ----MSMDTRMVSFGFTR---AALLCG---------LLLLLTAVCPLRVT-AADGD--DG
Tbg97223640            ----MSMDTRMVSFGFTR---AALLCG---------LLLLLTAVCPLRVT-AADGD--DG
Tb92725350             ----MSMDTRMVNFGFTR---AALLCG---------LLLLLTAVCPLHVT-AADGD--DG
Tbg97223330            ----MSMDTRMVNFGFTR---AALLCG---------LLLLLTAVCPLHVT-AADGD--DG
Tb92725290             ----MSMDTRMVNFGFTR---AALLCG---------LLLLLTAVCPLHVT-AAD----NG
Tbg97223720            ----MSMDTRMVSFGFTR---AALLCG---------LLLLLTAVCPLHVS-AADGD--DG
Tb92725320             ----MSMDTRMVNFGFTR---AALLCG---------ILLLLTAVCSLHVTAAADGD--DG
Tbg97223350            ----MSMDTRMVNFGFTR---AALLCG---------LLLLLTAVCPLHVT-AAADD--NG
Tbg97223500            ----------MVNFGFTR---AALLCG---------LLLLLTAVCPLHVT-AAADD--NG
Tb92725300             ----MSMDTRMVNFGFTR---AALLCG---------LLLLLTAVCPLHVT-AADGD--NG
Tb92725310             ----MSMDTRMVNFGFTR---AALLCG---------ILLLLTAVCPLHVT-AADGD---G
Tbg97222800            ----MSMDTRMVNFGFTR---AALLCG---------ILLLLTAVCPLHVT-AADGI--DG
Tb92724760             ----MSMDTRMVNFGFTR---AALLCG---------ILLLLTAVCSLHVT-AADGD--DG
Tbg97222940            ------MDTRMVNFGFTR---AALLCG---------ILLLLTAVCPLHVT-AAADD--DG
Tb92724920             ----MSMDTRMVNFGFTR---AALLCG---------ILLLLTAVCPLHVT-AADDD--DG
TcIL30002110           RKKEISKQAKISKTTMSSGRNALFACGAILRAVVA-LLLLQCVGRPLCLTASAT-----E
TcIL3000013740         RKKEISKQAKISKTTMSSGRNALFACGAILRAVVA-LLLLQCVGRPLCLTASAT-----E
```

```
TcIL3000027940          RKKEISKQAKISKTTMSSGRNALFACGAILRAVVA-LLLLQCVGRPLCLTASAT-----E
TcIL3000001110          RKKEISKKTKISKTTMSPGRNALFSCGTILRAVVALLLLLQCVGRPLCLTASAT-----E
TcIL3000033420          RKKEISKKAKISKTTMSPGRNALFACGAILRAVVA-LLLLQCVGRPLCLTASAD-----E
Tb9276380               ----MGTEISMTACGFAR---MVVLFT---------ALL-QCLSGSSHLFAFGK-----V
Tbg972630               ----MGTEISMTACGFAR---MVVLFT---------ALL-QCLSGSSHLFAFGK-----V
Tb9276370               ----MGTEISMTACGFAR---MVVLFT---------ALL-QCLSGSSHLFAFGK-----V
TcIL3000050510          ------------------------------------------------------------
TcIL3000050500          -----------MSKGTHA---APYALV---------LAL-LCVVHPLQVAVVAQ-----P
TcIL3000016620          -----------MSKGTHA---APYALV---------LVL-LCVVHPLQVAVVAQ-----P
TcIL3000016630          -----------MSKGTHA---TPCALV---------LVL-LCVVHPLQMAVSAK-----E
TcIL3000047260          -----------MSKGTHA---VPHVLV---------MAL-LCVVHPLQVAASAK-----E


Tbg97291330             PVTTAAKSYAIYTHEVFENNGVYNVDYVNSERVCRAEGMNLATDHSEATNSLIYKLLKPK
Tbg97291280             PVTTAAKSYAVYTHEVFENNGVYNVDYVNSERVCRAEGMNLATDHSEATNSLIYKLLKPK
Tbg97291270             PVTTAAKSYAIYTHEVFENNGVYNVDYVNSERVCRAEGMNLATDHSEATNSLIYKLLKPK
Tbg97291300             PVTTAAKSYAIYTHEVFENNGVYNVDYVNSERVCRAEGMNLATDHSEATNSLIYKLLKPK
Tb09v40146              PVTTAAKSYAVYPHEVFENNGVYNVDYVNSERVCRAEGMNLATDHSEATNSLIYKLLKPK
Tb09v40147              PVTTAAKSYAIYTHEVFENNGVYNVDYVNSERVCRAEGMNLATDHSEATNSLIYKLLKPK
Tb091601520             PVTTAAKSYAIYPHEVFKNNGVYNVDYVNSERVCRAEGMNLATDHSEATNSLIYKLLKPK
Tb09v40149              PVTTAAKSYAVYPHEVFKNNGVYNVDYVNSERVCRAEGMNLATDHSEATNSLIYKLLKPK
Tb09v40059              PVTTAAKSYAVYPHEVFENNGVYNVDYVNSERVCRAEGMNLATDHSEATNSLIYKLLKPK
Tb09v40058              PVTTAAKSYAVYPHEVFENNGVYNVDYVNSERVCRAEGMNLATDHSEATNSLIYKLLKPK
Tb09v40148              PVTTAAKSYAIYTHEVFENNGVYNVDYVNSERVCRAEGMNLATDHSEATNSLIYKLLKPK
Tbg97291380             PVTTAAKSYAVYPHEVFENNGVYNVDYVNSERVCRAEGMNLATDHSEATNSLIYKLLKPK
Tb09v40060              PVTTAAKSYAIYPHEVFKNNGVYNVDYVNSERVCRAEGMNLATDHSEATNSLIHKLLKPK
Tbg97291360             PVTTAAKSYAIYTHEVFENNGVYNVDYVNSERVCRAEGMNLATDHSEATNSLIYKLLKPK
Tc00104705351105310     HVTIGITKYFHHANSIFSSNFGGRVDYTNSQVSCFNGGGLLASDQTPAAHISIAAQLREL
Tc00104705350763120     HVTIGITKYFHYANSIFSSNFGGRVDYSNSQFSCFNGGGLLAADQTPAAHISIAAQLREL
Tc00104705350401180     HVTIGITKYFHHANSIFSSNFGGRVDYTNSQVSCFNGGGLLAADQTPAAHISIAAELREL
Tc001047053511859200    HVTIGITKYFHHAHSVFKTNFGYRVDYTNSQVSCFNGGGLLASDQTPAAHISIAAELRKL
Tc00104705350601110     HVTIGITKYFHYANSIFKTNFATRVDYTNSQVSCFNGGGLLASDQTPAAHISIAAQLREL
Tc00104705351104770     HVTIVITKYFHHANSIFSSNFGGRVDYTNSQVSCFNGGGLLASDQTPAAHVSIAAELREL
Tc00104705350453350     HVVIEIVKYFNYPNSIFFQNYAGDVSYFSSDDVCHLGGGVLSADQTPAAHVAIADELRKT
Tc001047053508735100    HVVIEIVKYFNYPNSIFFQNYAGDVSYFSSDDVCHLGGGVLSADQTPAAHVAIADELRKT
Tc0010475350894510      HVVIEIVRYFHYSNPIFFDNYAGDVSYFSSDDVCHLGGGVLSADQTPAAHAAIADELRKT
Tc00104705350863740     NVVIEIVKYFHYSNPIFFQNYAGDVSYFSSDDVCHLGGGVLSADQTPAAHVAIADELRKT
Tc00104705351112960     HVAIEIVRYFHYANPIFFENYAGDVSYFSSDDACHLGGGVLSADQTPAAHVAIAGELRKT
Tc00104705351191190     HVVIEIVKYFHYANPIFFENYAGDVSYFSSDDACHLGGGVLSADQTPAAHVAIAGELRKT
Tc00104705351024930     HVVIEIVKYFHYANPIFFENYAGDVSYFSSDDACHLGGGVLSADQTPAAHVAIAGELRKT
Tc00104705350735910     HVAIEIVKYFHYANPIFFENYAGDVSYFSSDDVCHLGGGVLSADQTPAAHVAIADELRKT
TvY486_0201870          ----------------------------------REAGAVLAADQSRAAHKAILQRFYAR
TvY486_0600050          ------------------------------------------------------------
TvY486_0028480          RVTVNVKRYLSLPSEGFRTQHAHRVDYSSSDRLCREAGAVLAADQSRAAHKAILEQFHVL
TvY486_0201850          RVTVNVRRYLSLPSESFRTAYDQRVDYSSSDRLCREAGAVLAADQSDAAHKAIFDQFHSY
Tbg97223580             RVIVNVKEYFSMPNRAFIRDGEYHVSYYNANAYCGDNGAVLPADQTEAAHQSLQDAMKRV
Tbg97223450             RVIVNVKEYFSMPNRAFIRDGEYHVSYYNANAYCGDNGAVLPADQTEAAHQSLQDAMKRV
Tbg97223390             RVIVNVKEYFSMPNRAFIRDGEYHVSYYNANAYCGDNGAVLPADQTEAAHQSLQDAMKRV
Tbg97223430             RVIVNVKEYFSMPNRAFIRDGEYHVSYYNANAYCGDNGAVLPADQTEAAHQSLQDAMKRV
Tbg97223760             RVIVNVKEYFSMPNRAFIRDGEYHVSYYNANAYCGDNGAVLPADQTEAAHQSLQDAMKRV
Tbg97223550             RVIVNVKEYFSMPNRAFIRDGEYHVSYYNANAYCGDNGAVLPADQTEAAHQSLQDAMKRV
Tbg97223410             RVIVNVKEYFSMPNRAFIRDGEYHVSYYNANAYCGDNGAVLPADQTEAAHQSLQDAMKRV
Tbg97223680             RVIVNVKEYFSMPNRAFIRDGEYHVSYYNANAYCGDNGAVLPADQTEAAHQSLQDAMKRV
Tbg97223740             RVIVNVKEYFSMPNRAFIRDGEYHVSYYNANAYCGDNGAVLPADQTEAAHQSLQDAMKRV
Tbg97223540             RVIVNVKEYFSMPNRAFIRDGEYHVSYYNANAYCGDNGAVLPADQTEAAHQSLQDAMKRV
Tb92725360              RVIVNVKEYFSMPNRAFIRDGEYHVSYYNANAYCVDNGAVLPADQTEAAHQSLQSAIKRV
Tb92725340              RVIVNVKEYFSMPNRAFIRDGEYHVSYYNANAYCVDNGAVLPADQTEAAHQILQDAMKRV
Tb92725330              RVIVNVKEYFSMPNRAFIRDGEYHVSYYNANAYCVDNGAVLPADQTEAAHQSLQDAMKRV
Tbg97223600             RVIVNINTIISLVNRAFIRDGEYYVSYYNANAYCGDNGAVLPADQTEAAHQSLQDAMKRV
Tbg97223520             RVIVNINTIISLVNRAFIRDGEYYVSYYNANAYCGDNGAVLPADQTEAAHQSLQDAMKRV
Tbg97223370             RVIVNINTIISMVNRAFIRDGEYYVSYYNANAYCGDNGAVLPADQTEAAHQSLQDAMKRV
Tbg97223470             RVIVNINTIISMVNRAFIRDGEYYVSYYNANAYCGDNGAVLPADQTEAAHQSLQDAMKRV
Tbg97223700             RVIVNINTIISMVNRAFIRDGEYYVSYYNANAYCGDNGAVLPADQTEAAHQSLQDAMKRV
Tbg97223620             RVIVNINTIISMVNRAFIRDGEYYVSYYNANAYCGDNGAVLPADQTEAAHQSLQDAMKRV
Tbg97223490             RVIVNINTIISMVNRAFIRDGEYYVSYYNANAYCVGNGAVLPADQTEAAHQILQDAIKRV
Tbg97223660             RVIVNINTIISMVNRAFIRDGEYYVSYYNANAYCVGNGAVLPADQTEAAHQILQDAIKRV
Tbg97223640             RVIVNINTIISMVNRAFIRDGEYYVSYYNANAYCVGNGAVLPADQTEAAHQILQDAIKRV
Tb92725350              RVIVNINTIISMVNRAFIRDGEYHVSYYNANAYCVDNGAVLPADQTEAAHQILQDAMKRV
Tbg97223330             RVIVNVKSYFSMDNFAFAHRGNYFVSYKNAEAYCVDNGAVLPADQTEAAHQSLQSAIKRV
Tb92725290              RVIVNVKSYFSMDNFAFAHRGNYFVSYKNAAAYCGDNGAVLPADQTEAAHQSLQSAIKRV
Tbg97223720             RVIVNVKSYFSMVYRAFFRNKTYFVSYENAEAYCVENGAVLPADQTEAAHQSLQSAIKRV
Tb92725320              RVIVNVKSYFSMVYRAFFRNKTYFVSYENAEAYCVENGAVLPADQTEAAHQSLQDAMKRV
Tbg97223350             RVIVNVKEYFSMPNYAFIRRRKYMVDQESTNRYCVYFGAEVAADQTEAAHQSLQDAMKRV
Tbg97223500             RVIVNVKEYFSMPNYAFIRRRKYMVDQESTNRYCVYFGAEVAADQTEAAHQSLQDAMKRV
Tb92725300              RVIVNVKEYFSMPNYAFIRRRKYMVDHESTNRYCVYFGAEVAADQTEAAHQSLQDAMKRV
Tb92725310              RVIVNVKTYFSVPTYAFIRKGRYAVNYENANYYCVFFGAVLPADQTEAAHQSLQDAMKRV
Tbg97222800             RVIVNVKEYFSMDNFAFSRHGKYFVSYNNAEAYCRDNGAVLPADQTEAAHQSLQDAMKDV
Tb92724760              RVIVNVKEYFSMDNFAFSRHGKYFVSYNNAEAYCRDNGAVLPADQTEAAHQSLQDAMKDV
Tbg97222940             RVIVNVKEYFSMDNFAFSRHERYIVGYDNAEAYCVDNGAVLPADQTEAAHKSLQDAIKRV
```

Tb92724920 RVIVNVKEYFSMDNFAFSRHERYIVGYDNAEAYCVDNGAVLPADQTEAAHQSLQDAMKSV
TcIL30002110 KVNVNVKRRFSASNYHFNRGERYMVDYRTSRQYCDQNGGVLVADQSDASHADIDKLLKAV
TcIL3000013740 KVNVNVKRRFSASNYHFERGESYMVDYRTSRQYCDQNGGVLVADQSDASHADIDKLLKAF
TcIL3000027940 KVNVNVKRRFRTSNFHFNRGERYMVDYRTSRQYCDQNGGVLVADQSDASHADIEKLLKAV
TcIL3000001110 KVNVNVKMRFDASNFNFNRGGRYMVDYKTARQYCEQNGAVLVADQSDASHADIDRLLKAF
TcIL3000033420 KVNVNVKMRFDASNFNFNHGGRYMVDYKTARQYCEQNGAVLVADQSDASHADIDRILKMI
Tb9276380 YPTAYVKEYILFPNRKFWHGGDYLVDYSNSVHICREAGGTLATDHSQATNERLTAYHLGV
Tbg972630 YPTAYVKEYILFPNRKFWHGGDYLVDYSNSVHICREAGGTLATDHSQATNECLTAYHLSV
Tb9276370 YPTAYVKEYILFPNRKFWHGGDYLVDYSNSVHICREAGGTLATDHSQATNERLTAYHLGV
TcIL3000050510 --
TcIL3000050500 GPIASTKDYIIFPNGRFWNGGQYLVSHGATERICREEGAILATDHSEATDAAIKEVHSTL
TcIL3000016620 GPAASTKDYIIFPNARFWNGGQYLVSHGATERICREEGAILATDHSEATDAAINEVYFKL
TcIL3000016630 GPIASTKEYIILPNGRFNEGGQYLVSHGATERICREEGAILATDHSEATDAAINEVHSTL
TcIL3000047260 GSTVSTKDYIILSDEKFQELGPNTAGHGLSKRACREEGATLATDHSEATGAAITKILKEL

Tbg97291330 NE--LGYLYVYLGGDAKYSASSVHEEKDRCKVGDLASSLNCVYRWNTGLFAPATPDDNGV
Tbg97291280 NE--LGYLYVYLGGDAKYSASSVHEEKDRCKVGDLASSLNCVYRWNTGLFAPATPDDNGV
Tbg97291270 NE--LGYLYVYLGGDAKYSASSVHEEKDRCKVGDLASSLNCVYRWNTGLFAPATPDDNGV
Tbg97291300 NE--LGYLYAYLGGDAKYSASSVHEEKDRCKVGDLASSLNCVYRWNTGLFAPATPDDNGV
Tb09v40146 NK--LGYLYAYLGGDAKYSASSVHEEKDRCKVGDLASSLNCVYRWNTGLFAPATPDDNGV
Tb09v40147 NK--LGYLYAYLGGDAKYSASSVHEEKDRCKVGDLASSLNCVYRWNTGLFAPATPDDNGV
Tb091601520 NE--LGYLYAYLGGDAKYSASSVHEEKDRCKVGDLASSLNCVYRWNTGLFAPATPDDNGV
Tb09v40149 NK--LGYLYAYLGGDAKYSASSVHEEKDRCKVGDLASSLNCVYRWNTGLFAPATPDDNGV
Tb09v40059 NK--LGYLYAYLGGDAKYSASSVHEEKDRCKVGDLASSLNCVYRWNTGLFAPATPDDNGV
Tb09v40058 NK--LGYLYAYLGGDAKYSASSVHEEKDRCKVGDLASSLNCVYRWNTGLFAPATPDDNGV
Tb09v40148 NK--LGYLYAYLGGDAKYSASSVHEEKDRCKVGDLASSLNCVYRWNTGLFAPATPDDNGV
Tbg97291380 NE--LGYLYAYLGGDAKYSASSVHEEKDRCKVGDLASSLNCVYRWNTGLFAPATPDDNGV
Tb09v40060 NK--LGYLYAYLGGDAKYSASSVHEEKDRCKVGDLASSLNCVYRWNTGLFAPATPDDNGV
Tbg97291360 NE--LGYLYAYLGGDAKYSASSVHEEKDRCKVGDLASSLNCVYRWNTGLFAPATPDDNGV
Tc00104705351105310 ASNKDETFRSYLGGTAEYTANSTP-PEQRCLRGERRASLKCIYKWNEGAFIDSSSDGHGV
Tc00104705350763120 ASNKDETFRSYLGGTAQYTANSAP-PENRCLRDERRASLKCIYKWNEGAFRDSSSDGHGV
Tc00104705350401180 ASNKDETFRSYLGGTAEYTANSTP-PEQRCLRGNKSASLKCIYKWNEGAFIDSSSDGHGV
Tc001047053511859200 ASKKVETFRTYLGGTAQYTADSAP-SEQRCLRGERRASLKCIYKWKEGAFLDSSSDGHGV
Tc00104705350601110 ASKEDETFRTYLGGTAEYTANSTP-PEQRCLRGERRASLKCIYKWNEGAFLDSSSDGHGV
Tc00104705351104770 ASKKVETFRSFLGGTAEYTADSAP-SEQRCLRGERRASLKCIYKWNEGAFLDSSSDGHGV
Tc00104705350453350 AVHNYDTSHAYLGGDADHAATPTTDPKKRCGRGLKWATLNCIFKWNQGAFADSSADGLGV
Tc001047053508735100 AVHNYDTSHAYLGGDADHAATPTTDPKKSCGRGLNLPTLNCIFKWNQGAFADSSADGLGV
Tc001047535O894510 AVRKYKTSHAYLGGDADHAATSTTDLKKRCGRGLKWATLNCIFKWNQGAFADSSADGLGV
Tc00104705350863740 VVPNYDTSYAYLGGDADHAATSTTDPKKRCGRGLKWATLNCIFKWNQGAFADSSDDGLGV
Tc00104705351112960 AVPNYDTSHVYLGGDADHEATPTTDPKKRCGRGLKWATLNCIFKWNKGAFADSSADGLGV
Tc00104705351191190 AVPKYKTSHVYLGGDADHEATPTTDPKKRCGRGLKWATLNCIFKWNKGAFADSSADGLGV
Tc00104705351024930 AVPKYKTSHVYLGGDADHEATPTTDPKKRCGRGLKWASLNCIFRWNKGAFADSSADGLGV
Tc00104705350735910 AVPNYDTSHVYLGGDADHEATPTTDPKKRCGRGLKWATLNCIFKWNQGAFADSSADGLGV
TvY486_0201870 K--NDRPLHSYLGGDARRSASTLEEPARRCKAGDEDASLNCVYRWNKGLFATST-HGRGA
TvY486_0600050 --
TvY486_0028480 S--VKSVGYSFLGGDALSNAAMRRNPLERCKAGDKTTSLNCVYRWNKGLFAAAS-NGDGA
TvY486_0201850 AK-NNQELYSFLGGDALSNAAVRREPTKWCKAGDKTTSLNCVYRWNKGLFATAS-NGDGA
Tbg97223580 IT--GSDAFSYLGGDAVYSSGGVIEADKRCKPGDIASSRYCVYRWNKGLFAKALPDGWGV
Tbg97223450 IT--GSDAFSYLGGDAVYSSGGVIEADKRCKPGDIASSRYCVYRWNKGLFAKALPDGWGV
Tbg97223390 IT--GSDAFSYLGGDAVYSSGGVIEADKRCKPGDIASSRYCVYRWNKGLFAKALPDGWGV
Tbg97223430 IT--GSDAFSYLGGDAVYSSGGVIEADKRCKPGDIASSRYCVYRWNKGLFAKALPDGWGV
Tbg97223760 IT--GSDAFSYLGGDAVYSSGGVIEADKRCKPGDIASSRYCVYRWNKGLFAKALPDGWGV
Tbg97223550 IT--GSDAFSYLGGDAVYSSGGAIVANKRCKPGDIASSRYCVYRWNKGLFAKALPDGWGV
Tbg97223410 IT--GSDAFSYLGGDAVYSSGGAIVANKRCKPGDIASSRYCVYRWNKGLFAKALPDGWGV
Tbg97223680 IT--GSDAFSYLGGDAVYSSGGAIVANKRCKPGDIASSRYCVYRWNKGLFAKALPDGWGV
Tbg97223740 IT--GSDAFSYLGGDAVYSSGGAIVANKRCKPGDIASSRYCVYRWNKGLFAKALPDGWGV
Tbg97223540 IT--GSDAFSYLGGDAVYSSGGAIEADKRCKPGDIASSRYCVYRWNKGLFAKALPDGWGV
Tb92725360 IT--GSDAFSYLGGDAVYSSGGVIEADKRCKPGDAASSRYCVYRWNKGLFAKALPDGWGV
Tb92725340 IA--ASDAFSYLGGDAVYSSGGAIVANKRCKPGDIASSRYCVYRWNKGLFAKALPDGWGV
Tb92725330 TN--SFVAFSYLGGDAVYSSGGVIEADKRCKPGDIASSRYCVYRWNKGLFAKALPDGWGV
Tbg97223600 IT--GSDAFSYLGGDAVYSSGGVIEADKRCKPGDIASSRYCVYRWNKGLFPKALPDGWGV
Tbg97223520 IT--GSDAFSYLGGDAVYSSGGVIEADKRCKPGDIASSRYCVYRWNKGLFPKALPDGWGV
Tbg97223370 IT--GSDAFSYLGGDAVYSSGGVIEADKRCKPGDIASSRYCVYRWNKGLFAKALPDGHGV
Tbg97223470 IT--GSDAFSYLGGDAVYSSGGVIEADKRCKPGDIASSRYCVYRWNKGLFAKALPDGHGV
Tbg97223700 IT--GSDAFSYLGGDAVYSSGGVIEADKRCKPGDIASSRYCVYRWNKGLFAKALPDGHGV
Tbg97223620 IT--GSDAFSYLGGDAVYSSGGVIEADKRCKPGDIASSRYCVYRWNKGLFAKALPDGWGV
Tbg97223490 IT--GSDAFSYLGGDAVYSSGEAIVADKRCKPGDAASSRYCVYRWNKGLFAKALPDGHGV
Tbg97223660 IT--GSDAFSYLGGDAVYSSGEAIVADKRCKPGDAASSRYCVYRWNKGLFAKALPDGHGV
Tbg97223640 IT--GSDAFSYLGGDAVYSSGEAIVANKRCKPGDIASSRYCVYRWNKGLFAKALPDGHGV
Tb92725350 TA--ASDAFSYLGGDAVYSSGGAIVANKRCKPGDIASSRYCVYRWNKGLFAKALPDGHGV
Tbg97223330 IT--GSDAFSYLGGDAVYSSGEAIEADKRCKPGDIASSRYCVYRWNKGLFAKALPDGQGV
Tb92725290 IT--GSDAFSYLGGDAVYSSGEAIEADKRCKPGDAASSRYCVYRWNKGLFAKALPDGQGV
Tbg97223720 YS--IFVAFSYLGGDAVYSSSGVIEFNKRCNAGDIASSRYCVYRWNKGLFAKALPDGHGV
Tb92725320 YS--IFVAFSYLGGDAVYSSSGVIEFNKRCNAGDIASSRYCVYRWNKGLFAKVLPDGHGV
Tbg97223350 IT--GSDAFSYLGGDAVYSSGEAIEADKRCKPGDIASSRYCVYRWNKGLFAKALPDGHGV
Tbg97223500 IT--GSDAFSYLGGDAVYSSGEAIEADKRCKPGDIASSRYCVYRWNKGLFAKALPDGHGV
Tb92725300 IT--GSDAFSYLGGDAVYSSGEAIEADKRCKPGDAASSRYCVYRWNKGLFAKALPDGHGV
Tb92725310 IT--GSDAFSYLGGDAVYSSGEAIEADKRCKPGDIASSRYCVYRWNKGLFAKALPDGWGV

```
Tbg97222800            LT--GSDAFSYLGGDAVYSSGGAIVADKRCKPGDAASSRHCVYRWNKGLFAKALPDGHGV
Tb92724760             LT--GSDAFSYLGGDAVYSSGGAIVADKRCKPGDAASSRHCVYRWNKGLFAKALPDGHGV
Tbg97222940            IT--GSVAFSYLGGDAVYSSGGVIEADKRCKPGDAASSRHCVYRWNKGLFAKALPDGHGV
Tb92724920             IF--EFEAVSYLGGDAVYSSTRVFEADKRCKPGDAASSRHCVYRWNKGLFAKALPDGHGV
TcIL30002110           AW--NGNVVAYLGGDASYSARPPSSRWDRCVAGDFRTSKYCVYRWNQGLFAGSQPGGQGV
TcIL3000013740         AW--NGNVVAYLGGDASYSARPPSSRWDKCVAGYFRTSKYCVYRWNQGLFADSQPGGQGV
TcIL3000027940         AW--NGNVVAYLGGDASYSARPPSSRWDKCVAGDFRTSKYCVYRWNQGLFADSQPGGQGV
TcIL3000001110         AW--SRNVIAYLGGDALYSTGPPSSRWGRCVAGYFRTSKYCVYRWNQGLFADSQPGGQGV
TcIL3000033420         AW--SKNVISYLGGDALYSTGPPSSRWDRCAAGYLSVSRYCVYRWNQGLFADSQPGGQGV
Tb9276380              A--DGPFIYSYLGGDATYSASAAQDAGIRCTAGDYDTSLNCVYRWNSGLFEASGIEGNGV
Tbg972630              A--DGPFIYSYLGGDATYSASAAQDAGSRCTAGDYDTSLNCVYRWNSGLFEASGIEGNGV
Tb9276370              A--DGPFIYSYLGGDATYSASAAQDAGIRCTAGDYDTSLNCVYRWNSGLFEASGIEGNGV
TcIL3000050510         ------------------------------------------------------------
TcIL3000050500         P--QVY-IYSYLGGDATFSASDAQDAGSRCKERNSSTSIHCVYRWNRGLFDATTTNGVGE
TcIL3000016620         R--QRY-IYSYLGGDAAFSASEAQDVGSRCKEADNSTSIHCVYRWNRGLFDATTTNGIGE
TcIL3000016630         S--HVHNIYSYLGGDATFSASEAQDAGSRCKEADNSTSIHCVYRWNRGLFDATTTNGIGE
TcIL3000047260         P--YFF-PISYLGGDATLSASEAQDAGSRCRSGDKSTSIHCVYRWNTGLFEATTTNGIGE


Tbg97291330            AFWRGSSHEVSGAGSMNDYPSFFKN-YPAYGRLNVIAKLDSSD-RVTWFDDDDDFGN---
Tbg97291280            AFWRGSSHEVSGAGSMNDYPSFFKN-YPAYGRLNVIAKLDSSD-RVTWFDDDDDFGN---
Tbg97291270            AFWRGSYHEVSGAGSMNDYPSFFKN-YPAYGRLNVIAKLDSSD-RVTWFDDDDDFGN---
Tbg97291300            AFWRGSYHEVSGAGSMNDYPSFFKN-YPAYGRLNVIAKLDSSD-RVTWFDDDDDFGN---
Tb09v40146             AFWRGSYYEVTGAGSMNDYPSFFEN-YPAYGRLNVIAKLDSSD-RVTWFDDDDDFGN---
Tb09v40147             AFWRGSYYEVTGAGSMNDYPSFFEN-YPAYGRLNVIAKLDSSD-RVTWFDDDDDFGN---
Tb091601520            AFWRGSYYEVTGAGSMNDYPSFFEN-YPAYGRLNVIAKLDSSG-RFTWFDDDDDFGN---
Tb09v40149             AFWRGSYYEVTGAGSMNDYPSFFEN-YPAYGRLNVIAKLDSSG-RFTWFDDDDDFGN---
Tb09v40059             AFWRGSYYEVTGAGSMNDYPSFFEN-YPAYGRLNVIAKLDSSG-RFTWFDDDDDFGN---
Tb09v40058             AFWRGSYYEVTGAGSMNDYPSFFEN-YPAYGRLNVIAKLDSSG-RFTWFDDDDDFGN---
Tb09v40148             AFWRGSYYEVTGAGSMNDYPSFFEN-YPAYGRLNVIAKLDSSG-RFTWFDDDDDFGN---
Tbg97291380            AFWRGSYHEVSGAGSMNDYPSFFKN-YPAYGRLNVIAKLDPSD-RVTWFDDDDDFGH---
Tb09v40060             AFWRGSYYEVTGAGSMNDYPSFFEN-YPAYGRLNVIAKLDPSD-RVTWFDDDDDFGN---
Tbg97291360            AFWRGSYHEVSGAGSMNDYPSFFKN-YPAYGRLNVIAKLDSSD-RVTWFDDDDDFGN---
Tc00104705351105310    SFFRGTVYTLIGAKAMNGY-------------------------DAYWGPKLPGHG----
Tc00104705350763120    SFFRGTMYTLIGAKAMNGY-------------------------DAYWGPKLPGHG----
Tc00104705350401180    SFFRGTVYTLIDAGPMNGY-------------------------DAYWGPELPKHG----
Tc001047053511859200   SFFRGTMYTLIGAGPMNGY-------------------------DAYWGNKLPGHG----
Tc00104705350601110    SFFRGTMYTLIGAGPMNGY-------------------------DAYWGNKLPGHG----
Tc00104705351104770    SFFRGTMYTLIGAKAMNGY-------------------------DAYWGPKLPGHG----
Tc00104705350453350    AFFRGTLYTLVDVGPMNGY-------------------------KAYWPYHYPHHG----
Tc001047053508735100   AFFRGTLYTLVGVGPMNGY-------------------------KAYWPYHYPHHG----
Tc0010475350894510     AFFRGTLYTLVDAGPMNGY-------------------------KAYWPYHYPHHG----
Tc00104705350863740    AFFRGTLYTLVGVGPMNGY-------------------------KAYWPYHYPFHG----
Tc00104705351112960    AFFRGTLYTLVGVGPMNGY-------------------------KAYWPYQYPYHG----
Tc00104705351191190    AFFRGTLYTLVGVGPMNGY-------------------------KAYWPYHYPYHG----
Tc00104705351024930    AFFRGTLYTLVDAGPMNGY-------------------------KAYWPYQYPHHG----
Tc00104705350735910    AFFRGTLYTLVDAGPMNGY-------------------------KAYWPYQYPHHG----
TvY486_0201870         AFWRGSYRSVPGSGKLNGYNAEWRSHHPRNGGLNIVSRTDPDVLWFTWADIIERSSR---
TvY486_0600050         -----------------------------------IVSRTDPDVLWFTWADIIERSSR---
TvY486_0028480         AFWRGSLRDVSGSAALNGYPSHWSELYPKDSYLNVVAWYLPDKYAVYWRDVNSDQP----
TvY486_0201850         AFWRGSLYGVSGSAAMNGYPPHWNELYPKDSYLNIVECHLNDKHAMYWRDVNSAQP----
Tbg97223580            AFWHGSYVKYGGAASMNGYPSFWGSEYPKRGQLYTLSWYHKDYDVTTWYDGKDMSGR---
Tbg97223450            AFWHGSYVKYGGAASMNGYPSFWGSEYPKRGQLYTLSWYHKDYDVTTWYDGKDMSGR---
Tbg97223390            AFWHGSYVKYGGAASMNGYPSFWGSEYPKRGQLYTLSWYHKDYDVTTWYDGKDMSGR---
Tbg97223430            AFWHGSYVKYGGAASMNGYPSFWGSEYPKRGQLYTLSWYHKDYDVTTWYDGKDMSGR---
Tbg97223760            AFWHGSYVKYGGAASMNGYPSFWGSEYPKRGQLYTLSWYHKDYDVTTWYDGKDMSGR---
Tbg97223550            AFWHGSYVKYGGAASMNGYPSFWGSEYPKRGQLYTLSWYHKDYDVTTWYDGKDMSGR---
Tbg97223410            AFWHGSYVKYGGAASMNGYPSFWGSEYPKRGQLYTLSWYHKDYDVTTWYDGKDMSGR---
Tbg97223680            AFWHGSYVKYGGAASMNGYPSFWGSEYPKRGQLYTLSWYHKDYDVTTWYDGKDMSGR---
Tbg97223740            AFWHGSYVKYGGAASMNGYPSFWGSEYPKRGQLYTLSWYHKDYDVTTWYDGKDMSGR---
Tbg97223540            AFWHGSYVKYGGAASMNGYPSFWGSEYPKRGQLYTLSWYHKDYDVTTWYDGKDMSGR---
Tb92725360             AFWRGSYVKYGGAASMNGYPSFWGSEYPKRGQLYTLSWYHKDYDVTTWYDGKDMSGR---
Tb92725340             AFWHGSYVKYGGAASMNGYPSFWGSEYPKHGQLYTLSWYHKDYDVTTWYDGKDMSGR---
Tb92725330             AFWHGSYVKYGGAASMNGYPSFWGSEYPKRGQLYTLSWYHKDYDVTTWYDGKDMSGR---
Tbg97223600            AFWHGSYVKYGGAASMNGYPSFWGSEYPKRGQLYTLSWYHKDYDVTTWYDGKDMSGR---
Tbg97223520            AFWHGSYVKYGGAASMNGYPSFWGSEYPKRGQLYTLSWYHKDYDVTTWYDGKDMSGR---
Tbg97223370            AFWHGSYVKYGGAASMNGYPSFWGSEYPKRGQLYTLSWYHKDYDVTTWYDGKDMSGR---
Tbg97223470            AFWHGSYVKYGGAASMNGYPSFWGSEYPKRGQLYTLSWYHKDYDVTTWYDGKDMSGR---
Tbg97223700            AFWHGSYVKYGGAASMNGYPSFWGSEYPKRGQLYTLSWYHKDYDVTTWYDGKDMSGR---
Tbg97223620            AFWHGSYVKYGGAASMNGYPSFWGSEYPKRGQLYTLSWYHKDYDVTTWYDGKDMSGR---
Tbg97223490            AFWHGSYVKYIFAGPMNGYPSFWGGEHPKRGQLYTLSWYHKDYDVTTWYDGKDMSGR---
Tbg97223660            AFWHGSYVKYIFAGPMNGYPSFWGGEHPKRGQLYTLSWYHKDYDVTTWYDGKDMSGR---
Tbg97223640            AFWHGSYVKYIFAGPMNGYPSFWGGEHPKRGQLYTLSWYHKDYDVTTWYDGKDMSGR---
Tb92725350             AFWHGSYVKYIFAGPMNGYPSFWGGEHPKRGQLYTLSWYHKDYDVTTWYDGKDMSGR---
Tbg97223330            AFWRGSYIKYDGAASMNGYPSFWGGEYPKRGQLYTLSWYHTGDDVTTWYDGKDMSGR---
Tb92725290             AFWRGSYVKYGGAASMNGYPSFWGSEYPKRGQLYTLSWYHTGDDVTTWYDGKDMSGR---
Tbg97223720            AFWHGSYVKYDGAASMNGYPSFWGSEYPKRGQLYTLSWYHKDYDVTTWYDAESVAGQ---
Tb92725320             AFWHGSYVKYDGAASMNGYPSFWGSEYPKRGQLYTLSWYHEHEDVTTWYDAESVAGQ---
Tbg97223350            AFWHGSYVKYGGAASMNGYPSFWGGEYPKRGQLYTLSWYHTGDDVTTWYDADGVSGT---
```

```
Tbg97223500                AFWHGSYVKYGGAASMNGYPSFWGGEYPKRGQLYTLSWYHTGDDVTTWYDADGVSGT---
Tb92725300                 AFWHGSYVKYDGAASMNGYPSFWGSEYPKRGQLYTLSWYHTGDNSTTWYDADGVSGT---
Tb92725310                 AFWHGSYVKYDGAASMNGYPSFWGSEYPKRGQLYTLSWYHTGDNSTTWYDADGVSGT---
Tbg97222800                AFWRGSYVKYAFAAPMNGYPSFWGSEYPKHGQLYTLSWYHEDDDVTTWYDGREASGT---
Tb92724760                 AFWRGSYVKYAFAAPMNGYPSFWGSEYPKHGQLYTLSWYHEDDDVTTWYDGREASGT---
Tbg97222940                AFWRGSYVKYAFAAPMNGYPSFWGSEYPKRGQLYTLSWYHEDDDVTTWYDGREASGT---
Tb92724920                 AFWRGSYVKYAFAAPMNGYPSFWGSEYPKSGQLYTLSWYHEHEDVTTWYDGREASGT---
TcIL30002110               SFWRGSFQRDPSAEAMNGYPRYWRGDYPNRGQLYVLAWRLMNDVDTLWYDGGEVSGS---
TcIL3000013740             SFWRGSFQRDPSAEAMNGYPRYWRGDYPNRGQLYVLAWRLMNDVDTLWYDGREVSGS---
TcIL3000027940             SFWRGSFQRDPSAEAMNGYPRYWRGDYPNRGQLYVLAWRLMNDVDTLWYDGREVSGS---
TcIL3000001110             SFWRGSFQRDPSAEAMNGYPRYWRGDYPNRGQLYVLAWRLMNDVDTLWYDGGEVSGS---
TcIL3000033420             SFWRGSFQRDPTAEAMNGYPRYWRGDYPNRGQLFVLAWRLMNDVDTLWYDGREVSGS---
Tb9276380                  AFWRGSYTYKSGSGALGGYPSFFAQSYPQDGQLHVMGWYTNVTKWFGWFD----------
Tbg972630                  AFWRGSYTYKSGSGALGGYPSFFAQSYPQDGQLHVMGWYTNVTKWFGWFD----------
Tb9276370                  AFWRGSYTYKSGSGALGGYPSFFAQSYPQDGQLHVMGWYTNVTKWFGWFD----------
TcIL3000050510             ---------------MNDLPSRWLNTYPKYGFLYXISRRYSTESNRLWFDEPEDTHSKYA
TcIL3000050500             AFWRGSYRSTPGAGSMNDLPSRWLNTYPKYGFLYPISRRYSTESNRLWFDEPEDTHSKYA
TcIL3000016620             AFWRGSYRSTPGAGSMNDFPFRWIKDYPKFGFLYPISRRHYRESKRTWFDEYEDTGHEAA
TcIL3000016630             AFWRGSYRSTPGAGSMNDFPFRWIKDYPKFGVLYPMSRRHYRESKRTWFD----------
TcIL3000047260             AFWRGSYKRMPGAGSMNDFPPRWHPQFPGAGLLYVDSGYAKIFAGSVWFDKNE-TQQALI
                                                                          *

Tbg97291330                ------------------------------------------------------------
Tbg97291280                ------------------------------------------------------------
Tbg97291270                ------------------------------------------------------------
Tbg97291300                ------------------------------------------------------------
Tb09v40146                 ------------------------------------------------------------
Tb09v40147                 ------------------------------------------------------------
Tb091601520                ------------------------------------------------------------
Tb09v40149                 ------------------------------------------------------------
Tb09v40059                 ------------------------------------------------------------
Tb09v40058                 ------------------------------------------------------------
Tb09v40148                 ------------------------------------------------------------
Tbg97291380                ------------------------------------------------------------
Tb09v40060                 ------------------------------------------------------------
Tbg97291360                ------------------------------------------------------------
Tc00104705351105310        ------------------------------------------------------------
Tc00104705350763120        ------------------------------------------------------------
Tc00104705350401180        ------------------------------------------------------------
Tc001047053511859200       ------------------------------------------------------------
Tc00104705350601110        ------------------------------------------------------------
Tc00104705351104770        ------------------------------------------------------------
Tc00104705350453350        ------------------------------------------------------------
Tc001047053508735100       ------------------------------------------------------------
Tc0010475350894510         ------------------------------------------------------------
Tc00104705350863740        ------------------------------------------------------------
Tc00104705351112960        ------------------------------------------------------------
Tc00104705351191190        ------------------------------------------------------------
Tc00104705351024930        ------------------------------------------------------------
Tc00104705350735910        ------------------------------------------------------------
TvY486_0201870             ------------------------------------------------------------
TvY486_0600050             ------------------------------------------------------------
TvY486_0028480             ------------------------------------------------------------
TvY486_0201850             ------------------------------------------------------------
Tbg97223580                ------------------------------------------------------------
Tbg97223450                ------------------------------------------------------------
Tbg97223390                ------------------------------------------------------------
Tbg97223430                ------------------------------------------------------------
Tbg97223760                ------------------------------------------------------------
Tbg97223550                ------------------------------------------------------------
Tbg97223410                ------------------------------------------------------------
Tbg97223680                ------------------------------------------------------------
Tbg97223740                ------------------------------------------------------------
Tbg97223540                ------------------------------------------------------------
Tb92725360                 ------------------------------------------------------------
Tb92725340                 ------------------------------------------------------------
Tb92725330                 ------------------------------------------------------------
Tbg97223600                ------------------------------------------------------------
Tbg97223520                ------------------------------------------------------------
Tbg97223370                ------------------------------------------------------------
Tbg97223470                ------------------------------------------------------------
Tbg97223700                ------------------------------------------------------------
Tbg97223620                ------------------------------------------------------------
Tbg97223490                ------------------------------------------------------------
Tbg97223660                ------------------------------------------------------------
Tbg97223640                ------------------------------------------------------------
Tb92725350                 ------------------------------------------------------------
Tbg97223330                ------------------------------------------------------------
Tb92725290                 ------------------------------------------------------------
```

```
Tbg97223720             ------------------------------------------------------------
Tb92725320              ------------------------------------------------------------
Tbg97223350             ------------------------------------------------------------
Tbg97223500             ------------------------------------------------------------
Tb92725300              ------------------------------------------------------------
Tb92725310              ------------------------------------------------------------
Tbg97222800             ------------------------------------------------------------
Tb92724760              ------------------------------------------------------------
Tbg97222940             ------------------------------------------------------------
Tb92724920              ------------------------------------------------------------
TcIL30002110            ------------------------------------------------------------
TcIL3000013740          ------------------------------------------------------------
TcIL3000027940          ------------------------------------------------------------
TcIL3000001110          ------------------------------------------------------------
TcIL3000033420          ------------------------------------------------------------
Tb9276380               ------------------------------------------------------------
Tbg972630               ------------------------------------------------------------
Tb9276370               ------------------------------------------------------------
TcIL3000050510          YTVRAERPVGFYVALCNIYVFTTSAPVPQPEST---------------------------
TcIL3000050500          YTVRAERPVGFYVALCNIYVFTTSAPVPQPEST---------------------------
TcIL3000016620          HVVRKEKQVKNFVLLCNIYVPTTSAPVPQPES----------------------------
TcIL3000016630          ------------------------------------------------------------
TcIL3000047260          YEVFRFRTTHLILCQLQNKKPAPLPPSPGPATADNEELNGTDANETASEELNGTDANETA

Tbg97291330             ------------------------------------------------------------
Tbg97291280             ------------------------------------------------------------
Tbg97291270             ------------------------------------------------------------
Tbg97291300             ------------------------------------------------------------
Tb09v40146              ------------------------------------------------------------
Tb09v40147              ------------------------------------------------------------
Tb091601520             ------------------------------------------------------------
Tb09v40149              ------------------------------------------------------------
Tb09v40059              ------------------------------------------------------------
Tb09v40058              ------------------------------------------------------------
Tb09v40148              ------------------------------------------------------------
Tbg97291380             ------------------------------------------------------------
Tb09v40060              ------------------------------------------------------------
Tbg97291360             ------------------------------------------------------------
Tc00104705351105310     ------------------------------------------------------------
Tc00104705350763120     ------------------------------------------------------------
Tc00104705350401180     ------------------------------------------------------------
Tc001047053511859200    ------------------------------------------------------------
Tc00104705350601110     ------------------------------------------------------------
Tc00104705351104770     ------------------------------------------------------------
Tc00104705350453350     ------------------------------------------------------------
Tc001047053508735100    ------------------------------------------------------------
Tc0010475350894510      ------------------------------------------------------------
Tc00104705350863740     ------------------------------------------------------------
Tc00104705351112960     ------------------------------------------------------------
Tc00104705351191190     ------------------------------------------------------------
Tc00104705351024930     ------------------------------------------------------------
Tc00104705350735910     ------------------------------------------------------------
TvY486_0201870          ------------------------------------------------------------
TvY486_0600050          ------------------------------------------------------------
TvY486_0028480          ------------------------------------------------------------
TvY486_0201850          ------------------------------------------------------------
Tbg97223580             ------------------------------------------------------------
Tbg97223450             ------------------------------------------------------------
Tbg97223390             ------------------------------------------------------------
Tbg97223430             ------------------------------------------------------------
Tbg97223760             ------------------------------------------------------------
Tbg97223550             ------------------------------------------------------------
Tbg97223410             ------------------------------------------------------------
Tbg97223680             ------------------------------------------------------------
Tbg97223740             ------------------------------------------------------------
Tbg97223540             ------------------------------------------------------------
Tb92725360              ------------------------------------------------------------
Tb92725340              ------------------------------------------------------------
Tb92725330              ------------------------------------------------------------
Tbg97223600             ------------------------------------------------------------
Tbg97223520             ------------------------------------------------------------
Tbg97223370             ------------------------------------------------------------
Tbg97223470             ------------------------------------------------------------
Tbg97223700             ------------------------------------------------------------
Tbg97223620             ------------------------------------------------------------
Tbg97223490             ------------------------------------------------------------
Tbg97223660             ------------------------------------------------------------
Tbg97223640             ------------------------------------------------------------
```

```
Tb92725350              ------------------------------------------------------------
Tbg97223330             ------------------------------------------------------------
Tb92725290              ------------------------------------------------------------
Tbg97223720             ------------------------------------------------------------
Tb92725320              ------------------------------------------------------------
Tbg97223350             ------------------------------------------------------------
Tbg97223500             ------------------------------------------------------------
Tb92725300              ------------------------------------------------------------
Tb92725310              ------------------------------------------------------------
Tbg97222800             ------------------------------------------------------------
Tb92724760              ------------------------------------------------------------
Tbg97222940             ------------------------------------------------------------
Tb92724920              ------------------------------------------------------------
TcIL30002110            ------------------------------------------------------------
TcIL3000013740          ------------------------------------------------------------
TcIL3000027940          ------------------------------------------------------------
TcIL3000001110          ------------------------------------------------------------
TcIL3000033420          ------------------------------------------------------------
Tb9276380               ------------------------------------------------------------
Tbg972630               ------------------------------------------------------------
Tb9276370               ------------------------------------------------------------
TcIL3000050510          ----------ETESEEHNGTNTNETESEELNGTNANETASEELNGTNVNETAGEELNGTN
TcIL3000050500          ----------ETESEEHNGTNTNETASEELNGTNANETASEELNGTNVNETAGEELNGTN
TcIL3000016620          ------------------------------------------------------------
TcIL3000016630          ------------------------------------------------------------
TcIL3000047260          SEELNGTDANETASEELNGTNANETASEELNGTDANETASEELNGTNANETASEELNGTD


Tbg97291330             ----------------------------------------NISFTNTRARSTRFFMVLCE
Tbg97291280             ----------------------------------------NISFTNTRARSTRFFMVLCE
Tbg97291270             ----------------------------------------NISFTNTRARSTRFFMVLCE
Tbg97291300             ----------------------------------------NISFTNTRARSTRFFMVLCE
Tb09v40146              ----------------------------------------NISFTNTRARSTRFFMVLCE
Tb09v40147              ----------------------------------------NISFTNTRARSTRFFMVLCE
Tb091601520             ----------------------------------------ISMFTNTRGRSTRFFMVLCE
Tb09v40149              ----------------------------------------ISMFTNTRGRSTRFFMVLCE
Tb09v40059              ----------------------------------------ISMFTNTRGRSTRFFMVLCE
Tb09v40058              ----------------------------------------ISMFTNTRGRSTRFFMVLCE
Tb09v40148              ----------------------------------------ISMFTNTRGRSTRFFMVLCE
Tbg97291380             ----------------------------------------ISPFTNVRGRSIYYFKVLCE
Tb09v40060              ----------------------------------------NINLMNVRSAVSRDIYILCE
Tbg97291360             ----------------------------------------NINLMNVRSAVGRGICILCE
Tc00104705351105310     ------------------------------------------------QLFLMFRMDTNQ
Tc00104705350763120     ------------------------------------------------QLFLMFRMDTNQ
Tc00104705350401180     ------------------------------------------------QLFLMFRMDTNQ
Tc001047053511859200    ------------------------------------------------QLFLMLRMDTSQ
Tc00104705350601110     ------------------------------------------------QLFLMLRMDTSQ
Tc00104705351104770     ------------------------------------------------QLFLMFRMDTSQ
Tc00104705350453350     ------------------------------------------------KTFLMLRLKKKE
Tc001047053508735100    ------------------------------------------------KTFLMLRLKTRE
Tc0010475350894510      ------------------------------------------------KTFLILRLDMMD
Tc00104705350863740     ------------------------------------------------KTFLMLRLNTTD
Tc00104705351112960     ------------------------------------------------KTFLMLRLKTTD
Tc00104705351191190     ------------------------------------------------KTFLILRLKTTD
Tc00104705351024930     ------------------------------------------------KTFLMLRLKTTD
Tc00104705350735910     ------------------------------------------------KTFLMLRLKTTD
TvY486_0201870          ----------------------------------------SSAKVPIEKTLLSNYIAVCE
TvY486_0600050          ----------------------------------------SSAKVPIEKTLLSNYIAVCE
TvY486_0028480          ----------------------------------------YFAPTPILYTYVTNYIAVCE
TvY486_0201850          ----------------------------------------HYAQAPIQGTYVTSYIAVCE
Tbg97223580             ----------------------------------------IKWASDNPDTKTENYVILCE
Tbg97223450             ----------------------------------------IKWASDNPDTKTENYVILCE
Tbg97223390             ----------------------------------------IKWASDNPDTKTENYVILCE
Tbg97223430             ----------------------------------------IKWASDNPDTKTENYVILCE
Tbg97223760             ----------------------------------------IKWASDNPDTKTENYVILCE
Tbg97223550             ----------------------------------------IKWASDNPDTKTENYVILCE
Tbg97223410             ----------------------------------------IKWASDNPDTKTENYVILCE
Tbg97223680             ----------------------------------------IKWASDNPDTKTENYVILCE
Tbg97223740             ----------------------------------------IKWASDNPDTKTENYVILCE
Tbg97223540             ----------------------------------------IKWASDNPDTKTENYVILCE
Tb92725360              ----------------------------------------IKWASDNPDTKTENYVILCE
Tb92725340              ----------------------------------------IKGTSKNPATKTENYVILCE
Tb92725330              ----------------------------------------IKWTSKNTATRTENYVILCE
Tbg97223600             ----------------------------------------IKGTSKNPATKTENYVILCE
Tbg97223520             ----------------------------------------IKGTSKNPATKTENYVILCE
Tbg97223370             ----------------------------------------IKGTSKNPATKTENYVILCE
Tbg97223470             ----------------------------------------IKGTSKNPATKTENYVILCE
Tbg97223700             ----------------------------------------IKGTSKNPATKTENYVILCE
Tbg97223620             ----------------------------------------IKWTSKNPATKTENYVILCE
```

```
Tbg97223490           ----------------------------------------IKGTSKNPATKTENYVILCE
Tbg97223660           ----------------------------------------IKGTSKNPATKTENYVILCE
Tbg97223640           ----------------------------------------IKGTSKNPATKTENYVILCE
Tb92725350            ----------------------------------------IKGTSKNPATKTENYVILCE
Tbg97223330           ----------------------------------------IKWASDNPDTKTENYVILCE
Tb92725290            ----------------------------------------IKWASDNPDTKTENYVILCE
Tbg97223720           ----------------------------------------LVAASNNPETTNKNYVILCE
Tb92725320            ----------------------------------------LVAASNNPETTNKNYVILCE
Tbg97223350           ----------------------------------------IRWTSRNFDTKSQDYVIVCE
Tbg97223500           ----------------------------------------IRWTSRNFDTKSQDYVIVCE
Tb92725300            ----------------------------------------IRWTSRNFDTKSQDYVIVCE
Tb92725310            ----------------------------------------IRWTSRNFDTKSQDYVVTCE
Tbg97222800           ----------------------------------------IRWTSQNFETKSQDYVVVCE
Tb92724760            ----------------------------------------IRWTSQNFETKSQDYVVVCE
Tbg97222940           ----------------------------------------IRWTSQNFETKSQDYVVVCE
Tb92724920            ----------------------------------------IRWTSQNFETKSQDYVVVCE
TcIL30002110          ----------------------------------------NRRSSRNPGTKSEEYRILCE
TcIL3000013740        ----------------------------------------DRRSSRNPGTKSEEYRILCE
TcIL3000027940        ----------------------------------------DRKSSRNPGTKSEEYHILCE
TcIL3000001110        ----------------------------------------DRKTSRNPGTKSGEYHILCE
TcIL3000033420        ----------------------------------------DRKTSRNPGTKSDEYRMLCE
Tb9276380             --------------------------------------------GGEVSGNKPGGKAPGY
Tbg972630             --------------------------------------------GGEVSGNKPGGKAPGY
Tb9276370             --------------------------------------------GGEVSGNKPGGKAPGY
TcIL3000050510        ASETAGEELNGTNTNETASEELNGTNTNETASEGLNGTNANETASEELNGTNTNETASEE
TcIL3000050500        ASETAGEELNGTSANETASEELNGTNANETASEELNGTNANETASEELNGTNANETASEE
TcIL3000016620        ----------------------D-----ENASEELNGTNANETESEELNGTNTNETESED
TcIL3000016630        -------------------------------------------VTLETAGSSTEGSVLDE
TcIL3000047260        ANETASEELNGTDANETASEELNGTNANETASEELNGTDANETASEELNGTDTNETASEE

Tbg97291330           ASAVPTPLPP------------------------------------------ASPHSENT
Tbg97291280           ASAVPTPLPP------------------------------------------ASPHSENT
Tbg97291270           ASAVPTPLPP------------------------------------------ASPHSENT
Tbg97291300           ASAVPTPLPP------------------------------------------ASPHSENT
Tb09v40146            ASAVPTPLPP------------------------------------------ASPHSENT
Tb09v40147            ASAVPTPLPP------------------------------------------ASPHSENT
Tb091601520           ASAVPTPLPP------------------------------------------ASPHSENT
Tb09v40149            ASAVPTPLPP------------------------------------------ASPHSENT
Tb09v40059            ASAVPTPLPP------------------------------------------ASPHSENT
Tb09v40058            ASAVPTPLPP------------------------------------------ASPHSENT
Tb09v40148            ASAVPTPLPP------------------------------------------ASPHSENT
Tbg97291380           ASAVPTPLPP------------------------------------------ASPHSENT
Tb09v40060            TSLIVAVAAENLIDADTFVVNERKFVNDKQSSGSNTSAVGNITVREGKRPDGNTSEDKNT
Tbg97291360           TSLTVALAAENLFATDTFAVNERKFVNDKQCSGSNTSAVGNISVREGKKPGENTSDDKNT
Tc00104705351105310   NDEKATWYDG-------------------------------------------------Q
Tc00104705350763120   NDEKATWYDG-------------------------------------------------Q
Tc00104705350401180   SDEKATWYDG-------------------------------------------------Q
Tc001047053511859200  SDKKATWYDG-------------------------------------------------Q
Tc00104705350601110   SDKNATWYDG-------------------------------------------------Q
Tc00104705351104770   SYEKATWYDG-------------------------------------------------Q
Tc00104705350453350   NSGNATWYDW-------------------------------------------------Y
Tc001047053508735100  NSEQATWYDW-------------------------------------------------Y
Tc0010475350894510    NSEKATWYDW-------------------------------------------------Y
Tc00104705350863740   NSENATWYDW-------------------------------------------------Y
Tc00104705351112960   NNGNATWYDW-------------------------------------------------Y
Tc00104705351191190   NNEQATWYDW-------------------------------------------------Y
Tc00104705351024930   NNGNATWYDW-------------------------------------------------Y
Tc00104705350735910   NSGNATWYDW-------------------------------------------------Y
TvY486_0201870        VQDSISTSTT-------------------------------------------------T
TvY486_0600050        VQDSISTSTT-------------------------------------------------T
TvY486_0028480        VQDSVSTTGT-------------------------------------------------G
TvY486_0201850        SQDSISSSTT-------------------------------------------------T
Tbg97223580           VHDSIS---T-------------------------------------------------T
Tbg97223450           VHDSIS---T-------------------------------------------------T
Tbg97223390           VHDSIS---T-------------------------------------------------T
Tbg97223430           VHDSIS---T-------------------------------------------------T
Tbg97223760           VHDSIS---T-------------------------------------------------T
Tbg97223550           VHDSIS---T-------------------------------------------------T
Tbg97223410           VHDSIS---T-------------------------------------------------T
Tbg97223680           VHDSIS---T-------------------------------------------------T
Tbg97223740           VHDSIS---T-------------------------------------------------T
Tbg97223540           VHDSIS---T-------------------------------------------------T
Tb92725360            VHDSISTT-T-------------------------------------------------T
Tb92725340            VHDSISTT-T-------------------------------------------------T
Tb92725330            VHDSISTT-T-------------------------------------------------T
Tbg97223600           VHDSIS------------------------------------------------------
Tbg97223520           VHDSIS------------------------------------------------------
Tbg97223370           VHDSIS------------------------------------------------------
```

```
Tbg97223470              VHDSIS------------------------------------------------------
Tbg97223700              VHDSIS------------------------------------------------------
Tbg97223620              VHDSIS------------------------------------------------------
Tbg97223490              VHDSIS------------------------------------------------------
Tbg97223660              VHDSIS------------------------------------------------------
Tbg97223640              VHDSIS------------------------------------------------------
Tb92725350               VHDSISTT-T-------------------------------------------------T
Tbg97223330              VHDSISTT-T-------------------------------------------------T
Tb92725290               VHDSISTT-T-------------------------------------------------T
Tbg97223720              VHDSIST-----------------------------------------------------
Tb92725320               VHDSIS------------------------------------------------------
Tbg97223350              VQRYVTVA-A-------------------------------------------------D
Tbg97223500              VQRYVTVA-A-------------------------------------------------D
Tb92725300               VQRYVTVA-A-------------------------------------------------D
Tb92725310               VQTDIITT-T-------------------------------------------------T
Tbg97222800              VHDSIS------------------------------------------------------
Tb92724760               VHDSIST-----------------------------------------------------
Tbg97222940              VHDSIST-----------------------------------------------------
Tb92724920               VHDSIS------------------------------------------------------
TcIL30002110             VHQSIAKPTT-------------------------------------------------T
TcIL3000013740           VHQSIAKPTT-------------------------------------------------T
TcIL3000027940           VHQSIAKPTT-------------------------------------------------T
TcIL3000001110           VHQSIAKS----------------------------------------------------
TcIL3000033420           VHRSIAKP----------------------------------------------------
Tb9276380                KSLTYFAALC-------------------------------------------------E
Tbg972630                KSLTYFAALC-------------------------------------------------E
Tb9276370                KSLTYFAALC-------------------------------------------------E
TcIL3000050510           LNGTNTNETA-------------------------------------------------S
TcIL3000050500           LNGTNANETA-------------------------------------------------S
TcIL3000016620           LNGTNTNETA-------------------------------------------------S
TcIL3000016630           RNSTLT------------------------------------------------------
TcIL3000047260           LNGTDTNETA-------------------------------------------------S


Tbg97291330              TADEN-------TTVDGNTNADG-------------------------------------
Tbg97291280              TADEN-------TTVDGNTNAD--------------------------------------
Tbg97291270              TADEN-------TTADGNTNADE-------------------------------------
Tbg97291300              TADEN-------TTADENTTADG-------------------------------------
Tb09v40146               TADEN-------TTADENTTVDE-------------------------------------
Tb09v40147               TADEN-------TTVDENTTVDE-------------------------------------
Tb091601520              TADEN-------TTVDENTTVDE-------------------------------------
Tb09v40149               TADEN-------TTADENTTADE-------------------------------------
Tb09v40059               TVDEN-------TTADENTTVDE-------------------------------------
Tb09v40058               TVDEN-------TTADENTTVDE-------------------------------------
Tb09v40148               TADEN-------TTADGNTTVDE-------------------------------------
Tbg97291380              TADEN-------TTADGNTTADGNITTDGNTNADGNTNADGNTTADGNTTADGNTNADGN
Tb09v40060               TSHENRSYDD-SSVADGNTSEDKNTTSHENSSHDDSSVADGNTSEDKNTTSHENSSHDDS
Tbg97291360              TSHENRSYND-SSVADGNTSDDESTTSHENRSYDDSSVADGNTSDDESTTSHENRSYDDS
Tc00104705351105310      G--------------VPKYDKE--------------------------------------
Tc00104705350763120      G--------------VPRGDGE--------------------------------------
Tc00104705350401180      G--------------VPKYDKE--------------------------------------
Tc001047053511859200     G--------------VPRYDGE--------------------------------------
Tc00104705350601110      G--------------VPRYDGE--------------------------------------
Tc00104705351104770      G--------------VPWYDGE--------------------------------------
Tc00104705350453350      P--------------APKDHGD--------------------------------------
Tc001047053508735100     P--------------APKTHID--------------------------------------
Tc0010475350894510       P--------------APKTHID--------------------------------------
Tc00104705350863740      P--------------APKDHID--------------------------------------
Tc00104705351112960      P--------------APKDHTD--------------------------------------
Tc00104705351191190      P--------------APKDHTD--------------------------------------
Tc00104705351024930      P--------------APKDHTD--------------------------------------
Tc00104705350735910      P--------------APKDHTD--------------------------------------
TvY486_0201870           T-----TTTTTTTTTTTTTTTTSTT-----------------------------------
TvY486_0600050           T-----TTTTTTTTTTTTTTTT--------------------------------------
TvY486_0028480           NGE---SASLASGAKLPFTSTNDAG-----------------------------------
TvY486_0201850           TRAPTTTTTTTTTTTEAPRTTTTST-----------------------------------
Tbg97223580              T----------TTTTTTTTTTT--------------------------------------
Tbg97223450              T----------TTTTTTTTTTT--------------------------------------
Tbg97223390              T----------TTTTTTTTTTT--------------------------------------
Tbg97223430              T----------TTTTTTTTTTT--------------------------------------
Tbg97223760              T----------TTTTTTTTTTT--------------------------------------
Tbg97223550              T----------TTTTTTTTTTT--------------------------------------
Tbg97223410              T----------TTTTTTTTTTT--------------------------------------
Tbg97223680              T----------TTTTTTTTTTT--------------------------------------
Tbg97223740              T----------TTTTTTTTTTT--------------------------------------
Tbg97223540              T----------TTTTTTTTTTT--------------------------------------
Tb92725360               T----------TTTTTTTTTTT--------------------------------------
Tb92725340               T----------TTTTTTTTTTT--------------------------------------
Tb92725330               T----------TTTTTTTTTTT--------------------------------------
```

```
Tbg97223600            ------------TTTTTTTTTT--------------------------------------
Tbg97223520            ------------TTTTTTTTTT--------------------------------------
Tbg97223370            ------------TTTTTTTTTT--------------------------------------
Tbg97223470            ------------TTTTTTTTTT--------------------------------------
Tbg97223700            ------------TTTTTTTTTT--------------------------------------
Tbg97223620            ------------TTTTTTTTTT--------------------------------------
Tbg97223490            ------------TTTTTTTTTT--------------------------------------
Tbg97223660            ------------TTTTTTTTTT--------------------------------------
Tbg97223640            ------------TTTTTTTTTT--------------------------------------
Tb92725350             T----------TTTTTTTTTTT--------------------------------------
Tbg97223330            T----------TTTTTTTTTTT--------------------------------------
Tb92725290             T----------TTTTTTTTTTT--------------------------------------
Tbg97223720            ------------TTTTTTTTTT--------------------------------------
Tb92725320             ------------TTTTTTTTTT--------------------------------------
Tbg97223350            N----------YTTTTTTTTTTTT------------------------------------
Tbg97223500            N----------YTTTTTTTTTTTT------------------------------------
Tb92725300             N----------YTTTTTT------------------------------------------
Tb92725310             ------------TTTTTT------------------------------------------
Tbg97222800            ------------TTTTTTTTTT--------------------------------------
Tb92724760             -----------TTTTTTTTTTT--------------------------------------
Tbg97222940            -------------TTTTTTTTT--------------------------------------
Tb92724920             ----------------TTTTTT--------------------------------------
TcIL30002110           T----------TTTTTTTTTTT--------------------------------------
TcIL3000013740         T----------TTTTTTTTTTT--------------------------------------
TcIL3000027940         T----------TTTTTTTTTTT--------------------------------------
TcIL3000001110         -----------TTTTTTTTTTT--------------------------------------
TcIL3000033420         -----------TTTTTTTTTTT--------------------------------------
Tb9276380              VT--------VPQPSHPVPGNGNS------------------------------------
Tbg972630              VT--------VPQPSNPVPGNGNSP-----------------------------------
Tb9276370              VT--------VPQPSHPVPGNGNS------------------------------------
TcIL3000050510         EE--------LNGTNANETASEELNGTNTNETASEGLNGTNANETASEELNGTNA-----
TcIL3000050500         EE--------LNGTNANETASEELNGTNANETASEELNGTNANETASEELNGTNA-----
TcIL3000016620         EE--------LNGTNTNETASEELN-------------GTNANETASEELNGTNA-----
TcIL3000016630         G---------IVGHIQHETPVAPPS------------------------P----------
TcIL3000047260         EE--------LNGTDTNETASEELNGTDTNETASEELNGTNANETASEELNGTDANETAS

Tbg97291330            ------------------------------------------------------------
Tbg97291280            ------------------------------------------------------------
Tbg97291270            ------------------------------------------------------------
Tbg97291300            ------------------------------------------------------------
Tb09v40146             ------------------------------------------------------------
Tb09v40147             ------------------------------------------------------------
Tb091601520            ------------------------------------------------------------
Tb09v40149             ------------------------------------------------------------
Tb09v40059             ------------------------------------------------------------
Tb09v40058             ------------------------------------------------------------
Tb09v40148             ------------------------------------------------------------
Tbg97291380            TTADG-------------------------------------------------------
Tb09v40060             SVADG-------------------------------------------------------
Tbg97291360            SVADGNSSDDESTSDDESTSDYESTAGENTNADGNTSNEKNTTTHEYRSYDDSSVADGNT
Tc00104705351105310    ------------------------------------------------------------
Tc00104705350763120    ------------------------------------------------------------
Tc00104705350401180    ------------------------------------------------------------
Tc001047053511859200   ------------------------------------------------------------
Tc00104705350601110    ------------------------------------------------------------
Tc00104705351104770    ------------------------------------------------------------
Tc00104705350453350    ------------------------------------------------------------
Tc001047053508735100   ------------------------------------------------------------
Tc0010475350894510     ------------------------------------------------------------
Tc00104705350863740    ------------------------------------------------------------
Tc00104705351112960    ------------------------------------------------------------
Tc00104705351191190    ------------------------------------------------------------
Tc00104705351024930    ------------------------------------------------------------
Tc00104705350735910    ------------------------------------------------------------
TvY486_0201870         ------------------------------------------------------------
TvY486_0600050         ------------------------------------------------------------
TvY486_0028480         ------------------------------------------------------------
TvY486_0201850         ------------------------------------------------------------
Tbg97223580            ------------------------------------------------------------
Tbg97223450            ------------------------------------------------------------
Tbg97223390            ------------------------------------------------------------
Tbg97223430            ------------------------------------------------------------
Tbg97223760            ------------------------------------------------------------
Tbg97223550            ------------------------------------------------------------
Tbg97223410            ------------------------------------------------------------
Tbg97223680            ------------------------------------------------------------
Tbg97223740            ------------------------------------------------------------
Tbg97223540            ------------------------------------------------------------
```

```
Tb92725360            ------------------------------------------------------------
Tb92725340            ------------------------------------------------------------
Tb92725330            ------------------------------------------------------------
Tbg97223600           ------------------------------------------------------------
Tbg97223520           ------------------------------------------------------------
Tbg97223370           ------------------------------------------------------------
Tbg97223470           ------------------------------------------------------------
Tbg97223700           ------------------------------------------------------------
Tbg97223620           ------------------------------------------------------------
Tbg97223490           ------------------------------------------------------------
Tbg97223660           ------------------------------------------------------------
Tbg97223640           ------------------------------------------------------------
Tb92725350            ------------------------------------------------------------
Tbg97223330           ------------------------------------------------------------
Tb92725290            ------------------------------------------------------------
Tbg97223720           ------------------------------------------------------------
Tb92725320            ------------------------------------------------------------
Tbg97223350           ------------------------------------------------------------
Tbg97223500           ------------------------------------------------------------
Tb92725300            ------------------------------------------------------------
Tb92725310            ------------------------------------------------------------
Tbg97222800           ------------------------------------------------------------
Tb92724760            ------------------------------------------------------------
Tbg97222940           ------------------------------------------------------------
Tb92724920            ------------------------------------------------------------
TcIL30002110          ------------------------------------------------------------
TcIL3000013740        ------------------------------------------------------------
TcIL3000027940        ------------------------------------------------------------
TcIL3000001110        ------------------------------------------------------------
TcIL3000033420        ------------------------------------------------------------
Tb9276380             ------------------------------------------------------------
Tbg972630             ------------------------------------------------------------
Tb9276370             ------------------------------------------------------------
TcIL3000050510        ------------------------------------------------------------
TcIL3000050500        ------------------------------------------------------------
TcIL3000016620        ------------------------------------------------------------
TcIL3000016630        ------------------------------------------------------------
TcIL3000047260        EELNGTDTNETASE---------------------EL-----------------------

Tbg97291330           ------------------------------------------------------------
Tbg97291280           ------------------------------------------------------------
Tbg97291270           ------------------------------------------------------------
Tbg97291300           ------------------------------------------------------------
Tb09v40146            ------------------------------------------------------------
Tb09v40147            ------------------------------------------------------------
Tb091601520           ------------------------------------------------------------
Tb09v40149            ------------------------------------------------------------
Tb09v40059            ------------------------------------------------------------
Tb09v40058            ------------------------------------------------------------
Tb09v40148            ------------------------------------------------------------
Tbg97291380           ------------------------------------------------------------
Tb09v40060            ------------------------------------------------------------
Tbg97291360           SDDESTTSHENRSYDDSSVADGNSSGDESTTSHENRSYNDSSVADGNTSDDESTTSHENR
Tc00104705351105310   ------------------------------------------------------------
Tc00104705350763120   ------------------------------------------------------------
Tc00104705350401180   ------------------------------------------------------------
Tc001047053511859200  ------------------------------------------------------------
Tc00104705350601110   ------------------------------------------------------------
Tc00104705351104770   ------------------------------------------------------------
Tc00104705350453350   ------------------------------------------------------------
Tc001047053508735100  ------------------------------------------------------------
Tc0010475350894510    ------------------------------------------------------------
Tc00104705350863740   ------------------------------------------------------------
Tc00104705351112960   ------------------------------------------------------------
Tc00104705351191190   ------------------------------------------------------------
Tc00104705351024930   ------------------------------------------------------------
Tc00104705350735910   ------------------------------------------------------------
TvY486_0201870        ------------------------------------------------------------
TvY486_0600050        ------------------------------------------------------------
TvY486_0028480        ------------------------------------------------------------
TvY486_0201850        ------------------------------------------------------------
Tbg97223580           ------------------------------------------------------------
Tbg97223450           ------------------------------------------------------------
Tbg97223390           ------------------------------------------------------------
Tbg97223430           ------------------------------------------------------------
Tbg97223760           ------------------------------------------------------------
Tbg97223550           ------------------------------------------------------------
Tbg97223410           ------------------------------------------------------------
```

```
Tbg97223680            ------------------------------------------------------------
Tbg97223740            ------------------------------------------------------------
Tbg97223540            ------------------------------------------------------------
Tb92725360             ------------------------------------------------------------
Tb92725340             ------------------------------------------------------------
Tb92725330             ------------------------------------------------------------
Tbg97223600            ------------------------------------------------------------
Tbg97223520            ------------------------------------------------------------
Tbg97223370            ------------------------------------------------------------
Tbg97223470            ------------------------------------------------------------
Tbg97223700            ------------------------------------------------------------
Tbg97223620            ------------------------------------------------------------
Tbg97223490            ------------------------------------------------------------
Tbg97223660            ------------------------------------------------------------
Tbg97223640            ------------------------------------------------------------
Tb92725350             ------------------------------------------------------------
Tbg97223330            ------------------------------------------------------------
Tb92725290             ------------------------------------------------------------
Tbg97223720            ------------------------------------------------------------
Tb92725320             ------------------------------------------------------------
Tbg97223350            ------------------------------------------------------------
Tbg97223500            ------------------------------------------------------------
Tb92725300             ------------------------------------------------------------
Tb92725310             ------------------------------------------------------------
Tbg97222800            ------------------------------------------------------------
Tb92724760             ------------------------------------------------------------
Tbg97222940            ------------------------------------------------------------
Tb92724920             ------------------------------------------------------------
TcIL30002110           ------------------------------------------------------------
TcIL3000013740         ------------------------------------------------------------
TcIL3000027940         ------------------------------------------------------------
TcIL3000001110         ------------------------------------------------------------
TcIL3000033420         ------------------------------------------------------------
Tb9276380              ------------------------------------------------------------
Tbg972630              ------------------------------------------------------------
Tb9276370              ------------------------------------------------------------
TcIL3000050510         ------------------------------------------------------------
TcIL3000050500         ------------------------------------------------------------
TcIL3000016620         ------------------------------------------------------------
TcIL3000016630         ------------------------------------------------------------
TcIL3000047260         ------------------------------------------------------------


Tbg97291330            ------------------------------------------------------------
Tbg97291280            ------------------------------------------------------------
Tbg97291270            ------------------------------------------------------------
Tbg97291300            ------------------------------------------------------------
Tb09v40146             ------------------------------------------------------------
Tb09v40147             ------------------------------------------------------------
Tb091601520            ------------------------------------------------------------
Tb09v40149             ------------------------------------------------------------
Tb09v40059             ------------------------------------------------------------
Tb09v40058             ------------------------------------------------------------
Tb09v40148             ------------------------------------------------------------
Tbg97291380            ------------------------------------------------------------
Tb09v40060             ------------------------------------------------------------
Tbg97291360            SYDDSSVADGNTSDDESTTSHENRSYDDSSVAMGTQVTMKVLPMMRVLVTMSTAGENTKA
Tc00104705351105310    ------------------------------------------------------------
Tc00104705350763120    ------------------------------------------------------------
Tc00104705350401180    ------------------------------------------------------------
Tc001047053511859200   ------------------------------------------------------------
Tc00104705350601110    ------------------------------------------------------------
Tc00104705351104770    ------------------------------------------------------------
Tc00104705350453350    ------------------------------------------------------------
Tc001047053508735100   ------------------------------------------------------------
Tc0010475350894510     ------------------------------------------------------------
Tc00104705350863740    ------------------------------------------------------------
Tc00104705351112960    ------------------------------------------------------------
Tc00104705351191190    ------------------------------------------------------------
Tc00104705351024930    ------------------------------------------------------------
Tc00104705350735910    ------------------------------------------------------------
TvY486_0201870         ------------------------------------------------------------
TvY486_0600050         ------------------------------------------------------------
TvY486_0028480         ------------------------------------------------------------
TvY486_0201850         ------------------------------------------------------------
Tbg97223580            ------------------------------------------------------------
Tbg97223450            ------------------------------------------------------------
Tbg97223390            ------------------------------------------------------------
Tbg97223430            ------------------------------------------------------------
```

```
Tbg97223760            ------------------------------------------------------------
Tbg97223550            ------------------------------------------------------------
Tbg97223410            ------------------------------------------------------------
Tbg97223680            ------------------------------------------------------------
Tbg97223740            ------------------------------------------------------------
Tbg97223540            ------------------------------------------------------------
Tb92725360             ------------------------------------------------------------
Tb92725340             ------------------------------------------------------------
Tb92725330             ------------------------------------------------------------
Tbg97223600            ------------------------------------------------------------
Tbg97223520            ------------------------------------------------------------
Tbg97223370            ------------------------------------------------------------
Tbg97223470            ------------------------------------------------------------
Tbg97223700            ------------------------------------------------------------
Tbg97223620            ------------------------------------------------------------
Tbg97223490            ------------------------------------------------------------
Tbg97223660            ------------------------------------------------------------
Tbg97223640            ------------------------------------------------------------
Tb92725350             ------------------------------------------------------------
Tbg97223330            ------------------------------------------------------------
Tb92725290             ------------------------------------------------------------
Tbg97223720            ------------------------------------------------------------
Tb92725320             ------------------------------------------------------------
Tbg97223350            ------------------------------------------------------------
Tbg97223500            ------------------------------------------------------------
Tb92725300             ------------------------------------------------------------
Tb92725310             ------------------------------------------------------------
Tbg97222800            ------------------------------------------------------------
Tb92724760             ------------------------------------------------------------
Tbg97222940            ------------------------------------------------------------
Tb92724920             ------------------------------------------------------------
TcIL30002110           ------------------------------------------------------------
TcIL3000013740         ------------------------------------------------------------
TcIL3000027940         ------------------------------------------------------------
TcIL3000001110         ------------------------------------------------------------
TcIL3000033420         ------------------------------------------------------------
Tb9276380              ------------------------------------------------------------
Tbg972630              ------------------------------------------------------------
Tb9276370              ------------------------------------------------------------
TcIL3000050510         ------------------------------------------------------------
TcIL3000050500         ------------------------------------------------------------
TcIL3000016620         ------------------------------------------------------------
TcIL3000016630         ------------------------------------------------------------
TcIL3000047260         ------------------------------------------------------------

Tbg97291330            --------------------------------------------------NTNADENTTA
Tbg97291280            ------------------------------------------------------------
Tbg97291270            --------------------------------------------------N------TTA
Tbg97291300            --------------------------------------------------N------TNA
Tb09v40146             --------------------------------------------------NTTADENTTA
Tb09v40147             --------------------------------------------------NTTADENTTA
Tb091601520            --------------------------------------------------NTTADENTTV
Tb09v40149             --------------------------------------------------N---------
Tb09v40059             --------------------------------------------------N---------
Tb09v40058             --------------------------------------------------N---------
Tb09v40148             --------------------------------------------------NTTADENTTV
Tbg97291380            N--------------------------------------------ITTDGNTNADGNTTA
Tb09v40060             --NTSEDKNTTSHENRSYDDSSVADGNTSEDKNTTSHENRSYDDSSVADGNTSDDKNTTS
Tbg97291360            DGNTSDDESTTSHENRSYDDSSVADGNTSDDKSTTSHENRSYDDSSVADGNTSDDKNTTS
Tc00104705351105310    ------------------------------------------------------------
Tc00104705350763120    ------------------------------------------------------------
Tc00104705350401180    ------------------------------------------------------------
Tc001047053511859200   ------------------------------------------------------------
Tc00104705350601110    ------------------------------------------------------------
Tc00104705351104770    ------------------------------------------------------------
Tc00104705350453350    ------------------------------------------------------------
Tc001047053508735100   ------------------------------------------------------------
Tc0010475350894510     ------------------------------------------------------------
Tc00104705350863740    ------------------------------------------------------------
Tc00104705351112960    ------------------------------------------------------------
Tc00104705351191190    ------------------------------------------------------------
Tc00104705351024930    ------------------------------------------------------------
Tc00104705350735910    ------------------------------------------------------------
TvY486_0201870         ------------------------------------------------------------
TvY486_0600050         ------------------------------------------------------------
TvY486_0028480         ------------------------------------------------------------
TvY486_0201850         ------------------------------------------------------------
Tbg97223580            ------------------------------------------------------------
```

```
Tbg97223450             ------------------------------------------------------------
Tbg97223390             ------------------------------------------------------------
Tbg97223430             ------------------------------------------------------------
Tbg97223760             ------------------------------------------------------------
Tbg97223550             ------------------------------------------------------------
Tbg97223410             ------------------------------------------------------------
Tbg97223680             ------------------------------------------------------------
Tbg97223740             ------------------------------------------------------------
Tbg97223540             ------------------------------------------------------------
Tb92725360              ------------------------------------------------------------
Tb92725340              ------------------------------------------------------------
Tb92725330              ------------------------------------------------------------
Tbg97223600             ------------------------------------------------------------
Tbg97223520             ------------------------------------------------------------
Tbg97223370             ------------------------------------------------------------
Tbg97223470             ------------------------------------------------------------
Tbg97223700             ------------------------------------------------------------
Tbg97223620             ------------------------------------------------------------
Tbg97223490             ------------------------------------------------------------
Tbg97223660             ------------------------------------------------------------
Tbg97223640             ------------------------------------------------------------
Tb92725350              ------------------------------------------------------------
Tbg97223330             ------------------------------------------------------------
Tb92725290              ------------------------------------------------------------
Tbg97223720             ------------------------------------------------------------
Tb92725320              ------------------------------------------------------------
Tbg97223350             ------------------------------------------------------------
Tbg97223500             ------------------------------------------------------------
Tb92725300              ------------------------------------------------------------
Tb92725310              ------------------------------------------------------------
Tbg97222800             ------------------------------------------------------------
Tb92724760              ------------------------------------------------------------
Tbg97222940             ------------------------------------------------------------
Tb92724920              ------------------------------------------------------------
TcIL30002110            ------------------------------------------------------------
TcIL3000013740          ------------------------------------------------------------
TcIL3000027940          ------------------------------------------------------------
TcIL3000001110          ------------------------------------------------------------
TcIL3000033420          ------------------------------------------------------------
Tb9276380               ------------------------------------------------------------
Tbg972630               ----------------------------------------------------------AN
Tb9276370               ------------------------------------------------------------
TcIL3000050510          -------------------------------------------------------NETAS
TcIL3000050500          -------------------------------------------------------NETAS
TcIL3000016620          -------------------------------------------------------NETES
TcIL3000016630          ------------------------------------------------------------
TcIL3000047260          --------------------------------------------------NGTDTNETAS

Tbg97291330             DENTTADENTTADGNTNADENTTADEN---------------------------------
Tbg97291280             -EN------TTADGNTNADEN---------------------------------------
Tbg97291270             DENTTADENTTADGNTTADENTTVDEN---------------------------------
Tbg97291300             DENTTADGNTTADGNTNADENTTVDEN---------------------------------
Tb09v40146              DENTTADENTTADENTTADENTTVDEN---------------------------------
Tb09v40147              DENTTVDENTTADENTTADENTTADEN---------------------------------
Tb091601520             DENATVDENTTVDENTTADENTTADEN---------------------------------
Tb09v40149              ---------TTVDGNTNADENTTADEN---------------------------------
Tb09v40059              ---------TTVDENTNADENTTADEN---------------------------------
Tb09v40058              ---------ATVDENTTADENTTVDEN---------------------------------
Tb09v40148              DENTTVDENTTADGNTNADENTTADEN---------------------------------
Tbg97291380             DGNTTADGNTTADGNTNADGNTTTDEN---------------------------------
Tb09v40060              HENRSYDDSSVADGNTSDDKNTTSHENRSYDDSSVADGNTSEDKNTTSHENRSYDDSSVA
Tbg97291360             HENRSCDDSSVADGNTSDDKNTTSHENRSYDDSSVADGNTSDDKNTTSHENRSYDDSSVA
Tc00104705351105310     ----------TPEKTFG-------------------------------------------
Tc00104705350763120     ----------TPENTFG-------------------------------------------
Tc00104705350401180     ----------TPEKTFG-------------------------------------------
Tc001047053511859200    ----------TPEDTFG-------------------------------------------
Tc00104705350601110     ----------TPEDTFG-------------------------------------------
Tc00104705351104770     ----------TPENTFG-------------------------------------------
Tc00104705350453350     ----------TPYRSFG-------------------------------------------
Tc001047053508735100    ----------TPYRSFG-------------------------------------------
Tc0010475350894510      ----------TPYRSFG-------------------------------------------
Tc00104705350863740     ----------TPYLSFG-------------------------------------------
Tc00104705351112960     ----------TPYRSFG-------------------------------------------
Tc00104705351191190     ----------TPYRSFG-------------------------------------------
Tc00104705351024930     ----------TPYRSFG-------------------------------------------
Tc00104705350735910     ----------TPYRSFG-------------------------------------------
TvY486_0201870          --TTTTKPPTTPTPTTT----TTTTTTKP-------------------------------
TvY486_0600050          -----TKPPTTPTPTTT----TTTTTTKP-------------------------------
```

```
TvY486_0028480          --GNILIPNSSPSSSTNNAPVSSTLTAMPTVDEASLAASGGGSNGTNGA-----------
TvY486_0201850          --TSTTTTTKAPTSTTT----STTATVAPTNTTSSSSTTESPSSPTTMADG---------
Tbg97223580             ---------TTAAPASGVVPEP--------------------------------------
Tbg97223450             ---------TTAAPASGVVPEP--------------------------------------
Tbg97223390             ---------TTAAPASGVVPEP--------------------------------------
Tbg97223430             ---------TTAAPASGVVPEP--------------------------------------
Tbg97223760             ---------TTAAPASGVVPEP--------------------------------------
Tbg97223550             ---------TTAAPASGVVPEP--------------------------------------
Tbg97223410             ---------TTAAPASGVVPEP--------------------------------------
Tbg97223680             ---------TTAAPASGVVPEP--------------------------------------
Tbg97223740             ---------TTAAPASGVVPEP--------------------------------------
Tbg97223540             ---------TTAAPASGVVPEP--------------------------------------
Tb92725360              ---------TTAAPASGEVPEP--------------------------------------
Tb92725340              ---------TTAAPASGEVPEP--------------------------------------
Tb92725330              ---------TTAAPASGEVPEP--------------------------------------
Tbg97223600             ---------TTAAPASGEVPEP--------------------------------------
Tbg97223520             ---------TTAAPASGEVPEP--------------------------------------
Tbg97223370             ---------TTAAPASGEVPEP--------------------------------------
Tbg97223470             ---------TTAAPASGEVPEP--------------------------------------
Tbg97223700             ---------TTAAPASGEVPEP--------------------------------------
Tbg97223620             ---------TTAAPASGEVPEP--------------------------------------
Tbg97223490             ---------TTAAPASGEVPEP--------------------------------------
Tbg97223660             ---------TTAAPASGEVPEP--------------------------------------
Tbg97223640             ---------TTAAPASGEVPEP--------------------------------------
Tb92725350              ---------TTAAPASGEVPEP--------------------------------------
Tbg97223330             ---------TTAAPASGVVPEP--------------------------------------
Tb92725290              ---------TTAAPASGEVPEP--------------------------------------
Tbg97223720             ---------TTAAPASGEVPEP--------------------------------------
Tb92725320              ---------TTAAPASGEVPEP--------------------------------------
Tbg97223350             ---------TTAAPASGEVPEP--------------------------------------
Tbg97223500             ---------TTAAPASGEVPEP--------------------------------------
Tb92725300              ---------TTAAPASGEVPEP--------------------------------------
Tb92725310              ---------TTAAPASGEVPEP--------------------------------------
Tbg97222800             -----------AAPASGEVPEP--------------------------------------
Tb92724760              -----------TAPASGEVPEP--------------------------------------
Tbg97222940             -----------AAPASGEVPEP--------------------------------------
Tb92724920              -----------AAPASGEVPEP--------------------------------------
TcIL30002110            ---------TTTTTTTTKKPENTTTSTTT-------------------------------
TcIL3000013740          ---------TTTTTTTTKKPENTTTSTTT-------------------------------
TcIL3000027940          ---------TTTTTTTTKKPENTTTSTTT-------------------------------
TcIL3000001110          ---------TPTTTTTTKQPEN--------------------------------------
TcIL3000033420          ---------TTTTTTTTKQPEN--------------------------------------
Tb9276380               ----------TANEELAVINESLHSDEE--------------------------------
Tbg972630               EDEVSGNGNSTANEELAVINESLHSDEE--------------------------------
Tb9276370               ----------TANEELAVINESLHSDEE--------------------------------
TcIL3000050510          EELNGTNTNETASEGLNGTNANETG-----------------------------------
TcIL3000050500          EELNGTNANETASEELNGTNANETASEELNGTNANETESEELNGTSANETESEELN----
TcIL3000016620          EELNGTSANETASEELNGTNVNETE-----------------------------------
TcIL3000016630          --LSGPEGSETASEELNGTNVNETE-----------------------------------
TcIL3000047260          EELNGTDANETASEELNGTNANETASEELNGTDANETASEELNGTDANETASEELNGTDA


Tbg97291330             ---------TTADENTTVDGD------------TNAD-----------------------
Tbg97291280             ------------------------------------------------------------
Tbg97291270             ------------------------------------------------------------
Tbg97291300             ---------TTVDEN---------------------------------------------
Tb09v40146              ---------TTVDENTTVDENTTADGNTNADENTTAD-----------------------
Tb09v40147              ---------TTVDENTTADEN------------TTVD-----------------------
Tb091601520             ---------TTADEN---------------------------------------------
Tb09v40149              ---------TTADEN---------------------------------------------
Tb09v40059              ---------TTVDENTNADEN------------TTAD-----------------------
Tb09v40058              ---------TTVDEN---------------------------------------------
Tb09v40148              ---------TTVDEN------------------TTVD-----------------------
Tbg97291380             ---------TTADENTNADGNTTTDGNTNADGNTTADGNITTDGNTNADGNTTADGNTTA
Tb09v40060              DGNTSDDKNTTSHENSSHDDSSVADGNTSEDKNTTSHENRSHDDSSVADGNTSEDKNTTS
Tbg97291360             DGNSSDDKNTTSHENRSYDDSSVADGNSSDDKNTTSHENRSYDDSSVADGNTSDDKNTTT
Tc00104705351105310     ------------------------------------------------------------
Tc00104705350763120     ------------------------------------------------------------
Tc00104705350401180     ------------------------------------------------------------
Tc001047053511859200    ------------------------------------------------------------
Tc00104705350601110     ------------------------------------------------------------
Tc00104705351104770     ------------------------------------------------------------
Tc00104705350453350     ------------------------------------------------------------
Tc001047053508735100    ------------------------------------------------------------
Tc0010475350894510      ------------------------------------------------------------
Tc00104705350863740     ------------------------------------------------------------
Tc00104705351112960     ------------------------------------------------------------
Tc00104705351191190     ------------------------------------------------------------
Tc00104705351024930     ------------------------------------------------------------
```

```
Tc00104705350735910        ------------------------------------------------------------
TvY486_0201870             ------------------------------------------------------------
TvY486_0600050             ------------------------------------------------------------
TvY486_0028480             ------------------------------------------------------------
TvY486_0201850             ------------------------------------------------------------
Tbg97223580                ------------------------------------------------------------
Tbg97223450                ------------------------------------------------------------
Tbg97223390                ------------------------------------------------------------
Tbg97223430                ------------------------------------------------------------
Tbg97223760                ------------------------------------------------------------
Tbg97223550                ------------------------------------------------------------
Tbg97223410                ------------------------------------------------------------
Tbg97223680                ------------------------------------------------------------
Tbg97223740                ------------------------------------------------------------
Tbg97223540                ------------------------------------------------------------
Tb92725360                 ------------------------------------------------------------
Tb92725340                 ------------------------------------------------------------
Tb92725330                 ------------------------------------------------------------
Tbg97223600                ------------------------------------------------------------
Tbg97223520                ------------------------------------------------------------
Tbg97223370                ------------------------------------------------------------
Tbg97223470                ------------------------------------------------------------
Tbg97223700                ------------------------------------------------------------
Tbg97223620                ------------------------------------------------------------
Tbg97223490                ------------------------------------------------------------
Tbg97223660                ------------------------------------------------------------
Tbg97223640                ------------------------------------------------------------
Tb92725350                 ------------------------------------------------------------
Tbg97223330                ------------------------------------------------------------
Tb92725290                 ------------------------------------------------------------
Tbg97223720                ------------------------------------------------------------
Tb92725320                 ------------------------------------------------------------
Tbg97223350                ------------------------------------------------------------
Tbg97223500                ------------------------------------------------------------
Tb92725300                 ------------------------------------------------------------
Tb92725310                 ------------------------------------------------------------
Tbg97222800                ------------------------------------------------------------
Tb92724760                 ------------------------------------------------------------
Tbg97222940                ------------------------------------------------------------
Tb92724920                 ------------------------------------------------------------
TcIL30002110               ------------------------------------------------------------
TcIL3000013740             ------------------------------------------------------------
TcIL3000027940             ------------------------------------------------------------
TcIL3000001110             ------------------------------------------------------------
TcIL3000033420             ------------------------------------------------------------
Tb9276380                  ------------------------------------------------------------
Tbg972630                  ------------------------------------------------------------
Tb9276370                  ------------------------------------------------------------
TcIL3000050510             ------------------------------------------------------------
TcIL3000050500             ------------------------------------------------------------
TcIL3000016620             ------------------------------------------------------------
TcIL3000016630             ------------------------------------------------------------
TcIL3000047260             NETASEELNGTDANETASEELNGTDANETASEELNGTDANETASEELNGTDANETASEEL

Tbg97291330                ------------------------------------------------------------
Tbg97291280                ------------------------------------------------------------
Tbg97291270                ------------------------------------------------------------
Tbg97291300                ------------------------------------------------------------
Tb09v40146                 ------------------------------------------------------------
Tb09v40147                 ------------------------------------------------------------
Tb091601520                ------------------------------------------------------------
Tb09v40149                 ------------------------------------------------------------
Tb09v40059                 ------------------------------------------------------------
Tb09v40058                 ------------------------------------------------------------
Tb09v40148                 ------------------------------------------------------------
Tbg97291380                DGNTNADGNTTTD-----------------------------------------------
Tb09v40060                 HENSSHDDSSVADGNTSEDKNTTSHENRSYDDSSVADGNTSEDKNTTSHENSSHDDSSVA
Tbg97291360                HENRSYDDSSVADGNTSDDKNTTSHENRSYDDSSVADGNTSDDKNTTIHENRSYDDSSVA
Tc00104705351105310        ------------------------------------------------------------
Tc00104705350763120        ------------------------------------------------------------
Tc00104705350401180        ------------------------------------------------------------
Tc001047053511859200       ------------------------------------------------------------
Tc00104705350601110        ------------------------------------------------------------
Tc00104705351104770        ------------------------------------------------------------
Tc00104705350453350        ------------------------------------------------------------
Tc001047053508735100       ------------------------------------------------------------
Tc0010475350894510         ------------------------------------------------------------
Tc00104705350863740        ------------------------------------------------------------
```

```
Tc00104705351112960       ------------------------------------------------------------
Tc00104705351191190       ------------------------------------------------------------
Tc00104705351024930       ------------------------------------------------------------
Tc00104705350735910       ------------------------------------------------------------
TvY486_0201870            ------------------------------------------------------------
TvY486_0600050            ------------------------------------------------------------
TvY486_0028480            ------------------------------------------------------------
TvY486_0201850            ------------------------------------------------------------
Tbg97223580               ------------------------------------------------------------
Tbg97223450               ------------------------------------------------------------
Tbg97223390               ------------------------------------------------------------
Tbg97223430               ------------------------------------------------------------
Tbg97223760               ------------------------------------------------------------
Tbg97223550               ------------------------------------------------------------
Tbg97223410               ------------------------------------------------------------
Tbg97223680               ------------------------------------------------------------
Tbg97223740               ------------------------------------------------------------
Tbg97223540               ------------------------------------------------------------
Tb92725360                ------------------------------------------------------------
Tb92725340                ------------------------------------------------------------
Tb92725330                ------------------------------------------------------------
Tbg97223600               ------------------------------------------------------------
Tbg97223520               ------------------------------------------------------------
Tbg97223370               ------------------------------------------------------------
Tbg97223470               ------------------------------------------------------------
Tbg97223700               ------------------------------------------------------------
Tbg97223620               ------------------------------------------------------------
Tbg97223490               ------------------------------------------------------------
Tbg97223660               ------------------------------------------------------------
Tbg97223640               ------------------------------------------------------------
Tb92725350                ------------------------------------------------------------
Tbg97223330               ------------------------------------------------------------
Tb92725290                ------------------------------------------------------------
Tbg97223720               ------------------------------------------------------------
Tb92725320                ------------------------------------------------------------
Tbg97223350               ------------------------------------------------------------
Tbg97223500               ------------------------------------------------------------
Tb92725300                ------------------------------------------------------------
Tb92725310                ------------------------------------------------------------
Tbg97222800               ------------------------------------------------------------
Tb92724760                ------------------------------------------------------------
Tbg97222940               ------------------------------------------------------------
Tb92724920                ------------------------------------------------------------
TcIL30002110              ------------------------------------------------------------
TcIL3000013740            ------------------------------------------------------------
TcIL3000027940            ------------------------------------------------------------
TcIL3000001110            ------------------------------------------------------------
TcIL3000033420            ------------------------------------------------------------
Tb9276380                 ------------------------------------------------------------
Tbg972630                 ------------------------------------------------------------
Tb9276370                 ------------------------------------------------------------
TcIL3000050510            ------------------------------------------------------------
TcIL3000050500            ------------------------------------------------------------
TcIL3000016620            ------------------------------------------------------------
TcIL3000016630            ------------------------------------------------------------
TcIL3000047260            NGTDANETASEELNGTDANETASEELNGTDANETASEELNGTDANETASEELNGTDANET

Tbg97291330               ------------------------------------------------------------
Tbg97291280               ------------------------------------------------------------
Tbg97291270               ------------------------------------------------------------
Tbg97291300               ------------------------------------------------------------
Tb09v40146                ------------------------------------------------------------
Tb09v40147                ------------------------------------------------------------
Tb091601520               ------------------------------------------------------------
Tb09v40149                ------------------------------------------------------------
Tb09v40059                ------------------------------------------------------------
Tb09v40058                ------------------------------------------------------------
Tb09v40148                ------------------------------------------------------------
Tbg97291380               ------------------------------------------------------------
Tb09v40060                DGNTSEDKNTTSHENRSYDDSSVADGNTSEDKNTTSHENRSYGDTSVADLSSSDDESTSD
Tbg97291360               DGNTSDDKNTTSHENRSYDDSSVADGNTSDDKNTTSHENRSCDDSSVADGNTSDDESTSD
Tc00104705351105310       ------------------------------------------------------------
Tc00104705350763120       ------------------------------------------------------------
Tc00104705350401180       ------------------------------------------------------------
Tc001047053511859200      ------------------------------------------------------------
Tc00104705350601110       ------------------------------------------------------------
Tc00104705351104770       ------------------------------------------------------------
Tc00104705350453350       ------------------------------------------------------------
```

```
Tc001047053508735100      ------------------------------------------------------------
Tc0010475350894510        ------------------------------------------------------------
Tc00104705350863740       ------------------------------------------------------------
Tc00104705351112960       ------------------------------------------------------------
Tc00104705351191190       ------------------------------------------------------------
Tc00104705351024930       ------------------------------------------------------------
Tc00104705350735910       ------------------------------------------------------------
TvY486_0201870            ------------------------------------------------------------
TvY486_0600050            ------------------------------------------------------------
TvY486_0028480            ------------------------------------------------------------
TvY486_0201850            ------------------------------------------------------------
Tbg97223580               ------------------------------------------------------------
Tbg97223450               ------------------------------------------------------------
Tbg97223390               ------------------------------------------------------------
Tbg97223430               ------------------------------------------------------------
Tbg97223760               ------------------------------------------------------------
Tbg97223550               ------------------------------------------------------------
Tbg97223410               ------------------------------------------------------------
Tbg97223680               ------------------------------------------------------------
Tbg97223740               ------------------------------------------------------------
Tbg97223540               ------------------------------------------------------------
Tb92725360                ------------------------------------------------------------
Tb92725340                ------------------------------------------------------------
Tb92725330                ------------------------------------------------------------
Tbg97223600               ------------------------------------------------------------
Tbg97223520               ------------------------------------------------------------
Tbg97223370               ------------------------------------------------------------
Tbg97223470               ------------------------------------------------------------
Tbg97223700               ------------------------------------------------------------
Tbg97223620               ------------------------------------------------------------
Tbg97223490               ------------------------------------------------------------
Tbg97223660               ------------------------------------------------------------
Tbg97223640               ------------------------------------------------------------
Tb92725350                ------------------------------------------------------------
Tbg97223330               ------------------------------------------------------------
Tb92725290                ------------------------------------------------------------
Tbg97223720               ------------------------------------------------------------
Tb92725320                ------------------------------------------------------------
Tbg97223350               ------------------------------------------------------------
Tbg97223500               ------------------------------------------------------------
Tb92725300                ------------------------------------------------------------
Tb92725310                ------------------------------------------------------------
Tbg97222800               ------------------------------------------------------------
Tb92724760                ------------------------------------------------------------
Tbg97222940               ------------------------------------------------------------
Tb92724920                ------------------------------------------------------------
TcIL30002110              ------------------------------------------------------------
TcIL3000013740            ------------------------------------------------------------
TcIL3000027940            ------------------------------------------------------------
TcIL3000001110            ------------------------------------------------------------
TcIL3000033420            ------------------------------------------------------------
Tb9276380                 ------------------------------------------------------------
Tbg972630                 ------------------------------------------------------------
Tb9276370                 ------------------------------------------------------------
TcIL3000050510            ------------------------------------------------------------
TcIL3000050500            ------------------------------------------------------------
TcIL3000016620            ------------------------------------------------------------
TcIL3000016630            ------------------------------------------------------------
TcIL3000047260            ASEELNGTDANETASEELNGTDANETASEELNGTDTNETASEELNGTDANETASEELNGT
```

```
Tbg97291330               ----------------------------------------ENTTVDENTTVDEN------
Tbg97291280               ------------------------------------------------TTVDEN------
Tbg97291270               ------------------------------------------TTVDENTTVDEN------
Tbg97291300               ------------------------------------------TTVDENTTVDEN------
Tb09v40146                ----------------------------------------ENTTVDENTTVDEN------
Tb09v40147                ----------------------------------------ENTTVDENTTVDEN------
Tb091601520               ------------------------------------------TTADENATADEN------
Tb09v40149                ------------------------------------------TTADENTTADEN------
Tb09v40059                ----------------------------------------ENTNVDENTTADEN------
Tb09v40058                ------------------------------------------TNVDENTTADEN------
Tb09v40148                ----------------------------------------ENTTVDENTTVDEN------
Tbg97291380               ----------------------------ENTTADENTNADGNTTVDENTTADGN------
Tb09v40060                DESTSDYESTADEEYSLYNN----GIWKENTTADENTTADENTNVDENTTADEN------
Tbg97291360               DESTSDYESTADEEYSLYNN----GIWKENTTADENTTVDENTTVDENTTVDENTTVDEN
Tc00104705351105310       --------------------------------------------VVCETQEGYV------
Tc00104705350763120       --------------------------------------------VVCETQEGYV------
Tc00104705350401180       --------------------------------------------VVCETQEGYV------
Tc001047053511859200      --------------------------------------------VVCETQEGYV------
```

```
Tc00104705350601110       --------------------------------------------VVCETQEGYV------
Tc00104705351104770       --------------------------------------------VVCETQEGYV------
Tc00104705350453350       --------------------------------------------VVCETQKGYV------
Tc001047053508735100      --------------------------------------------VVCETQKGYV------
Tc0010475350894510        --------------------------------------------VVCEAQKGYV------
Tc00104705350863740       --------------------------------------------VVCEAQKGYV------
Tc00104705351112960       --------------------------------------------VVCEAQKGYV------
Tc00104705351191190       --------------------------------------------VVCEAQKGYV------
Tc00104705351024930       --------------------------------------------VVCEAQKGYV------
Tc00104705350735910       --------------------------------------------VVCEAQKGYV------
TvY486_0201870            ---------------------------PT----KTTTTT-----TSAPDVSEWV------
TvY486_0600050            ---------------------------PT----TTTTTT-----TSAPDVSEWV------
TvY486_0028480            ------------------------GHHPTGSAVTAVTATKPPPPTSAPDVSEWV------
TvY486_0201850            ------------------------SSGPSGGAAGTPSSS--SG-TASATSPGPV------
Tbg97223580               -------------------------------------------TVTKDEEVT--------
Tbg97223450               -------------------------------------------TVTKDEEVT--------
Tbg97223390               -------------------------------------------TVTKDEEVT--------
Tbg97223430               -------------------------------------------TVTKDEEVT--------
Tbg97223760               -------------------------------------------TVTKDEEVT--------
Tbg97223550               -------------------------------------------TVTKDEEVT--------
Tbg97223410               -------------------------------------------TVTKDEEVT--------
Tbg97223680               -------------------------------------------TVTKDEEVT--------
Tbg97223740               -------------------------------------------TVTKDEEVT--------
Tbg97223540               -------------------------------------------TVTKDEEVT--------
Tb92725360                -------------------------------------------TVTKDEEVT--------
Tb92725340                -------------------------------------------TVTKDEEVT--------
Tb92725330                -------------------------------------------TVTKDEEVT--------
Tbg97223600               -------------------------------------------TVTKDEEVT--------
Tbg97223520               -------------------------------------------TVTKDEEVT--------
Tbg97223370               -------------------------------------------TVTKDEEVT--------
Tbg97223470               -------------------------------------------TVTKDEEVT--------
Tbg97223700               -------------------------------------------TVTKDEEVT--------
Tbg97223620               -------------------------------------------TVTKDEEVT--------
Tbg97223490               -------------------------------------------TVTKDEEVT--------
Tbg97223660               -------------------------------------------TVTKDEEVT--------
Tbg97223640               -------------------------------------------TVTKDEEVT--------
Tb92725350                -------------------------------------------TVTKDEEVT--------
Tbg97223330               -------------------------------------------TVTKDEEVT--------
Tb92725290                -------------------------------------------TVTKDEEVT--------
Tbg97223720               -------------------------------------------TVTKDEEVT--------
Tb92725320                -------------------------------------------TVTKDEEVT--------
Tbg97223350               -------------------------------------------TVTKDEEVT--------
Tbg97223500               -------------------------------------------TVTKDEEVT--------
Tb92725300                -------------------------------------------TVTKDEEVT--------
Tb92725310                -------------------------------------------TVTKDEEVT--------
Tbg97222800               -------------------------------------------TVTKDEEVT--------
Tb92724760                -------------------------------------------TVTKDEEVT--------
Tbg97222940               -------------------------------------------TVTKDEEVT--------
Tb92724920                -------------------------------------------TVTKDEEVT--------
TcIL30002110              -----------------------------------------TTTTTAEPENT-T------
TcIL3000013740            -----------------------------------------TTTTTAEPENT-T------
TcIL3000027940            -----------------------------------------TTTTTAEPENT-T------
TcIL3000001110            -----------------------------------------TTTTTKEPENN-T------
TcIL3000033420            -----------------------------------------TTTTTKEPENTTT------
Tb9276380                 ---------------------------------------------STNSQDQ--------
Tbg972630                 ---------------------------------------------STNSQDQ--------
Tb9276370                 ---------------------------------------------STNSQDQ--------
TcIL3000050510            ---------------------------------------------SEDSSSG--------
TcIL3000050500            ------------------------GTNANETASEELNGTNANETESDDSSSG--------
TcIL3000016620            ---------------------------------------------SDDSSSG--------
TcIL3000016630            ---------------------------------------------SDDSSSG--------
TcIL3000047260            NANETASEELNGTDTNETASEELNGTNANETASEELNGTDANETASEDSPSG--------


Tbg97291330               TNTDEISNGSNEASDKTVPSTASDGEGSGGGAGAAVVIIFILLVVLLVLLYFCCFAG--H
Tbg97291280               TNTDEISNGSNEASDKTVPSTSSDGEGSGGGAGAAVVIIFILLVVLLVLLYFCCFAG--H
Tbg97291270               TNTDEISNGSNEASDKTVPSTASDGEGSGGGAGAAVVIIFILLVVLLVLLYFCCFAG--H
Tbg97291300               TNTDEISNGSNEASDKTVPSTASDGEGSGGGAGAAVVIIFILLVVLLVLLYFCCFAG--H
Tb09v40146                TNADEISNGSNEASDKTVPSTASDGEGSGGGAGAAVVIIFILLAVLLILLYFCCFAG--H
Tb09v40147                TNADEISNGSNEASDKTVPSTASDGEGSGGGAGAAVVIIFILLAVLLILLYFCCFAG--H
Tb091601520               TIADEISNGSNEASDKTVPSTASDGEGSGGGAGAAVVIIFILLAVLLILLYFCCFAG--H
Tb09v40149                TNADEISNGSNEASDKTVPSTASDGEGSGGGAGAAVVIIFILLAVLLILLYFCCFAG--H
Tb09v40059                TIADEISNGSNEASDKTVPSTASDGEGSGGGAGAAVVIIFILLAVLLILLYFCCFAG--H
Tb09v40058                TIADEISNGSNEASDKTVPSTASDGEGSGGGAGAAVVIIFILLAVLLILLYFCCFAG--H
Tb09v40148                TNADEISNGSNEASDKTVPSTASDGEGSGGGAGAAVVIIFILLAVLLILLYFCCFAG--H
Tbg97291380               TTADEISNGSNEASDKTVPSTASDGEGSGGGAGAAVVIIFILLVVLLVLLYFCCFAG--H
Tb09v40060                TNADEISNGSNEASDKTVPSTASDGEGSGGGAGAAVVIIFILLAVLLILLYFCCFAG--H
Tbg97291360               TNTDEISNGSNEASDKTVPSTSSDGEGSGGGAGAAVVIIFILLVVLLVLLYFCCFAG--H
Tc00104705351105310       VSTTTLPPT----------VEISWVHKRWYVILIAILVPVIVAVAIITVCFCRSCGGHDE
```

```
Tc00104705350763120         VSTTTLPPT----------VEIPWVHKRWYVILIAILVPVIVAVAIITVYFCRSCGGHDE
Tc00104705350401180         VSTTTLPPT----------VEIPWVHKRWYVILIAILVPVIVAVAIITACFCRCRGADDE
Tc001047053511859200        VSTTTLPPT----------VEISWVRKNWYFVLIAILVPVIVAVALITVCFCRCRGADDE
Tc00104705350601110         VSTTTLPPT----------VKISWVHKRWYVILIAILVPVIVAVTLITVCFCRCRGADDE
Tc00104705351104770         VSTTTLPPA----------VEISWVQKNWYFVLAAILVPVIVAVVLITACFCRCRGADDE
Tc00104705350453350         VSTTTLPPA----------IVVPWLQKNWYVVLIVVLLPVVVAVALITVCFYRCRGVDDE
Tc001047053508735100        VSTTTLPPA----------VVVPWLQKNWYVVLIVVLLPVVVAVALITVCFYRCRGVDDE
Tc0010475350894510          VSTTTLPPA----------VVVPWLQKNWYVVLLAVLLPVIAAAALITVCFYRCRGV-DE
Tc00104705350863740         VSTTTLPPA----------VVVPWLQKNWYFVLLAVLLPVIAAAALITVCFYRCRGVYDE
Tc00104705351112960         VSTTTLPPA----------VVVPWLQKNWYVVLMAVLLPVIAAVALITVCFYRCRGVEDE
Tc00104705351191190         VSTTTLPPA----------VVVPWLQKNWYVVLIAVLLPVIAAVALITVCFYRCRGVDDE
Tc00104705351024930         VSTTTLPPA----------VVVPWLQKNWYVVLIAVLLPVIAAVALITVCFYRCRGVDDE
Tc00104705350735910         VSTTTLPPA----------VVVPWLQKNWYFILAAILLPVIAAVALITVCFCRCRGVDDE
TvY486_0201870              SSATFVPPVPGAKGQVGRDASSTWAERYWYVILLGLLVAIAAVVLIVLCLCLRCCAG--D
TvY486_0600050              SSATFVPPVPGAKGQVGRDASSTWAERYWYVILLGLLVAIAAVVLIVLCLCLRCCAG--D
TvY486_0028480              SSATFVPPVPGAKGQVGRDASSTWAERYWYVILLSLLVAIAAVVLIVLCLCLRCCAG--D
TvY486_0201850              GNATSAPPVPGGAGHAGLGASSTWAERYWYVILLVFLSVIVAIVLIVLLFVCRSRSR--D
Tbg97223580                 VTTASLEERS-----------------NWHIILIALISVVSLIVLLLFLLRWFCDYN--V
Tbg97223450                 VTTASLEERS-----------------NWHIILIALISVVSLIVLLLFLLRWFCDYN---
Tbg97223390                 VTTASLEERS-----------------NWHIILIALISVVSLIVLLLFLLRWFCDYN--V
Tbg97223430                 VTTASLEERS-----------------NWHIILIALISVVSLIVLLLFLLRWFCDYN--V
Tbg97223760                 VTTASLEERS-----------------NWHIILIALISVVSLIVLLLFLLRWFCDYN--V
Tbg97223550                 VTTASLEERS-----------------NWHIILIALISVVSLIVLLLFLLRWFCDYN--V
Tbg97223410                 VTTASLEERS-----------------NWHIILIALISVVSLIVLLLFLLRWFCDYN--V
Tbg97223680                 VTTASLEERS-----------------NWHIILIALISVVSLIVLLLFLLRWFCDYN--V
Tbg97223740                 VTTASLEERS-----------------NWHIILIALISVVSLIVLLLFLLRWFCDYN--V
Tbg97223540                 VTTASLEERS-----------------NWHIILIALISVVSLIVLLLFLLRWFCDYN--V
Tb92725360                  VTTVSLEERS-----------------NWHIILIALISVVSLIVLLLFLLRWFCDYD--V
Tb92725340                  VTTVSLEERS-----------------NWHIILIALISVVSLIALLLFLLRWFCDYD--V
Tb92725330                  VTTVSLEERS-----------------NWHIILIALISVVSLIVLLLFLLRWFCDYD--V
Tbg97223600                 VTTVSLEERS-----------------NWHIILIALISVVALIVLLLFLLRWFCDYN--V
Tbg97223520                 VTTVSLEERS-----------------NWHIILIALISVVALIVLLLFLLRWFCDYN--V
Tbg97223370                 VTTVSLEERS-----------------NWHIILIALISVVALIVLLLFLLRWFWDYN--V
Tbg97223470                 VTTVSLEERS-----------------NWHIILIALISVVALIVLLLFLLRWFCDYN--V
Tbg97223700                 VTTVSLEERS-----------------NWHIILIALISVVALIVLLLFLLRWFCDYN--V
Tbg97223620                 VTTVSLEERS-----------------NWHIILIALISVVALIVLLLFLLRWFCDYN--V
Tbg97223490                 VTTVSLEERS-----------------NWHIILIALISVVALIVLLLFLLRWFCDYN--V
Tbg97223660                 VTTVSLEERS-----------------NWHIILIALISVVSLIVLLLFLLRWFCDYN--V
Tbg97223640                 VTTVSLEERS-----------------NWHIILIALISVVALIVLLLFLLRWFCDYN--V
Tb92725350                  VTTVSLEERS-----------------NWHIILIALISVVSLIVLLLFLLRWFCDYD--V
Tbg97223330                 VTTVSLEERS-----------------NWHIILIALISVVALIVLLLLLLRWFCDYN--V
Tb92725290                  VTTVSLEERS-----------------NWHIILIALISVVSLIVLLLFLLRWFCDYD--V
Tbg97223720                 VTTVSLEERS-----------------NWHIILIALISVVSLIVLLLFLLRWFCDYN--V
Tb92725320                  VTTVSLEERS-----------------NWHIILIALISVVSLIVLLLFLLRWFCDYD--V
Tbg97223350                 VTTVSLEERS-----------------NWHIILIALISVVSLIVLLLFLLRWFCDYN--V
Tbg97223500                 VTTVSLEERS-----------------NWHIILIALISVVSLIVLLLFLLRWFCDYN--V
Tb92725300                  VTTVSLEERS-----------------NWHIILIALISVVSLIVLLLFLLRWFCDYD--V
Tb92725310                  VTTVSLEERS-----------------NWHIILIALISVVSLIVLLLFLLRWFCDYD--V
Tbg97222800                 VTTASLEERI-----------------DLLIIILIALISVVSLIVLLLFLLRWFCDYN--V
Tb92724760                  VTTVSLEERI-----------------DLLIIILIALISVVSLIVLLLFLLRWFCDYN--V
Tbg97222940                 VTTASLEERS-----------------NWHIILIALISVVALIVLLLFLLRWFCDYN--V
Tb92724920                  VTTASLEERS-----------------NWHIILIALISVVSLIVLLLFLLRWFCDYN--V
TcIL30002110                TTTTAQPEASEGK-GSG------------YIILIVLLLLAALIALLLLAYCCFSAG---G
TcIL3000013740              TTTTAQPEASEGK-GSG------------YIILIVLLLLAALIALLLLAYCCFSAG---G
TcIL3000027940              TTTTAQPEASEGK-GSG------------YIILIVLLLLAALIALLLLAYCCFSAG---G
TcIL3000001110              TTTTAQPEASEGSDGSSG-ASTSWGTRNWYIILIVLLLLAALIALLLLAYCCFSAG---G
TcIL3000033420              TTTTAQPEASEGSDGSSG-ASASWGTRNWYIILIVFLLLAALIALLLLAYCCFSAG---G
Tb9276380                   PSATPKPKGE----------------AETTLTSLSHITICVGGELCLLIVFFSLAIV--C
Tbg972630                   PSATPKPKGE----------------AETTLTSLSHITICVGGELCLLIVFFSLAIV--C
Tb9276370                   PSATPKPKGE----------------AETTLTSLSHITICVGGELCLLIVFFSLAIV--C
TcIL3000050510              VSSTEAPAGK----------------VNGGVLAVAILLPIIAIALLLLLWYFCFRRR--D
TcIL3000050500              VSSTEAPAGK----------------VNGGVLAVAILLPIIAIALLLLLWYFCFRRR--D
TcIL3000016620              VSSTEAPAGK----------------VNGGVLALAILLPIIAIALLLLLWYFCFRRR--D
TcIL3000016630              VSSTEAPAGK----------------VNGGVLAVAILLPIIAIALLLLLWYFCFRRR--D
TcIL3000047260              VSSTAAPAGK----------------VNGGVLALAILLPIIAIVLLLLAWYFCVGWK--N
                              :                                   :         :

Tbg97291330                 EKYITVMSLREKVTSP---VSNVEAAEVAAVP-----------SNGEEHLSITSHQQETP
Tbg97291280                 EKYITVMSLREKVTSP---VSNVEAAEVAAVP-----------SNGEEHLSITSHQQETP
Tbg97291270                 EKYITVMSLREKVTSP---VSNVEAAEVAAVP-----------SNGEEHLSITSHQQETP
Tbg97291300                 EKYITVMSLREKVTSP---VSNVEAAEVAAVP-----------SNGEEHLSITSHQQETP
Tb09v40146                  EKYITVMSLREKVTSP---VSNVEAAEVAAVP-----------SNGEEHLSITSHQQETP
Tb09v40147                  EKYITVMSLREKVTSP---VSNVEAAEVAAVP-----------SNGEEHLSITSHQQETP
Tb091601520                 EKYITVMSLREKVTSP---VSNVEAAEVAAVP-----------SNGEEHLSITSHQQETP
Tb09v40149                  EKYITVMSLREKVTSP---VSNVEAAEVAAVP-----------SNGEEHLSITSHQQETP
Tb09v40059                  EKYITVMSLREKVTSP---VSNVEAAEVAAVP-----------SNGEEHLSITSHQQETP
Tb09v40058                  EKYITVMSLREKVTSP---VSNVEAAEVAAVP-----------SNGEEHLSITSHQQETP
Tb09v40148                  EKYITVMSLREKVTSP---VSNVEAAEVAAVP-----------SNGEEHLSITSHQQETP
Tbg97291380                 EKYITVMSLREKVTSP---VSNVEAAEVAAVP-----------SNGEEHLSITSHQQETP
```

Tb09v40060	EKYITVMSLREKVTSP---VSNVEAAEVAAVP-----------SNGEEHLSITNHQQETP
Tbg97291360	EKYITVMSLREKVTSP---VSNVEAAEVAAVP-----------SNGEEHLSITSHQQETP
Tc00104705351105310	SKWVVHMVLREVNGEP---LHPIDGDGGIYND-DSPMMRYSTLGDGVPMTAPSGYENVEV
Tc00104705350763120	SKWVVHMVLREVNGEP---LHPIDGDGGIYND-DSPMMRYSTLGDGVPMTAPSGYENVEV
Tc00104705350401180	SKWVVHMVLREVNSEP---LHPIDGDGGIYND-DSQMMRYSTLGDGVPMTAPSGYENVEV
Tc00104705511859200	SKWVVHMVLREVNGKP---LHPIDGDDGIYND-NSPMMRYSTLGDGVPMNTPFAYENVEV
Tc00104705350601110	SKWVVHMVLREVSSEP---LHPIDGDDGIYND-NSPMMRYSTLGDGVPMNTPFAYENVEV
Tc00104705351104770	SKWVVHMVLREVNGKP---FHVTDRNGGIYND-DSPMMRYSTLGDGVPMTAPFAYENVEV
Tc00104705350453350	SKWVVHMVLREINDNP---LYAIDRNDGIYNN-DGQMKQDSTLGDGMPTTTPSWHENAEV
Tc001047053508735100	SKWVAHMVLREINDNP---LYAIDRNDGIHNK-DSQMKQDLTLGDGMPTTVPSEHENAEV
Tc0010475350894510	SKWVVHMVLREMNDNP---LYAIDRNDRIHNN-NG-LMLDATLGDEVLMTVPPGYESFEV
Tc00104705350863740	SKWVVHMVLREMNDNP---LYAIDRNDRIHNN-NG-LMLDATFGDEVLMNVPPGYESFEV
Tc00104705351112960	SKWVVHMVLREVKREP---LYAIDGNDGIYNN-NGQMLRSLTLGDEVVMTVPSGYESFEV
Tc00104705351191190	SKWVVHMVLREVKREP---LYAIDGNDGIYNNDNSQMLRSLTLGDEVLMTVPSGYENFEV
Tc00104705351024930	SKWVVHMVLREMNDSP---LYAIDGNDGIHNN-NSQMLRSLTLGDEVLTTVPSGYGNFEV
Tc00104705350735910	SKWVVHMVLREVKREP---LYAIDRNDGIHND-ASQMKEDSTLGDGMPMTVSSGHENAEV
TvY486_0201870	GEKVTPMVLRESAGAPLCVVAPVGDGDAYYAD--------------VPPFVPSHADGAGY
TvY486_0600050	GEKVTPMVLRESAGAPLCVVAPVGDGDAYYAD--------------VPPFVPSHADGAGY
TvY486_0028480	GEKVTPMVLRESAGAPLCVVAPVGDGDAYYAD--------------VPPFVPSHADGMWL
TvY486_0201850	GEKVTPMVLRESFHVP-YMVGTANDTGAYYAD--------------APPFVPPQNRGMER
Tbg97223580	KKEIIPMTVREVVGEP---LFVLESTPFVPPE-----------GFAAPPLALQYYNQMRT
Tbg97223450	--
Tbg97223390	KKEIIPMTLREVVGEP---LFVLEPTPFVPPE-----------GFAAPPLALQYYNQMRT
Tbg97223430	KKEIIPMTLREVVGEP---LFVLEPTPFVPPE-----------GFAAPPLALQYYNQMRT
Tbg97223760	KKEIIPMTLREVVGEP---LFVLEPTPFVPPE-----------GFAAPPLALQYYNQMRT
Tbg97223550	KKEIIPMTLREVVGEP---LFVLEPTPFVPPE-----------GFAAPPLALQYYNQMRT
Tbg97223410	KKEIIPMTLREVVGEP---LFVLEPTPFVPPE-----------GFAAPPLALQYYNQMRT
Tbg97223680	KKEIIPMTLREVVGEP---LFVLEPTPFVPPE-----------GFAAPPLALQYYNQMRT
Tbg97223740	KKEIIPMTLREVVGEP---LFVLEPTPFVPPE-----------GFAAPPLALQYYNQMRT
Tbg97223540	KKEIIPMTLREVVG--
Tb92725360	KKEIIPMTLREVVGEP---LFVLEPTPFVPPE-----------GFAAPPLALQYYNQMRT
Tb92725340	KKEIIPMTLREVVGEP---LFVLEPTPFVPPE-----------GFAAPPLALQYYNQMRT
Tb92725330	KKEIIPMTLREVVGEP---LFVLEPTPFVPPE-----------GFAAPPLALQYYNQMRT
Tbg97223600	KKEIIPMTLREVVGEP---LFVLEPTPFVPPE-----------GFAAPPLALQYYNQMRT
Tbg97223520	KKEIIPMTLREVVGEP---LFVLEPTPFVPPE-----------GFAAPPLALQYYNQMRT
Tbg97223370	KKEIIPMTLREVVGEP---LFVLEPTPFVPPE-----------GFAAPPLALQYYNQMRT
Tbg97223470	KKEIIPMTLREVVGEP---LFVLEPTPFVPPE-----------GFAAPPLALQYYNQMRT
Tbg97223700	KKEIIPMTLREVVGEP---LFVLEPTPFVPPE-----------GFAAPPLALQYYNQMRT
Tbg97223620	KKEIIPMTLREVVGEP---LFVLEPTPFVPPE-----------GFAAPPLALQYYNQMRT
Tbg97223490	KKEIIPMTLREVVGEP---LFVLESTPFVPPE-----------GFDAPPLALQYYD----
Tbg97223660	KKEIIPMTLREVVGEP---LFVLESTPFVPPE-----------GFAAPPLALQYYNQMRT
Tbg97223640	KKEIIPMTLREVVGEP---LFVLEPTPFVPPE-----------GFAAPPLALQYYNQMRT
Tb92725350	KKEIIPMTLREVVGEP---LFVLEPTPFVPPE-----------GFAAPPLALQYYNQMRT
Tbg97223330	KKEIIPMTLREVVGEP---LFVLESTPFAPPE-----------GFAAPPLALQYYNQMRT
Tb92725290	KKEIIPMTLREVVGEP---LFVLEPTPFVPPE-----------GFAAPPLALQYYNQMRT
Tbg97223720	KKEIIPMTLREVVGEP---LFVLEPTPFVPPE-----------GFAAPPLALQYYNQMRT
Tb92725320	KKEIIPMTLREVVGEP---LFVLEPTPFVPPE-----------GFDAPPLALQYYNQMRT
Tbg97223350	KKEIIPMTLREVVGEP---LFVLESTPFVPPE-----------GFAAPPLALQYYNQMRT
Tbg97223500	KKEIIPMTLREVVGEP---LFVLESTPFVPPE-----------GFAAPPLALQYYNQMRT
Tb92725300	KKEIIPMTLREVVGEP---LFVLEPTPFVPPE-----------GFAAPPLALQYYNQMRT
Tb92725310	KKEIIPMTLREVVGEP---LFVLEPTPFAPPE-----------GFAAPPLALQYYNQMRT
Tbg97222800	KKEIIPMTLREVVGEP---LFVLEPTPFVPPE-----------GFAAPPLALQYYNQMMT
Tb92724760	KKEIIPMTLREVVGEP---LFVLEPTPFVPPE-----------GFAAPPLALQYYNQMMT
Tbg97222940	KKEIIPMTLREVVGEP---LFVLESTPFVPPE-----------GFDAPPLALQYYD----
Tb92724920	KKEIIPMTLREVVGEP---LFVLEPTPFAPPE-----------GFDAPPLALQYYG----
TcIL30002110	GKELTPMTLREVTGLP---VRAVEPESYAAPD-----------EAYDIPQPLGYHYAQGN
TcIL3000013740	GKELTPMTLREVTGLP---VRAVEPESYAAPD-----------EAYDIPQPLGYHYAQGN
TcIL3000027940	GKELTPMTLREVTGLP---VRAVEPESYAAPD-----------EAYDIPQPLGYHYAQGN
TcIL3000001110	GKELTPMTLREVCGLP---LCAADSESYAAPD-----------EAYDIPQPLSYHNAQGH
TcIL3000033420	GKELTPMTLREVCGLP---LCAAGSESYAAPD-----------EAYGIPPPQSYHNAQGH
Tb9276380	--
Tbg972630	--
Tb9276370	--
TcIL3000050510	EKEDTPLSLREVYSPA---ECPSPVEAPLTSE-----------GGGAAEGGSPNASPAVA
TcIL3000050500	EKEDAPLSLREVYSPA---ECPSPVEAPLTSE-----------GGGAAEGGSPNASPAVA
TcIL3000016620	EKEDAPLSLREVYSPA---ECPSPVEAPLTSE-----------GGGAAEGGSPNASPVVA
TcIL3000016630	EKEDAPLSLREVYSPA---ECPSPVEAPLTSE-----------GGGAAEGGSPNASPAVA
TcIL3000047260	DKEITCMRLRETFLSP---ACLSPAEVPLTNG-----------GGGVAKAGSPNASPMGA

Tbg97291330	AADE--------------
Tbg97291280	AADE--------------
Tbg97291270	AGDE--------------
Tbg97291300	AGDE--------------
Tb09v40146	AVDE--------------
Tb09v40147	AADE--------------
Tb091601520	AADE--------------
Tb09v40149	AADE--------------
Tb09v40059	AADE--------------

Tb09v40058	AADE--------------
Tb09v40148	AVDE--------------
Tbg97291380	AGDE--------------
Tb09v40060	AADE--------------
Tbg97291360	AADE--------------
Tc00104705351105310	PRVIKNQETTVYDDGDVL
Tc00104705350763120	PRVIKNQETTVYDDGDVL
Tc00104705350401180	PRVIKNQETTVYDDGDVF
Tc001047053511859200	PRVIKNQENTVYDDGDVF
Tc00104705350601110	PRVIKNQENTVYDDGDVF
Tc00104705351104770	PKVIKNE-----------
Tc00104705350453350	PTVIASQESTVYDLGDVF
Tc001047053508735100	PTVIASQESTVYDLGDIL
Tc0010475350894510	PTVIKNQGSTVYDDGDVA
Tc00104705350863740	PTVIKNQGSTVYDDGDVF
Tc00104705351112960	PTVIKNQGSTVYDDGDVV
Tc00104705351191190	PKVVKNQGSTVYDDGDVA
Tc00104705351024930	PKVIKNQGSTVYDDGDVA
Tc00104705350735910	PKVTASQESTVYDFGDIL
TvY486_0201870	DGAW--------------
TvY486_0600050	DGAW--------------
TvY486_0028480	PQC---------------
TvY486_0201850	VHAW--------------
Tbg97223580	SE----------------
Tbg97223450	------------------
Tbg97223390	SE----------------
Tbg97223430	SE----------------
Tbg97223760	SE----------------
Tbg97223550	SE----------------
Tbg97223410	SE----------------
Tbg97223680	SE----------------
Tbg97223740	SE----------------
Tbg97223540	------------------
Tb92725360	SE----------------
Tb92725340	SE----------------
Tb92725330	SE----------------
Tbg97223600	SE----------------
Tbg97223520	SE----------------
Tbg97223370	SE----------------
Tbg97223470	SE----------------
Tbg97223700	SE----------------
Tbg97223620	SE----------------
Tbg97223490	------------------
Tbg97223660	SE----------------
Tbg97223640	SE----------------
Tb92725350	SE----------------
Tbg97223330	SE----------------
Tb92725290	SE----------------
Tbg97223720	SE----------------
Tb92725320	SE----------------
Tbg97223350	SE----------------
Tbg97223500	SE----------------
Tb92725300	SE----------------
Tb92725310	SE----------------
Tbg97222800	GQSY--------------
Tb92724760	GQSY--------------
Tbg97222940	------------------
Tb92724920	------------------
TcIL30002110	GDEW--------------
TcIL3000013740	GDEW--------------
TcIL3000027940	GDEW--------------
TcIL3000001110	EGQW--------------
TcIL3000033420	EGQW--------------
Tb9276380	------------------
Tbg972630	------------------
Tb9276370	------------------
TcIL3000050510	Q-----------------
TcIL3000050500	Q-----------------
TcIL3000016620	Q-----------------
TcIL3000016630	Q-----------------
TcIL3000047260	R-----------------

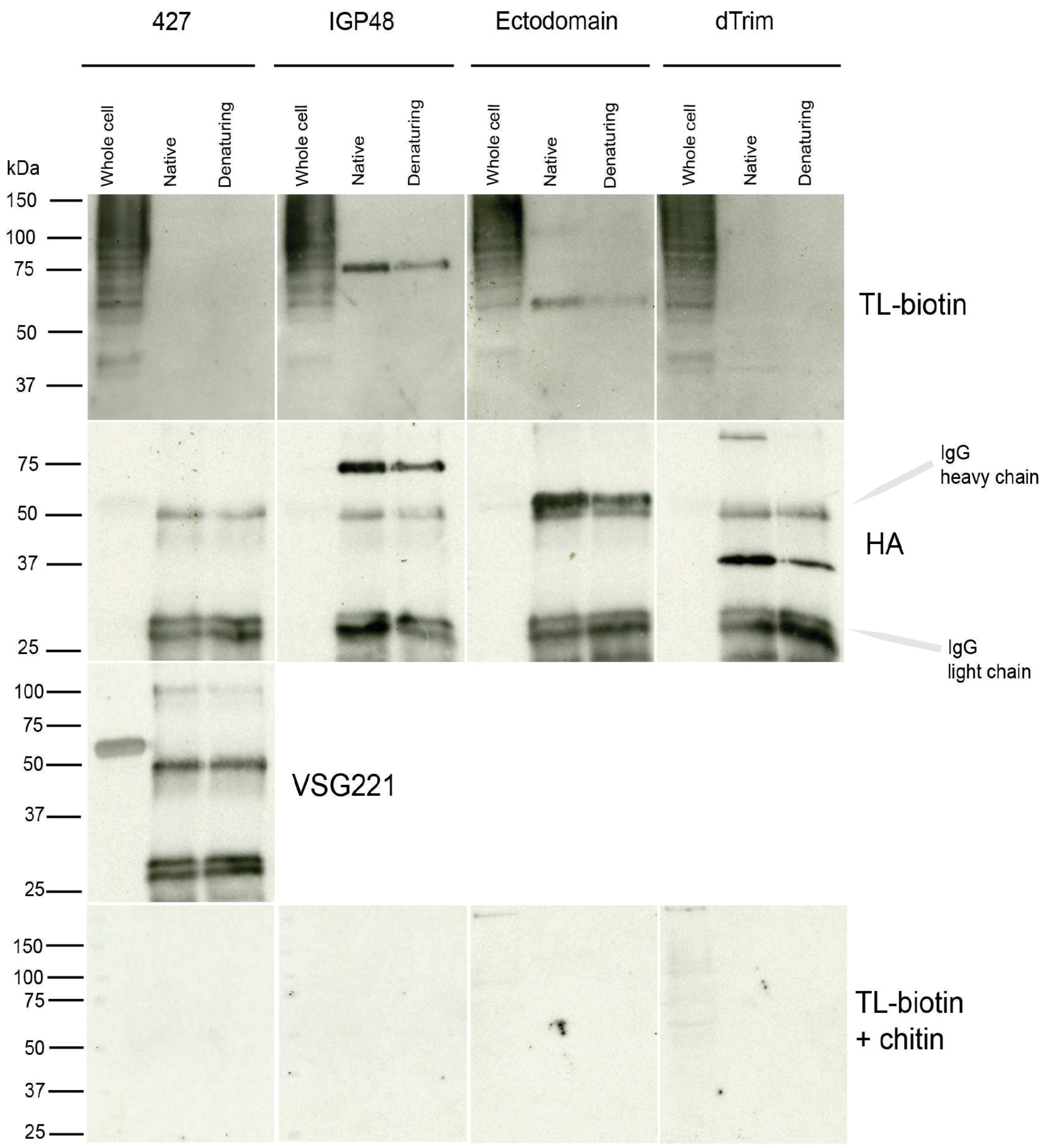
427
IGP48
Ectodomain
dTrim
Whole cell
Native
Denaturing
kDa
150
100
75
50
37
25
TL-biotin
HA
IgG heavy chain
IgG light chain
VSG221
TL-biotin + chitin

Figure S4

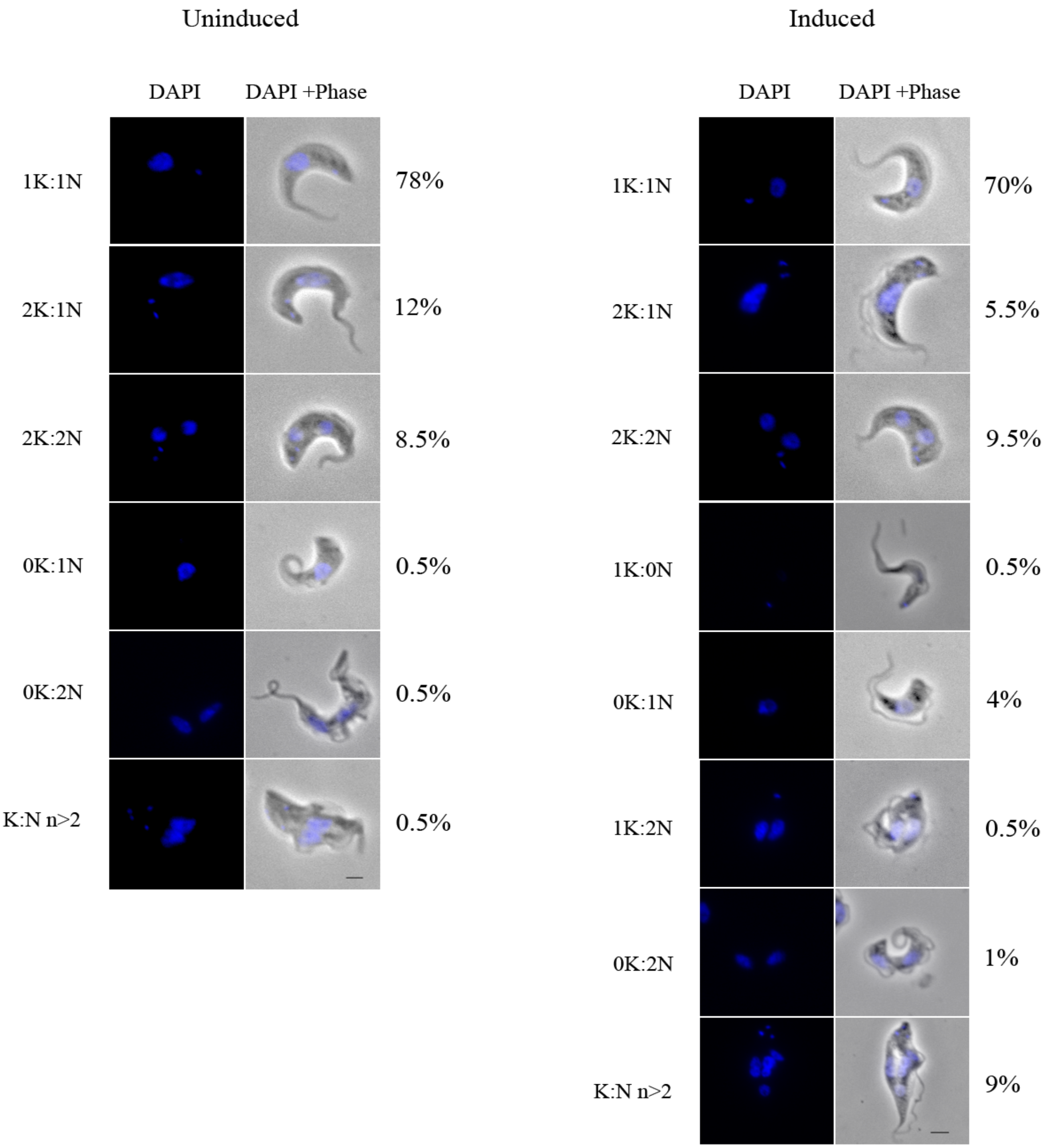
Uninduced
DAPI
DAPI +Phase
1K:1N
78%
2K:1N
12%
2K:2N
8.5%
0K:1N
0.5%
0K:2N
0.5%
K:N n>2
0.5%
Induced
DAPI
DAPI +Phase
1K:1N
70%
2K:1N
5.5%
2K:2N
9.5%
1K:0N
0.5%
0K:1N
4%
1K:2N
0.5%
0K:2N
1%
K:N n>2
9%

Figure S5

Figure S6

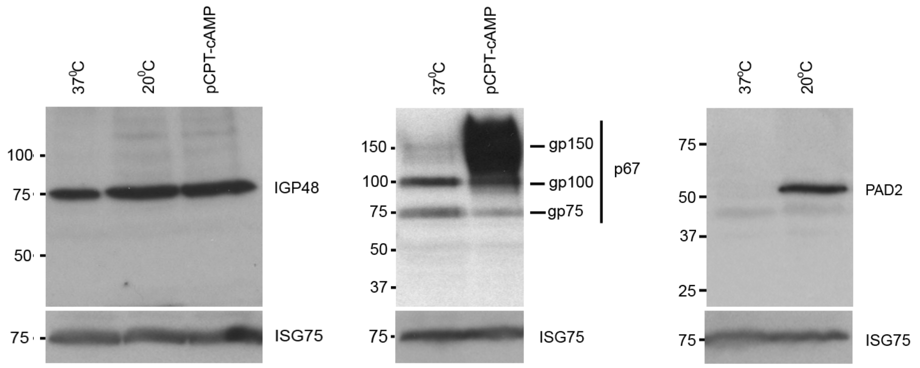

37⁰C
20⁰C
pCPT-cAMP
100
75
50
IGP48
75
ISG75
37⁰C
pCPT-cAMP
150
100
75
50
37
gp150
gp100
gp75
p67
75
ISG75
37⁰C
20⁰C
75
50
37
25
PAD2
75
ISG75

Figure S7

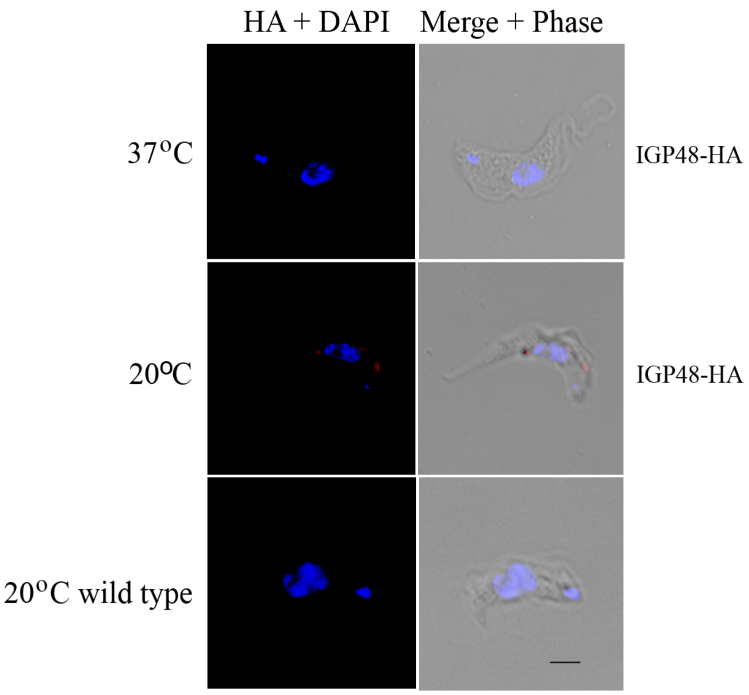

Figure S8

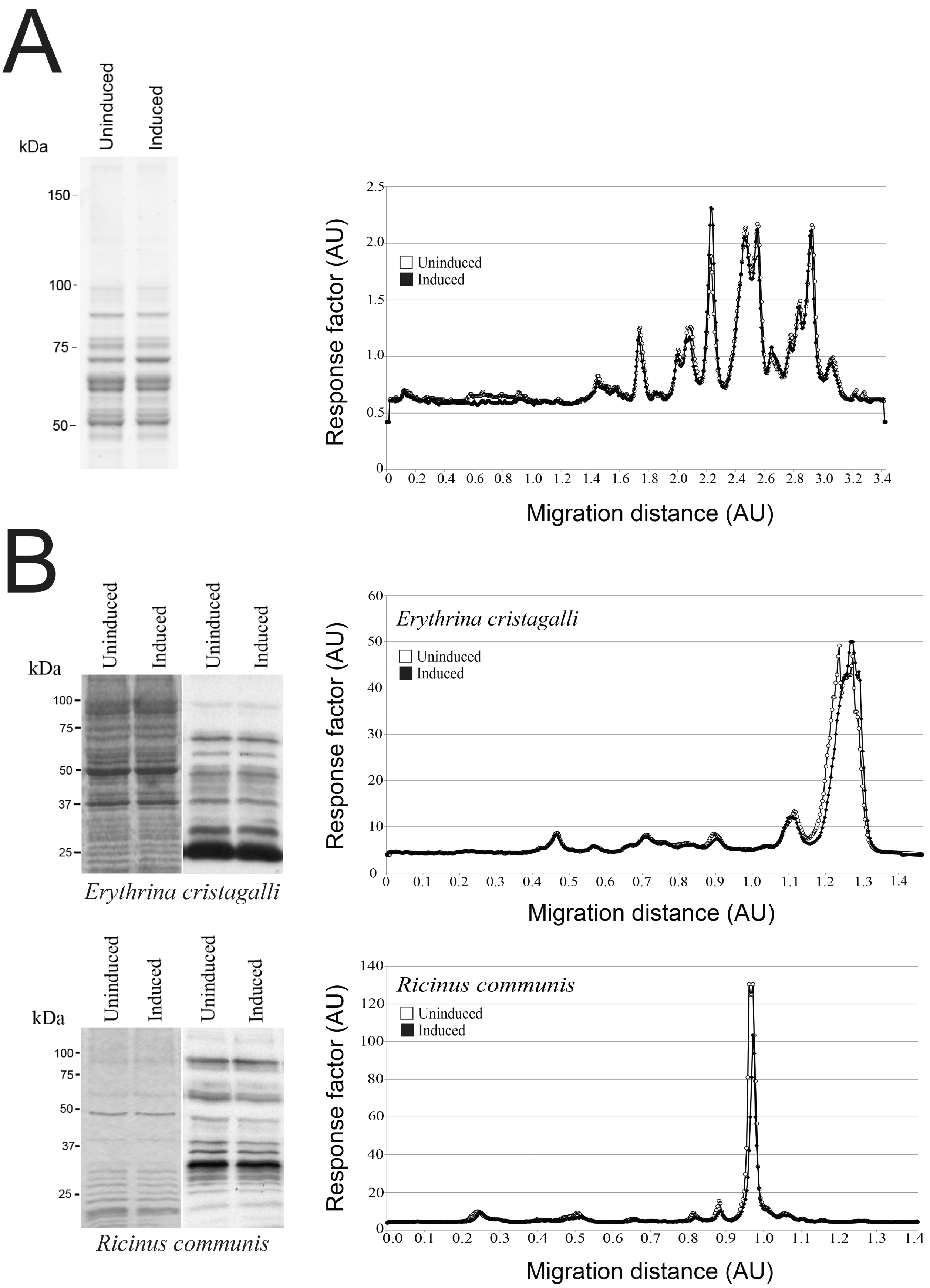
A
Uninduced
Induced
kDa
150
100
75
50
Uninduced
Induced
Response factor (AU)
Migration distance (AU)
0
0.5
1.0
1.5
2.0
2.5
0 0.2 0.4 0.6 0.8 1.0 1.2 1.4 1.6 1.8 2.0 2.2 2.4 2.6 2.8 3.0 3.2 3.4
B
Uninduced
Induced
Uninduced
Induced
kDa
100
75
50
37
25
Erythrina cristagalli
Erythrina cristagalli
Uninduced
Induced
Response factor (AU)
Migration distance (AU)
0
10
20
30
40
50
60
0 0.1 0.2 0.3 0.4 0.5 0.6 0.7 0.8 0.9 1.0 1.1 1.2 1.3 1.4
Uninduced
Induced
Uninduced
Induced
kDa
100
75
50
37
25
Ricinus communis
Ricinus communis
Uninduced
Induced
Response factor (AU)
Migration distance (AU)
0
20
40
60
80
100
120
140
0.0 0.1 0.2 0.3 0.4 0.5 0.6 0.7 0.8 0.9 1.0 1.1 1.2 1.3 1.4

Figure S9

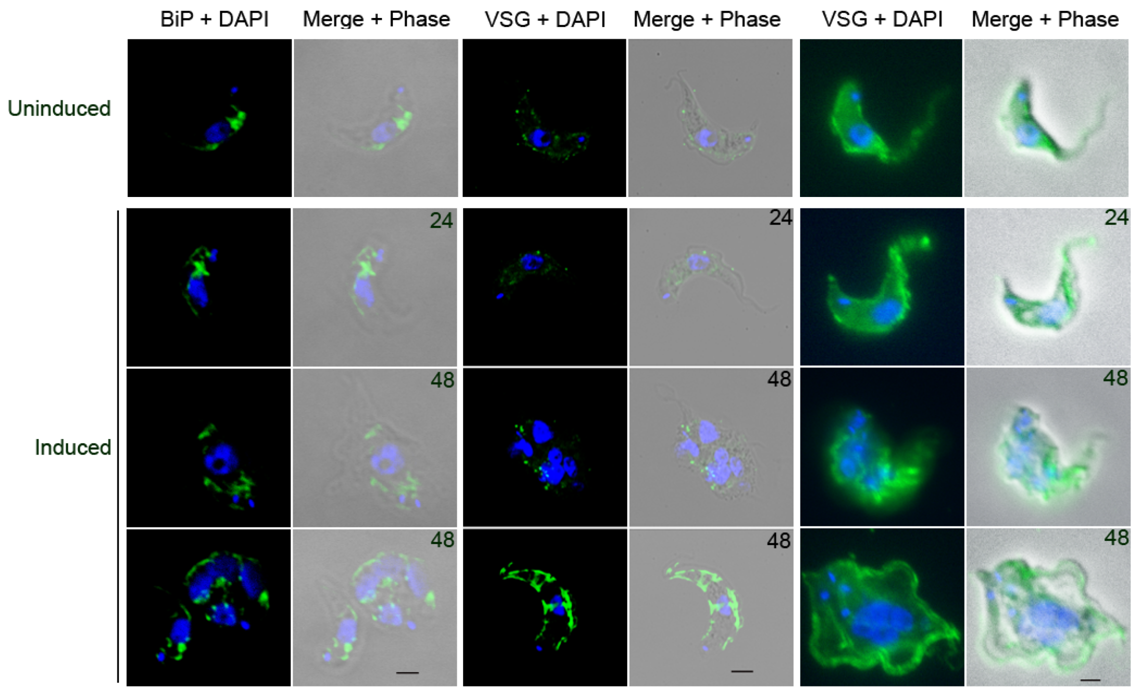

BiP + DAPI
Merge + Phase
VSG + DAPI
Merge + Phase
VSG + DAPI
Merge + Phase
Uninduced
Induced
24
24
24
48
48
48
48
48
48

Figure S10

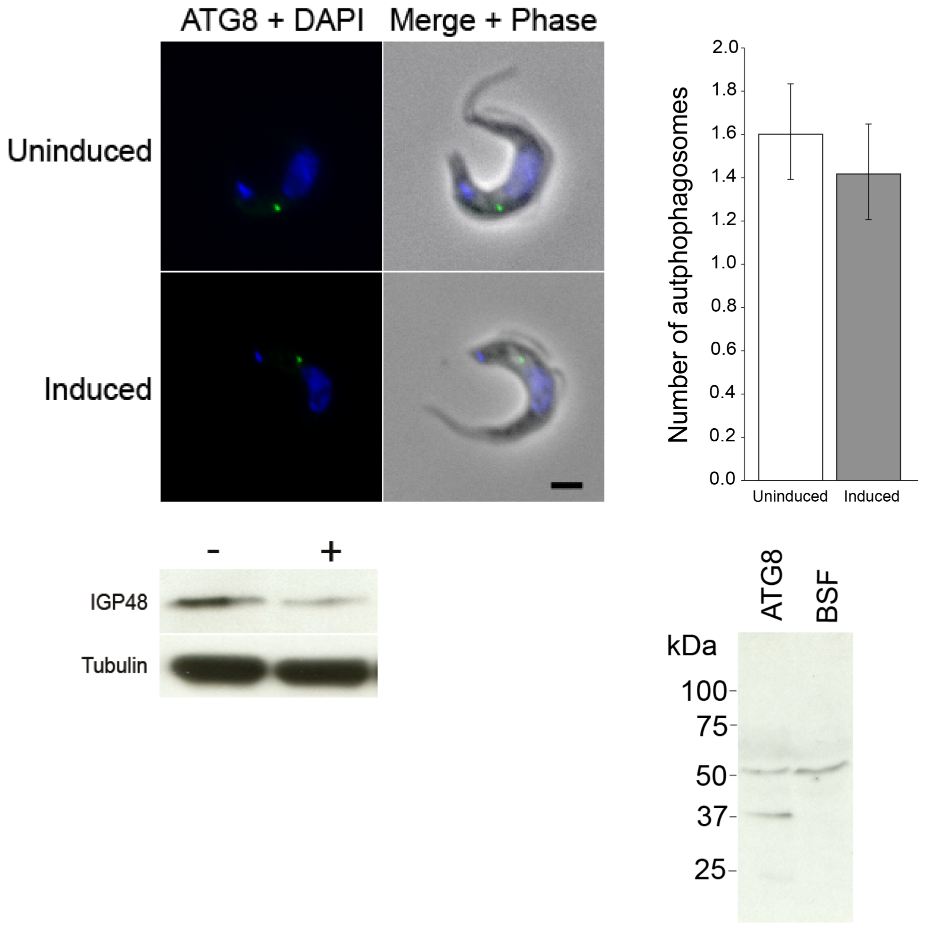

ATG8 + DAPI
Merge + Phase
Uninduced
Induced
Number of autphophagosomes
2.0
1.8
1.6
1.4
1.2
1.0
0.8
0.6
0.4
0.2
0.0
Uninduced
Induced
-
+
IGP48
Tubulin
ATG8
BSF
kDa
100
75
50
37
25

Table S1

Oligomers	Sequence	Restriction site	Epitope tag	
RNAi knock-down confirmation				
IGP48:FP	GCCGGTGCTGCTGTTGTTA	-	-	
IGP48:RP	ATACGGGTGAGGTAACCTTTT	-	-	
IGP40:FP	TTGTGTGTATCGTTGGAATAAG	-	-	
IGP40:RP	AGGATGGGTAACCATTCATTG	-	-	
IGP34:FP	CTCAAGATCAACCTTCGGCAA	-	-	
IGP34:RP	GCACACAATTGCGAGGGAAA	-	-	
			-	
Localisation			-	
IGP48-CO_2H:FP	CTCGAA**AAGCTT**ATGGAAATCCGCATGGTGCCA	HindIII	-	
IGP48-CO_2H:RP (HA)	TGCTAT**CCCGGG**TCAAGCGTAATCTGGAACATCGTATGGGTACTCATCAGCTGCTGGAGT	SmaI	HA	
IGP48-CO_2H:RP (FLAG)	AGCCAT**GGATCC**TCACTTGTCGTCATCGTCTTTGTAGTCCTCATCAGCTGCTGGAGTTTC	BamHI	FLAG	
IGP48-65:RP	ACTATCAAAGTCGGCACCACCGCCACTTCCCTC	-	-	
48-ISG65:FP	GGCGGTGGTGCCGACTTTGATAGTCTCCTAGAT	-	-	
ISG65:RP	GCCACC**CCCGGG**TCAAGCGTAATCTGGAACATCGTATGGGTACATTACTACTTTTACGCTAGA	SmaI	HA	
BiP-IGP48:FP	GCGGCC**GCTAGC**AGTAATGAAGCATCAGATAAG	NheI	-	
BiP-IGP48:RP	TGCCTT**GAATTC**TCACTCATCAGCTGCTGG	EcoRI	-	
IGP40-CO_2H:FP	GCTATG**AAGCTT**ATGAGCATGGACACCCGT	HindIII	-	
IGP40-CO_2H:RP	ACTGCA**CCCGGG**TTAAGCGTAATCTGGAACATCGTATGGGTATTCACTGGTTCTCATTTG	SmaI	HA	
IGP40-65: RP	ACTATCAAAGTCGTGCCAATTACTTCTCTCTTC	-	-	
40-ISG65:FP	AGTAATTGGCACGACTTTGATAGTCTCCTAGAT	-	-	
BiP-IGP40:FP	GCAGAT**GCTAGC**GAAGTACCGGAACCAACTGTG	NheI	-	
BiP-IGP40:RP	GACCTA**GAATTC**TTATTCACTGGTTCTCATTTG	EcoRI	-	
dCLECT:FP	TCTATT**GCTAGC**CCAACTCCGCTTCCACCTGCT	NheI	-	
dCLECT:RP	TGCCTT**GAATTC**TCACTCATCAGCTGCTGG	EcoRI	-	
dTRIM:FP	ATCGTC**GAATTC**GTGCCATCTACAGCTTCCGAT	EcoRI	-	
dTRIM:RP	GTACAT**GAATTC**AGCAGGTGGAAGCGGAGTTGG	EcoRI	-	
	Supplementary Table S1			

Table S2

Accession Number	Description	Accession Number
Tb927.5.390	ISG75	Tb09.v4.0147
Tb927.5.390b	ISG75	Tb09.v4.0146
Tb927.5.360	ISG75	Tb09.v4.0148
Tb927.5.360b	ISG75	Tb09.160.1520
Tb927.5.370	ISG75	Tb09.v4.0149
Tb927.5.370b	ISG75	Tb09.v4.0058
Tb927.5.350b	ISG75	Tb09.v4.0059
Tb927.5.350	ISG75	Tb09.v4.0060
Tb927.5.400	ISG75	
Tb927.5.400b	ISG75	Tb927.2.4920
Tb927.5.380	ISG75	Tb927.2.4760
Tb927.5.380b	ISG75	Tb927.2.5330
		Tb927.2.5340
		Tb927.2.5360
**Accession Number**	**Description**	Tb927.2.5350
Tb927.6.1850	Singleton	Tb927.2.5290
Tb09.160.4230	Singleton	Tb927.2.5300
Tb11.01.4701	Singleton	Tb927.2.5310
Tb927.10.7140	Singleton	Tb927.2.5320
Tb11.01.5310	Singleton	
Tb09.160.1390	Singleton	Tb927.6.380
Tb09.v1.0780	Singleton	Tb927.6.370
Tb927.8.4110	Singleton	
Tb927.8.4060	Singleton	
Tb927.8.4010	Singleton	**Accession Number**
Tb11.01.3590	Singleton	Tb927.5.1430
Tb09.160.3680	Singleton	Tb927.5.1390
Tb927.2.1700	Singleton	Tb927.5.1410
Tb927.2.1760	Singleton	Tb927.5.420b
Tb11.57.0077	Singleton	Tb927.5.420
Tb09.v4.0152	Singleton	Tb927.5.430b
Tb11.1460	Singleton	Tb927.5.430
Tb11.v4.0022	Singleton	Tb927.5.410
Tb927.7.4280	Singleton	Tb927.5.410b
Tb927.7.4230	Singleton	Tb927.5.310

Tb927.7.4260	Singleton	Tb927.5.310b
Tb927.5.440b	Singleton	
Tb927.5.440	Singleton	
Tb09.v1.0510	Singleton	
Tb09.v1.0540	Singleton	
Tb927.7.4270	Singleton	
Tb927.10.6180	Singleton	
Tb11.01.3610	Singleton	
Tb927.5.610	ISG-like	
Tb927.5.620	ISG-like	
Tb927.5.630	ISG-like	
Tb927.10.2360	Singleton	
Tb11.1490	Singleton	
Tb927.10.120	Singleton	
Tb927.3.2870	Singleton	
Tb927.10.11140	Singleton	
Tb09.160.2060	Singleton	
Tb927.1.3900	Singleton	
Tb11.47.0001	Singleton	
Tb927.1.660	Singleton	
Tb927.3.5170	Singleton	
Tb927.1.770	Singleton	
Tb09.v4.0010	Singleton	
Tb927.8.8030	Singleton	
Tb927.8.7280	Singleton	
Tb09.160.4390	Singleton	
Tb927.6.1740	Singleton	
Tb927.1.4770	Singleton	
Tb927.3.4230	Singleton	
Tb09.160.4810	Singleton	
Tb09.v1.0630	Singleton	
Tb927.4.590	Singleton	
Tb927.10.2080	Singleton	
Tb927.8.3120	Singleton	
Tb927.5.1810	Singleton	
Tb11.01.0290	Singleton	

Tb927.8.6380	Singleton	
Tb927.5.4060	Singleton	
Tb927.4.2320	Singleton	
Tb09.160.5140	Singleton	
Tb927.5.2580	Singleton	
Tb11.13.0007	Singleton	
Tb927.10.2420	Singleton	
Tb09.160.2950	Singleton	
Tb09.v1.0650	Singleton	
Tb09.160.2340	Singleton	
Tb927.10.10840	Singleton	
Tb09.211.4290	Singleton	
Tb09.160.2140	Singleton	
Tb927.4.1120	Singleton	
Tb927.10.12450	Singleton	
Tb927.3.840	Singleton	
Tb927.7.5710	Singleton	
Tb11.02.2880	Singleton	
Tb09.160.5040	Singleton	
Tb927.4.1390	Singleton	
Tb11.02.3760	Singleton	
Tb09.211.2890	Singleton	
Tb10.v4.0132	Singleton	
Tb927.10.10760	Singleton	
Tb927.3.2360	Singleton	
Tb927.7.900	Singleton	
Tb09.v4.0150	Singleton	
Tb09.v2.0050	Singleton	
Tb927.5.1020	Singleton	
Tb927.3.3140	Singleton	
Tb927.8.560	Singleton	
Tb927.2.6390	Singleton	
Tb927.4.5470	Singleton	
Tb927.10.13090	Singleton	
Tb927.2.5970	Singleton	
Tb927.5.1740	Singleton	

Tb927.7.470	Singleton	
Tb11.01.1000	Singleton	
Tb09.v1.0850	Singleton	
Tb09.211.4680	Singleton	
Tb927.10.700	Singleton	
Tb11.02.0780	Singleton	
Tb09.211.4770	Singleton	
Tb927.6.4500	Singleton	
Tb11.01.7770	Singleton	
Tb927.4.4920	Singleton	
Tb11.01.3160	Singleton	
Tb927.8.6590	Singleton	
Tb927.4.510	Singleton	
Tb11.01.3790	Singleton	
Tb11.49.0009	Singleton	
Tb11.30.0008	Singleton	
Tb09.v4.0179	Singleton	
Tb10.v4.0122	Singleton	
Tb09.142.0500	Singleton	
Tb11.02.0060	Singleton	
Tb927.10.2500	Singleton	
Tb05.5K5.520	Singleton	
Tb927.10.4670	Singleton	
Tb927.5.292b	Singleton	
Tb927.2.2490	Singleton	
Tb927.7.4690	Singleton	
Tb11.v4.0065	Singleton	
Tb927.4.1190	Singleton	
Tb927.6.3700	Singleton	
Tb927.7.4980	Singleton	
Tb927.10.6640	Singleton	
Tb927.5.1630	Singleton	
Tb927.10.7000	Singleton	
Tb927.10.7180	Singleton	

**Description**	**Accession Number**	**Description**
IGP48	Tb927.2.3300	ISG65
IGP48	Tb927.2.3290	ISG65
IGP48	Tb927.2.3310	ISG65
IGP48	Tb927.2.3280	ISG65
IGP48	Tb927.2.3270	ISG65
IGP48	Tb927.2.3320	ISG65
IGP48		
IGP48		
	**Accession Number**	**Description**
IGP40	Tb927.8.7920	Adenylate Cyclase
IGP40	Tb927.8.7900	Adenylate Cyclase
IGP40	Tb927.8.7890	Adenylate Cyclase
IGP40	Tb927.8.7930	Adenylate Cyclase
IGP40	Tb927.8.7870	Adenylate Cyclase
IGP40	Tb927.8.7940	Adenylate Cyclase
IGP40	Tb927.8.7590	Adenylate Cyclase
IGP40	Tb927.4.4430	Adenylate Cyclase
IGP40	Tb927.4.4450	Adenylate Cyclase
IGP40	Tb927.4.4410	Adenylate Cyclase
	Tb927.4.4440	Adenylate Cyclase
IGP34	Tb927.7.7530	Adenylate Cyclase
IGP34	Tb927.7.7520	Adenylate Cyclase
	Tb927.4.4470	Adenylate Cyclase
	Tb927.4.4460	Adenylate Cyclase
**Description**	Tb927.4.3880	Adenylate Cyclase
ISG-like	Tb927.4.3870	Adenylate Cyclase
ISG-like	Tb927.4.3750	Adenylate Cyclase
ISG-like	Tb927.6.190	Adenylate Cyclase
ISG-like	Tb927.5.650	Adenylate Cyclase
ISG-like	Tb927.7.6040	Adenylate Cyclase
ISG-like	Tb927.7.6080	Adenylate Cyclase
ISG-like	Tb927.7.6070	Adenylate Cyclase
ISG-like	Tb927.7.6060	Adenylate Cyclase
ISG-like	Tb927.7.6050	Adenylate Cyclase
ISG-like	Tb927.10.13040	Adenylate Cyclase

ISG-like	Tb927.7.7470	Adenylate Cyclase
	Tb927.10.16190	Adenylate Cyclase
	Tb927.5.330b	Adenylate Cyclase
	Tb927.5.330	Adenylate Cyclase
	Tb927.5.320b	Adenylate Cyclase
	Tb927.5.320	Adenylate Cyclase
	Tb927.5.285b	Adenylate Cyclase

Table S3: Serum immunoglobulin responses to IGP48 in *T. b. rhodesiense* patients.

	Patient ID	IgG response to rIGP48 (+/-)	IgM response to rIGP48 (+/-)	Disease stage	Age (years)	Parasitaemia	Serum IgG (g/l)	Serum IgM (g/l)
INFECTED								
	L2	-	-	Late	12	0.25	20.5	9.4
	L3	+	-	Late	15	1	30.1	10.2
	L16	+	+	Early	40	20	25.2	21.7
	L30	+	-	Late	18	3	45.3	7.6
	S3	-	-	Late	12	0	17.3	5.7
	S13	+	-	Early	16	50	16.6	1.3
	S48	+	+	Late	40	100	3.1	18.8
	S69	+	+	Early	15	20	51.3	2.7
	S79	+	-	Late	50	2	17.8	51.6
UNINFECTED								
	LC1	-	-	N/A				
	LC15	-	-					
	EUM1	-	-					
	EUF1	-	-					

a: Disease stage: Later stage (meningoencephalitic) criteria either CSF white cells >5/mm^3 or parasites observed in the CSF.
b: Parasitaemia: Number of Giemsa stained parasites observed per 10 fields, at 400x magnification.
